# Supplementary material for: Direct and mediated effects of treatment context on low back pain outcome: a prospective cohort study
Source: BMJ Open. 2021 May 18;11(5):e044831. doi: 10.1136/bmjopen-2020-044831 (PMC8130743; doi:10.1136/bmjopen-2020-044831)
Supplement: Supplementary data [file bmjopen-2020-044831supp001.pdf]

Direct and Mediated Effects of Treatment Context on Back Pain: Supplemental Material

Contents

Table 1: Deviations from Published Protocol 1
Table 2: Study Measures with Example Items, Response Scales, and Interpretation 2
Table 3. Treatment Characteristics by Treatment Group, n (%) 6
Table 4. Practitioner Characteristics at Baseline by Treatment Group 7
Table 5. Secondary Outcomes (Mean, SD) over Time. 8
Table 6. Mean (Standard Deviation) Scores on Mediators over Time by Treatment Group 9
Table 7. Interaction Terms between Contextual Factors and Treatment Type 10
Figure 1. Modified Consort Diagram. Showing Participant Flow, Recruitment and Drop-Out by Therapy Group. 12
Figure 2. Primary Outcomes over Time by Therapy Group 14
Figure 3. General Mediation Model 15
References 16

Table 1: Deviations from Published Protocol

#	Deviation	Rationale
1	Unable to recruit equivalent numbers of practitioners and patients from the NHS and the private sector and therefore did not test the hypothesis that differences in contextual effects between acupuncture, osteopathy, and physiotherapy would be more pronounced in the NHS than the private sector.	Around the time recruitment began there was a marked decline in NHS commissioning of osteopathy and acupuncture for low back pain. This made it very difficult to recruit these groups.
2	Revised sample size calculation.	Necessary to reflect changes in sample as per deviation #1.
3	No test of whether effects of contextual components on back-related disability are mediated by increased patient adherence to theory-specific lifestyle advice (in addition to pain beliefs and self-efficacy).	Extensive missing patient self-report data on adherence and the variety of lifestyle advice precluded a test of this hypothesis.
4	Practitioner-reported data on caseload, waiting list, and appointment duration not collected.	Items were erroneously omitted from the questionnaire.

Bishop F, *et al. BMJ Open* 2021; 11:e044831. doi: 10.1136/bmjopen-2020-044831

Table 2: Study Measures with Example Items, Response Scales, and Interpretation

Construct	Measure (subscale)	Example item	Response scale	Possible range	Cronbach's alpha**	High score indicates
Primary Outcomes						
Disability	RMDQ[1]	I change position frequently to try to get my back comfortable	Yes/No	0-24	-	Greater back-related disability
Secondary Outcomes						
Social Role Disability	Core single item[2]	During the past 4 weeks, how many days did back pain or leg pain (sciatica) keep you from going to work or college?[2]	Free text – number of days in past 4 weeks	0-28	-	Greater social role disability
Functional impairment	Core single item[2]	During the past week, how much did pain interfere with your normal work (including both work outside the home and housework)?[2]	5-point verbal	1-5	-	Greater functional impairment
Pain	Core single item[2]	Please rate your pain by indicating the number that best describes your pain on average in the last 24 hours.[2]	11-point numerical	0-10	-	Greater average pain over last 24 hours
Wellbeing	Core single item[2]	If you had to spend the rest of your life with the symptoms you have right now, how would you feel about it?	5-point verbal	1-5	-	Greater wellbeing
Satisfaction	Core single item[2]	Over the course of treatment for your back pain or leg pain (sciatica) so far, how satisfied are you with your overall health care?	5-point verbal	1-5	-	Greater satisfaction with treatment
Contextual Components						
Therapeutic alliance: task	WAI-SF[3 4] (Task)	I believe the way we are working with my problem is correct	5-point verbal	1-5	0.67	Greater patient-practitioner agreement on task (i.e. how to

						achieve goals)
Therapeutic alliance: goal	WAI-SF[3 4] (Goal)	___ and I are working towards mutually agreed upon goals	5-point verbal	1-5	0.74	Greater patient-practitioner agreement on goals
Therapeutic alliance: bond	WAI-SF[3 4] (Bond)	I believe ___ likes me.	5-point verbal	1-5	0.86	Greater patient-practitioner interpersonal affective bond
Environment: Limits on Treatment*	ABS-mp[5 6] (Limitations on sessions)	If I keep seeing my patients on and off I can prevent relapse	7-point agree/disagree	4-28	0.46	Disinclined to put a limit on number of sessions or refer elsewhere
Environment: Connections within the Healthcare System*	ABS-mp[5 6] (Perceived connections within the health care system)	When referring patients I am confident they will receive good treatment	7-point agree/disagree	3-21	0.49	Feels well-connected to health care system resources
Environment: Satisfaction with appointment systems	PSQ[7] (Appointments)	Getting an appointment at a convenient time is easy	5-point agree/disagree	1-5	0.81	Less satisfied with appointment systems
Environment: Satisfaction with access	PSQ[7] (Access)	It is easy to get advice over the telephone	5-point agree/disagree	1-5	0.80	Less satisfied with access
Environment: Satisfaction with facilities	PSQ[7] (Facilities)	The waiting room is uncomfortable	5-point agree/disagree	1-5	0.75	Less satisfied with facilities
Patient's beliefs: Expectancies for treatment effectiveness	LBP treatment beliefs questionnaire[8] (Effectiveness)	[therapy] can work well for people with back pain	5-point agree/disagree	1-5	0.82	Stronger beliefs that therapy will be effective
Patient's beliefs: Perceived credibility of treatment	LBP treatment beliefs questionnaire [8] (Credibility)	Having [therapy] for back pain makes a lot of sense	5-point agree/disagree	1-5	0.85	Stronger beliefs that therapy is credible
Patient's beliefs:	LBP treatment beliefs	I feel that [therapy] would not	5-point	1-5	0.83	Fewer concerns about

Concerns about treatment	questionnaire [8] (Concerns)	harm me	agree/disagree			therapy
Patient's beliefs: Individualised fit of treatment	LBP treatment beliefs questionnaire [8] (individual fit)	I am confident [therapy] would be a suitable treatment for my back pain	5-point agree/disagree	1-5	0.87	Stronger beliefs that therapy is good fit for oneself
Practitioner's beliefs: psychological*	ABS-mp [5 6] (Psychological)	I often find myself providing psychological support to patients	7-point agree/disagree	4-28	0.65	More willing to engage with patients' psychological issues
Practitioner's beliefs: confidence *	ABS-mp[5 6] (Confidence and concern)	I am concerned about the quality of treatment my referred patients receive	7-point agree/disagree	2-14	0.18	More confident and less concerned about one's own and others' clinical limitations
Practitioner's beliefs: reactivation*	ABS-mp[5 6] (Reactivation)	Return to normal daily activities is the most important consequence of treatment	7-point agree/disagree	3-21	0.57	Greater tendency to focus on re-activation of work and activity
Practitioner's beliefs: biomedical*	ABS-mp[5 6] (Biomedical)	If you look hard enough you can find a structural reason for most patients' back pain	7-point agree/disagree	3-21	0.57	Greater tendency to focus on biomedical model of back pain (also includes rest and vigilance)
Practitioner's beliefs: Outcome Expectancies*	Single item per patient	Thinking about this patient, how effective do you expect your course of treatment to be?	7-point numerical	1-7	-	Expects treatment to be extremely effective for this patient
Hypothesised Mediators						
Self-efficacy for pain management	Self-efficacy for pain management[9]	How certain are you that you can decrease your pain quite a bit?	10-point numerical	10-100	0.78	Higher self-efficacy for pain management
Perception of LBP as threatening	Brief IPQ[10]	How much does your back pain affect your life?	11-point numerical	0-80	0.73	LBP perceived as more threatening
Psychosocial distress	StartBack[11]	I feel that my back pain is terrible and it's never going to get any better	2-point agree/disagree	0-5	0.45	Greater psychosocial distress

* These questionnaires completed by practitioners. All others completed by patients.

Bishop F, *et al. BMJ Open* 2021; 11:e044831. doi: 10.1136/bmjopen-2020-044831

** Cronbach's alpha in this sample

RMDQ, Roland Morris Disability Questionnaire. WAI-SF, Working Alliance Inventory – Short Form. ABS-mp, Attitudes to Back Pain Scale – Musculoskeletal Practitioners. PSQ, Patient Satisfaction Questionnaire. LBP, Low Back Pain. IPQ, Illness Perceptions Questionnaire.

Table 3. Treatment Characteristics by Treatment Group, n (%)

	Physiotherapy NHS (n=196)	Physiotherapy Private (n=165)	Osteopathy (n=394)	Acupuncture (n=205)
Number of weeks treated: M(SD)	6.9 (5.3)	6.1 (9.8)	4.3 (4.6)	8.6 (10.9)
Number of appointments attended: M (SD)	3.9 (2.7)	4.0 (3.2)	3.2 (2.3)	5.1 (4.2)
Treatment Modalities (>1 permitted)				
Manual therapy	61 (31.1%)	116 (70.3%)	248 (62.9%)	50 (24.4%)
Electro/thermal therapy	7 (3.6%)	30 (18.2%)	45 (11.4%)	59 (28.8%)
Acupuncture	21 (10.7%)	18 (10.9%)	46 (11.7%)	123 (60.0%)
Exercise	139 (70.9%)	117 (70.9%)	128 (32.5%)	16 (7.8%)
Lifestyle advice	127 (64.8%)	112 (67.9%)	205 (52.0%)	56 (27.3%)
Missing data	45 (23.0%)	44 (26.7%)	93 (23.6%)	80 (39.0%)

NHS, National Health Service. M, Mean. SD, Standard Deviation.

Note. The average duration of treatment was longest for patients seeing acupuncturists and shortest for patients seeing osteopaths. There was considerable missing data on the specific treatment modalities used for each patient (particularly for the acupuncture group). Among those patients for whom treatment modalities were reported, manual therapy techniques were most commonly used with patients consulting osteopaths and physiotherapists in the private sector; electro/thermal and acupuncture techniques were most likely to be applied by acupuncturists but were used for a minority of patients in the other groups; and patients consulting physiotherapists were most likely to undertake specific exercises or be given lifestyle advice. Categories were defined as follows: manual therapy (specifically, one or more of: mobilisation, articulation, grade V manipulation, high-velocity low-amplitude technique, soft tissue techniques, and East Asian techniques such as acupressure, tuina, gua sha, cupping); electro-therapy therapy (transcutaneous electrical nerve stimulation, ultrasound, electro-acupuncture, application of heat, application of cold, moxibustion); acupuncture (western acupuncture such as trigger point needling and segmental needling, traditionally-based acupuncture such as Traditional Chinese Medicine, five elements, stems and branches, Japanese needling); exercise (individual, group, hydrotherapy); lifestyle advice (therapy-specific and non-therapy-specific advice on diet, specific exercises, physical activity, rest, and relaxation).

Table 4. Practitioner Characteristics at Baseline by Treatment Group

	Whole sample (n=166)	Physiotherapy NHS (n=36)	Physiotherapy Private (n=29)	Osteopathy (n=46)	Acupuncture (n=55)
Years since qualifying	20.6 (10.1)	15.5 (7.2)	25.3 (11.0)	24.0 (10.9)	18.5 (8.6)
Self-rated experience treating LBP*	4.3 (0.8)	3.8 (1.3)	4.5 (0.9)	4.5 (0.7)	4.2 (0.7)
Age	49.7 (10.8)	39.2 (8.3)	50.8 (8.3)	55.0 (10.6)	53.3 (14.6)

* Rated on a 1-5 scale where 1 = not at all experienced; 5 = very experienced.

NHS, National Health Service. LBP, Low Back Pain.

Bishop F, *et al. BMJ Open* 2021; 11:e044831. doi: 10.1136/bmjopen-2020-044831

Table 5. Secondary Outcomes (Mean, SD) over Time.

Secondary Outcome	Baseline	2-weeks	3-months
Social Role Disability	2.0 (5.4)	N/A	0.9 (4.2)
Functional impairment	3.2 (1.1)	2.5 (1.1)	2.2 (1.1)
Pain	5.0 (2.2)	3.5 (2.4)	3.4 (2.5)
Wellbeing	1.6 (0.9)	2.3 (1.3)	2.8 (1.4)
Satisfaction	4.1 (1.0)	4.1 (1.0)	4.1 (1.1)

Bishop F, *et al. BMJ Open* 2021; 11:e044831. doi: 10.1136/bmjopen-2020-044831

Table 6. Mean (Standard Deviation) Scores on Mediators over Time by Treatment Group

		Self-efficacy for coping with pain			Perception of LBP as threatening			Psychosocial complexity of LBP		
Treatment Group / Timepoint		Baseline	2-weeks	3-months	Baseline	2-weeks	3-months	Baseline	2-weeks	3-months
Whole sample		68.5 (19.7)	65.9 (25.3)	69.1 (24.1)	37.2 (9.9)	32.7 (13.2)	32.1 (14.5)	0.60 (0.7)	1.1 (1.8)	1.1 (1.9)
Physiotherapy	NHS	60.6 (19.2)	56.2 (24.0)	59.0 (24.3)	41.0 (9.5)	38.9 (11.8)	36.8 (13.9)	0.6 (0.6)	1.3 (1.9)	1.4 (1.9)
Physiotherapy	Private	74.8 (23.0)	71.4 (26.6)	75.3 (24.6)	34.3 (9.4)	27.9 (13.4)	26.8 (14.0)	0.6 (0.6)	0.8 (1.6)	1.0 (1.9)
Osteopathy		70.0 (17.9)	66.8 (26.0)	71.4 (23.7)	35.4 (9.7)	30.7 (12.9)	30.0 (13.9)	0.6 (0.7)	1.1 (1.9)	1.0 (1.9)
Acupuncture		68.2 (18.3)	68.2 (21.9)	68.8 (21.7)	39.8 (9.4)	35.2 (12.4)	35.5 (14.5)	0.6 (0.7)	1.0 (1.7)	1.3 (1.9)

Bishop F, et al. BMJ Open 2021; 11:e044831. doi: 10.1136/bmjopen-2020-044831

Table 7. Interaction Terms between Contextual Factors and Treatment Type

Contextual component	Interaction term (95% CI)<br><br>Osteopathy*	Interaction term (95% CI)<br><br>Acupuncture*
Therapeutic alliance: goal	-0.27 (-1.62, 1.09)	0.88 (-0.79, 2.55)
Therapeutic alliance: task	-0.32 (-1.64, 1.00)	1.26 (-0.41, 2.93)
Therapeutic alliance: bond	0.16 (-0.83, 1.15)	0.55 (-0.78, 1.89)
Environment: Limits on Treatment	-0.13 (-0.60, 0.34)	0.14 (-0.33, 0.62)
Environment: Connections within the Healthcare System	0.12 (-0.35, 0.59)	0.33 (-0.24, 0.91)
Environment: Satisfaction with appointment systems	-0.07 (-0.22, 0.36)	-0.20 (-0.68, 0.08)
Environment: Satisfaction with access	-0.15 (-0.36, 0.07)	-0.31 (-0.47, -0.06)
Environment: Satisfaction with facilities	-0.13 (-0.42, 0.16)	-0.46 (-0.82, 0.09)
Patient's beliefs: Perceived credibility of treatment	1.43 (-1.43, 4.30)	0.58 (-2.26, 3.43)
Patient's beliefs: Expectancies for treatment effectiveness	2.86 (-0.08, 5.80)	1.85 (-1.13, 4.83)
Patient's beliefs: Concerns about treatment	0.04 (-2.85, 2.92)	-0.53 (-3.14, 2.09)
Patient's beliefs: Individualised fit of treatment	0.58 (-2.46, 3.62)	1.92 (-1.13, 4.97)
Practitioner's beliefs: Psychological	-0.46 (-0.87, 0.04)	-0.43 (-1.08, 0.21)
Practitioner's beliefs: Confidence	-0.34 (-1.01, 0.33)	-0.19 (-0.94, 0.55)

Bishop F, *et al. BMJ Open* 2021; 11:e044831. doi: 10.1136/bmjopen-2020-044831

Practitioner's beliefs: Reactivation	-0.17 (-0.57, 0.23)	0.18 (-0.24, 0.60)
Practitioner's beliefs: Biomedical	0.09 (-0.25, 0.43)	-4.10 (-9.50, 1.33)
Practitioner's beliefs: Outcome expectancies	0.22 (-0.61, 1.04)	0.37 (-0.61, 1.36)

*Physiotherapy is the reference group in the categorical treatment variable

Figure 1. Modified Consort Diagram. Showing Participant Flow, Recruitment and Drop-Out by Therapy Group.

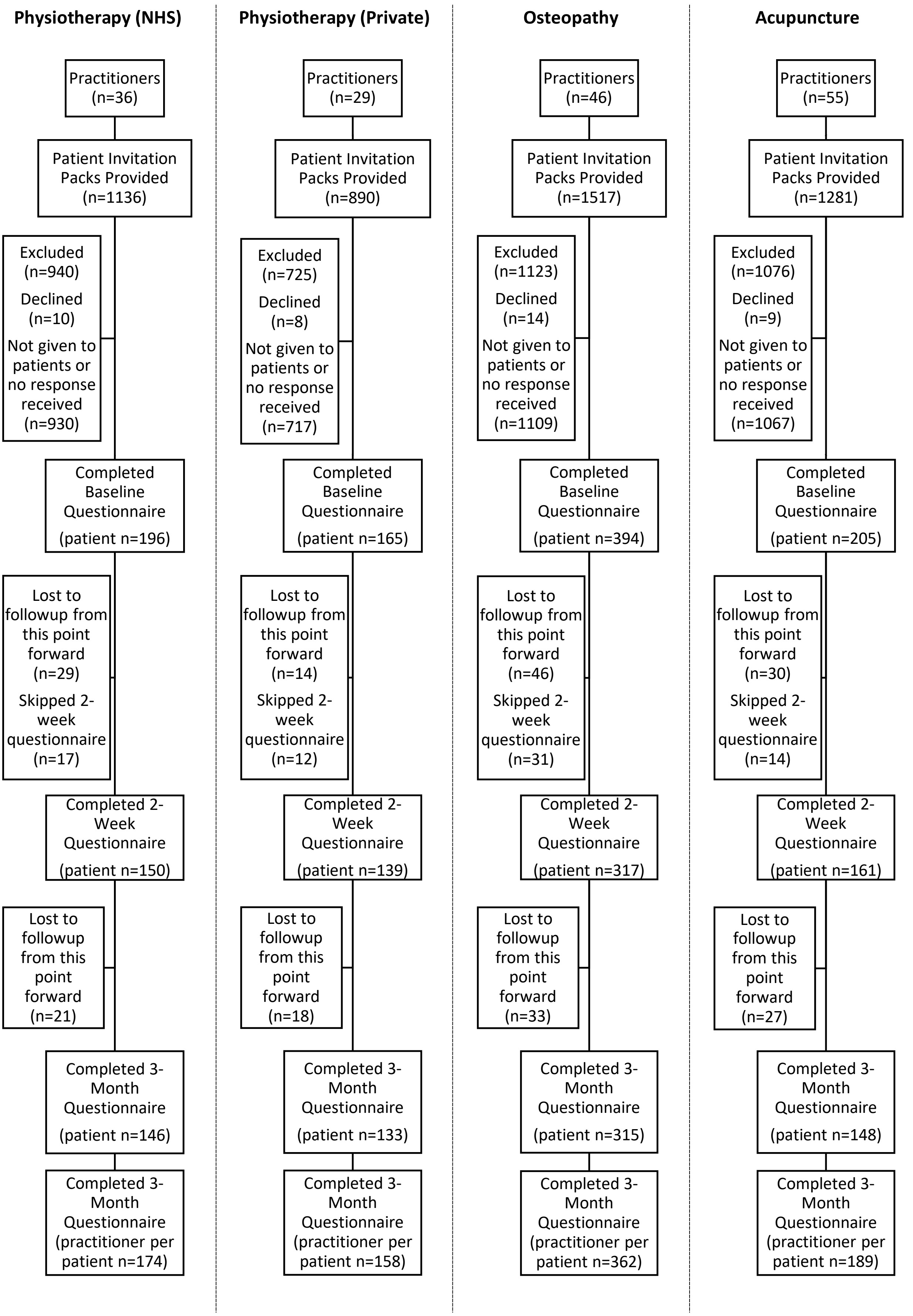
Physiotherapy (NHS)
Practitioners (n=36)
Patient Invitation Packs Provided (n=1136)
Excluded (n=940)
Declined (n=10)
Not given to patients or no response received (n=930)
Completed Baseline Questionnaire (patient n=196)
Lost to followup from this point forward (n=29)
Skipped 2-week questionnaire (n=17)
Completed 2-Week Questionnaire (patient n=150)
Lost to followup from this point forward (n=21)
Completed 3-Month Questionnaire (patient n=146)
Completed 3-Month Questionnaire (practitioner per patient n=174)
Physiotherapy (Private)
Practitioners (n=29)
Patient Invitation Packs Provided (n=890)
Excluded (n=725)
Declined (n=8)
Not given to patients or no response received (n=717)
Completed Baseline Questionnaire (patient n=165)
Lost to followup from this point forward (n=14)
Skipped 2-week questionnaire (n=12)
Completed 2-Week Questionnaire (patient n=139)
Lost to followup from this point forward (n=18)
Completed 3-Month Questionnaire (patient n=133)
Completed 3-Month Questionnaire (practitioner per patient n=158)
Osteopathy
Practitioners (n=46)
Patient Invitation Packs Provided (n=1517)
Excluded (n=1123)
Declined (n=14)
Not given to patients or no response received (n=1109)
Completed Baseline Questionnaire (patient n=394)
Lost to followup from this point forward (n=46)
Skipped 2-week questionnaire (n=31)
Completed 2-Week Questionnaire (patient n=317)
Lost to followup from this point forward (n=33)
Completed 3-Month Questionnaire (patient n=315)
Completed 3-Month Questionnaire (practitioner per patient n=362)
Acupuncture
Practitioners (n=55)
Patient Invitation Packs Provided (n=1281)
Excluded (n=1076)
Declined (n=9)
Not given to patients or no response received (n=1067)
Completed Baseline Questionnaire (patient n=205)
Lost to followup from this point forward (n=30)
Skipped 2-week questionnaire (n=14)
Completed 2-Week Questionnaire (patient n=161)
Lost to followup from this point forward (n=27)
Completed 3-Month Questionnaire (patient n=148)
Completed 3-Month Questionnaire (practitioner per patient n=189)

Figure 2. Primary Outcomes over Time by Therapy Group

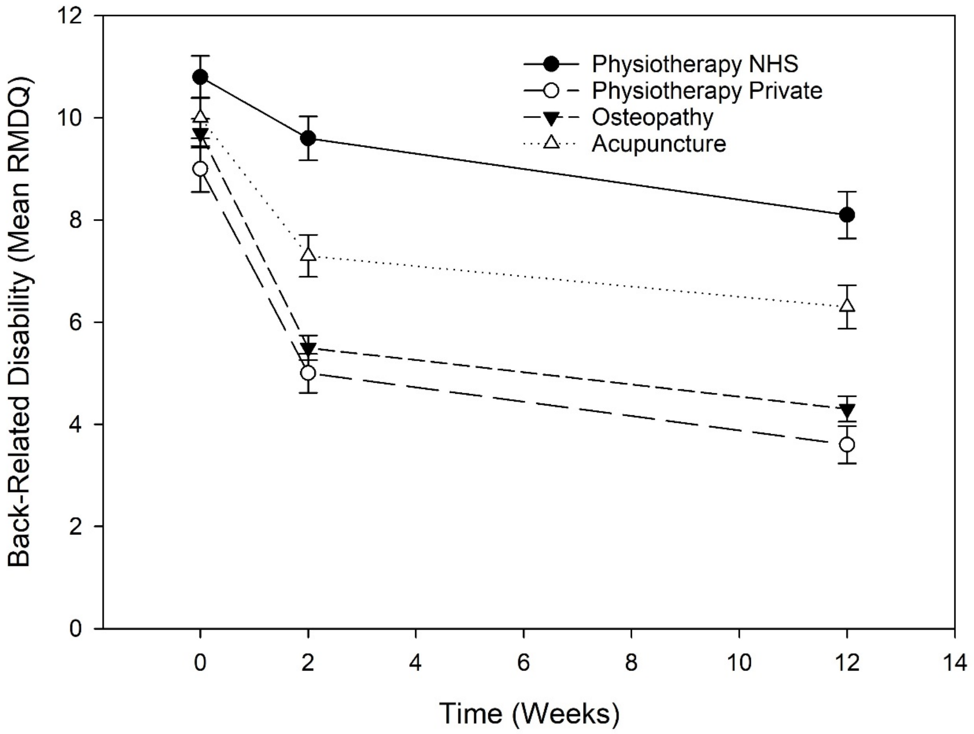

Figure 3. General Mediation Model

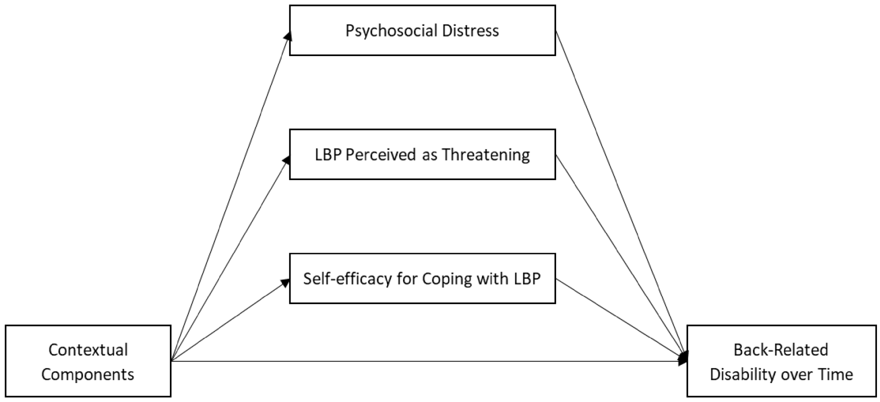

References

1. Roland M, Morris R. A study of the natural history of back pain. Part I: Development of a reliable and sensitive measure of disability in low-back pain. *Spine* 1983;8 (2):141-44.
2. Deyo RA, Battie M, Beurskens AJHM, et al. Outcome measures for low back pain research: A proposal for standardized use. *Spine* 1998;23(18):2003-13.
3. Busseri MA, Tyler JD. Interchangeability of the Working Alliance Inventory and Working Alliance Inventory, Short Form. *Psychological Assessment* 2003;15(2):193-97.
4. Tracey TJ, Kokotovic AM. Factor structure of the Working Alliance Inventory. *Psychological Assessment: A Journal of Consulting and Clinical Psychology* 1989;1(3):207-10.
5. Pincus T, Foster NE, Vogel S, et al. Attitudes to back pain amongst musculoskeletal practitioners: A comparison of professional groups and practice settings using the ABS-mp. *Manual Therapy* 2007;12(2):167-75.
6. Pincus T, Vogel S, Santos R, et al. The Attitudes to Back Pain Scale in Musculoskeletal Practitioners (ABS-mp): The Development and Testing of a New Questionnaire. *Clin J Pain* 2006;22(4):378-86.
7. Grogan S, Conner M, Norman P, et al. Validation of a questionnaire measuring patient satisfaction with general practitioner services. *Quality and Safety in Health Care* 2000;9(4):210-15.
8. Dima A, Lewith GT, Little P, et al. Patients' treatment beliefs in low back pain: development and validation of a questionnaire in primary care. *Pain* 2015;156(8):1489-500. doi: 10.1097/j.pain.0000000000000193 [published Online First: 2015/04/24]
9. Anderson KO, Dowds BN, Pelletz RE, et al. Development and initial validation of a scale to measure self-efficacy beliefs in patients with chronic pain. *Pain* 1995;63(1):77-83.
10. Broadbent E, Petrie KJ, Main J, et al. The Brief Illness Perception Questionnaire. *J Psychosom Res* 2006;60:631-37.
11. Hill JC, Dunn KM, Lewis M, et al. A primary care back pain screening tool: Identifying patient subgroups for initial treatment. *Arthritis Care Res* 2008;59(5):632-41.
