# Supplementary material for: Direct and mediated effects of treatment context on low back pain outcome: a prospective cohort study
Source: BMJ Open. 2021 May 18;11(5):e044831. doi: 10.1136/bmjopen-2020-044831 (PMC8130743; doi:10.1136/bmjopen-2020-044831)
Supplement: Author's manuscript [file bmjopen-2020-044831.draft_revisions.pdf]

BMJ Open

BMJ Open is committed to open peer review. As part of this commitment we make the peer review history of every article we publish publicly available.

When an article is published we post the peer reviewers' comments and the authors' responses online. We also post the versions of the paper that were used during peer review. These are the versions that the peer review comments apply to.

The versions of the paper that follow are the versions that were submitted during the peer review process. They are not the versions of record or the final published versions. They should not be cited or distributed as the published version of this manuscript.

BMJ Open is an open access journal and the full, final, typeset and author-corrected version of record of the manuscript is available on our site with no access controls, subscription charges or pay-per-view fees (http://bmjopen.bmj.com).

If you have any questions on BMJ Open's open peer review process please email info.bmjopen@bmj.com

BMJ Open

Direct and Mediated Effects of Treatment Context on Low Back Pain Outcome: A Prospective Cohort Study

Journal:	*BMJ Open*
Manuscript ID	bmjopen-2020-044831
Article Type:	Original research
Date Submitted by the Author:	15-Sep-2020
Complete List of Authors:	Bishop, Felicity; University of Southampton, Psychology<br>Al-Abbadey, Miznah; University of Portsmouth, Department of Psychology<br>Roberts, Lisa; University of Southampton, Health Sciences; University Hospital Southampton NHS Foundation Trust, Therapy Services<br>MacPherson, Hugh; University of York, Health Sciences<br>Stuart, Beth; University of Southampton, Primary Care and Population Sciences<br>Carnes, Dawn; Queen Mary University of London, Institute of Population Health Sciences<br>Fawkes, Carol; Queen Mary University of London, Institute of Population Health Sciences<br>Yardley, Lucy; University of Southampton, Psychology; University of Bristol, School of Health Sciences<br>Bradbury, Katherine; University of Southampton, Psychology
Keywords:	REHABILITATION MEDICINE, PRIMARY CARE, Back pain < ORTHOPAEDIC & TRAUMA SURGERY, COMPLEMENTARY MEDICINE

SCHOLARONE™
Manuscripts

I, the Submitting Author has the right to grant and does grant on behalf of all authors of the Work (as defined in the below author licence), an exclusive licence and/or a non-exclusive licence for contributions from authors who are: i) UK Crown employees; ii) where BMJ has agreed a CC-BY licence shall apply, and/or iii) in accordance with the terms applicable for US Federal Government officers or employees acting as part of their official duties; on a worldwide, perpetual, irrevocable, royalty-free basis to BMJ Publishing Group Ltd ("BMJ") its licensees and where the relevant Journal is co-owned by BMJ to the co-owners of the Journal, to publish the Work in this journal and any other BMJ products and to exploit all rights, as set out in our licence.

The Submitting Author accepts and understands that any supply made under these terms is made by BMJ to the Submitting Author unless you are acting as an employee on behalf of your employer or a postgraduate student of an affiliated institution which is paying any applicable article publishing charge ("APC") for Open Access articles. Where the Submitting Author wishes to make the Work available on an Open Access basis (and intends to pay the relevant APC), the terms of reuse of such Open Access shall be governed by a Creative Commons licence – details of these licences and which Creative Commons licence will apply to this Work are set out in our licence referred to above.

Other than as permitted in any relevant BMJ Author's Self Archiving Policies, I confirm this Work has not been accepted for publication elsewhere, is not being considered for publication elsewhere and does not duplicate material already published. I confirm all authors consent to publication of this Work and authorise the granting of this licence.

For peer review only

Direct and Mediated Effects of Treatment Context on Low Back Pain Outcome: A Prospective Cohort Study

Felicity L Bishop,[1*] Miznah Al-Abbadey, [1,2] Lisa Roberts, [3,4] Hugh MacPherson, [5] Beth Stuart, [6] Dawn Carnes, [7] Carol Fawkes, [7] Lucy Yardley, [1,8] Katherine Bradbury.[1]

[1] Department of Psychology, University of Southampton, Southampton, UK.
[2] Department of Psychology, University of Portsmouth, Portsmouth, UK.
[3] Health Sciences, University of Southampton, Southampton, UK.
[4] Therapy Services, University Hospital Southampton NHS Foundation Trust, Southampton, UK
[5] Health Sciences, University of York, York, UK.
[6] Primary Care and Population Sciences, University of Southampton, Southampton, UK.
[7] Institute of Population Health Sciences, Queen Mary University of London, London, UK.
[8] School of Psychological Science, University of Bristol, Bristol, UK

*Correspondence concerning this article should be addressed to Felicity L Bishop. Centre for Clinical and Community Applications of Health Psychology, Faculty of Environmental and Life Sciences, Building 44 Highfield Campus, University of Southampton, Southampton SO17 1BJ United Kingdom. Email: F.L.Bishop@southampton.ac.uk.

Word count, excluding title page, abstract, references, figures and tables: 4388

For peer review only

Abstract

Objectives: Contextual components of treatment previously associated with patient outcomes include the environment, therapeutic relationship, and expectancies. Questions remain about which components are most important, how they influence outcomes and comparative effects across treatment approaches. We aimed to identify significant and strong contextual predictors of patient outcomes, test for psychological mediators, and compare effects across three treatment approaches.

Design: Prospective cohort study with patient-reported and practitioner-reported questionnaire data (online or paper) collected at first consultation, 2-weeks, and 3-months.

Setting: Physiotherapy, osteopathy, and acupuncture clinics throughout the UK.

Participants: 166 practitioners (65 physiotherapists, 46 osteopaths, 55 acupuncturists) were recruited via their professional organisations. Practitioners recruited 960 adult patients seeking treatment for low back pain (LBP).

Primary and Secondary Outcomes: The primary outcome was back-related disability. Secondary outcomes were pain and well-being. Contextual components measured were: therapeutic alliance; patient satisfaction with appointment systems, access, facilities; patients' treatment beliefs including outcome expectancies; practitioners' attitudes to LBP; and practitioners' patient-specific outcome expectancies. The hypothesised mediators measured were: patient self-efficacy for pain management; patient perceptions of LBP; and psychosocial distress.

Results: After controlling for baseline and potential confounders, statistically significant predictors of reduced back-related disability were: all three dimensions of stronger therapeutic alliance (goal, task and bond); higher patient satisfaction with appointment systems; reduced patient-perceived treatment credibility; and increased practitioner-rated outcome expectancies. Therapeutic alliance over task (η_p^2=0.10 95% CI 0.07-0.14) and practitioner-rated outcome expectancies (η_p^2=0.08 95% CI 0.05-0.11) demonstrated the largest effect sizes. Patients' self-efficacy, LBP perceptions, and

For peer review only

psychosocial distress partially mediated these relationships. There were no interactions with treatment approach.

Conclusions: Enhancing contextual components in musculoskeletal healthcare could improve patient outcomes. Interventions should focus on helping practitioners and patients forge effective therapeutic alliances with strong affective bonds and agreement on treatment goals and how to achieve them.

Keywords: health knowledge, attitudes, practice; low back pain; placebo effects; therapeutic relationship; complementary therapies; context.

Article Summary

- The prospective but non-experimental design requires cautious interpretation regarding possible causal relationships between variables.
- 97% of the sample size was achieved (960 vs 986), with higher retention than anticipated (77% vs 70%) and the final sample at follow-up exceeded that required a priori (742 vs 690).
- The geographical spread across the UK and the large cohort size suggests the results may be generalizable beyond this sample.
- All variables were measured using self-report questionnaires, potentially introducing social desirability and/or shared-method bias; the former was partially mitigated by patients returning questionnaires directly to the researchers, not their clinicians; the latter was partially mitigated by the inclusion of patient and practitioner completed questionnaires and the separation in time of the measurement of predictors and outcomes.

For peer review only

INTRODUCTION

All healthcare interventions are delivered within a context that may significantly enhance or hinder an intervention's beneficial effects. Furthermore, contextual components, such as features of the environment or the patient's beliefs or "mind-set,"[1 2] may be shared across similar therapies. Therefore, identifying and strengthening beneficial contextual components could offer an efficient means of enhancing the effectiveness of multiple interventions. Low back pain (LBP) was chosen as the condition of interest as it is highly prevalent with considerable burden to individuals, communities, health services and the economy[3] and there is scope and need to improve patient outcomes across multiple non-pharmacological treatments. However, before embarking on intervention development and testing, it is vital to determine which contextual components should be targeted to produce the greatest likelihood of patient benefit.

Contextual components comprise the broad constellation of psychological, social, and environmental factors that act alongside and can interact with core components of interventions. Five domains have been proposed: patient-practitioner relationship; patients' beliefs; practitioners' beliefs; healthcare environment; and, for pharmacological interventions, incidental characteristics of treatment such as the colour of medications.[4] Particular components have been associated with improved patient satisfaction and/or clinical outcomes across multiple settings or specifically within non-pharmacological musculoskeletal care. Such components include: positive and empathetic patient-practitioner relationships and a strong patient-practitioner alliance[4-10] (patient-practitioner relationship); patients' outcome expectancies and perceived treatment credibility[9 11-16] (patient beliefs); musculoskeletal practitioners' beliefs about pain[17-22] and outcome expectancies[23] (practitioner beliefs); good organisational environments (e.g. collegiality)[24] the physical-sensory environment (e.g. music)[25] and the healthcare sector[26-28] (environment), although evidence of effects of healthcare sector on patient outcomes is mainly qualitative. Contextual components might impact patient outcomes via i) behavioural pathways (e.g. improved self-management,[29 30] but see also Haanstra et al.[31] ii) psychosocial pathways (e.g. reconceptualising pain as less threatening;[32]

For peer review only

[33 34] increased self-efficacy[35 36]), and/or iii) neurophysiological pathways.[37-41] The focus in this study was on psychosocial pathways; more details on how these were hypothesised to operate are provided in our published protocol.[42]

In summary, there is good evidence that contextual components can impact patient outcomes. However, most studies have focused on one or two components, meaning that direct comparisons have not been made to ascertain the relative contribution of different contextual components to patient health outcomes. Few studies have quantitatively tested potential pathways in the context of usual clinical practice. Most studies have focused on one treatment, meaning that direct comparisons have not been made to compare contextual components and their effects across treatment approaches. Therefore, the purpose of this study was to investigate the effects and mediating pathways of multiple contextual components on patient outcomes across three different treatment approaches in the private and public sectors. Physiotherapy, osteopathy, and acupuncture were selected as they are popular, safe, and relatively effective therapies for LBP[44-46] recommended by clinical guidelines at the conception of this study.[47 48]

The aims of the study were to (1) identify contextual components of treatment for LBP that predict patient outcomes, (2) test whether psychosocial factors mediate effects of contextual components on patient outcomes, and (3) compare components and their effects across physiotherapy, osteopathy, and acupuncture. In relation to aim (1), based on the literature it was hypothesised that patients would experience less back-related disability after treatment for LBP when contextual components are more positive (i.e., stronger therapeutic alliance; more pleasant/ accessible/ convenient/ supportive healthcare environment; positive patient-reported treatment beliefs and outcome expectancies; positive practitioner-reported outcome expectancies and practitioner having a biopsychosocial orientation to LBP). However, there were no strong grounds for a priori hypotheses concerning which component would be the strongest predictor of patient outcomes. In relation to aim (2), it was hypothesised that contextual components reduce patients' back-related disability by reducing the perceived threat of pain and/or increasing patients' self-

For peer review only

efficacy for coping with pain and/or reducing psychosocial distress. For aim (3), the effects of contextual components on patient outcomes were hypothesised to be larger in osteopathy and acupuncture – as examples of what have been classed complementary therapies - than in physiotherapy, which is usually considered a conventional therapy. This hypothesis was based on evidence that patients find the context of complementary therapies particularly valuable,[49] that the consultation process (alone, but in the context of a sham treatment) is effective in acupuncture for Irritable Bowel Syndrome[8] and that the rituals and modes of delivery of complementary therapies may enhance their effects.[50]

METHODS

Ethical Considerations

Ethical approval was obtained from NHS Health Research Authority NRES Committee East Midlands – Derby (REF: 14/EM/1113) on 22nd August 2014. Participants gave written informed consent; study documents conveyed the right to decline or withdraw without affecting medical care.

Design

In this prospective cohort study, practitioners and patients completed questionnaires at three time-points: baseline (after the first consultation for a new episode of LBP); during treatment (2-weeks post-baseline); and short-term outcome (3-months post-baseline). Contextual components were measured once each either at baseline or 2-weeks (specified in Measures), with time-points chosen to reduce ceiling effects, capture data accurately, and spread questionnaire burden. Outcomes, prognostic indicators and potential mediators were measured repeatedly at baseline, 2-weeks and 3-months, to permit tests of whether scores on contextual factors were associated with changes over time in prognostic indicators or mediators. Participants chose to complete hard copies (mailed, returned via Freepost) or electronic copies (emailed, completed online at isurvey.soton.ac.uk). Online and paper versions of the primary outcome are equivalent,

For peer review only

and used interchangeably, as patients value having this choice.[51] The full protocol was published.[42] See Supplemental Material Table 1 for deviations from the protocol.

Procedure

Between April 2015 and April 2017, physiotherapists, osteopaths, and acupuncturists working in the NHS and the private sector throughout the UK were recruited by advertisements and personal invitations, with support from professional societies. Inclusion criteria were: treat at least one patient with LBP on average per week, have at least three years' current experience in musculoskeletal work, and be registered (or eligible to register, for acupuncturists) with the appropriate professional body. To better capture current clinical practice, no restrictions were placed on type of intervention being delivered, meaning that osteopaths and physiotherapists were able to use acupuncture if and when they would usually do so.

Written consent was obtained from eligible practitioners, who gave potentially eligible patients a study invitation pack (containing invitation letter, information sheet, consent form, and baseline questionnaire). Patients were recruited between May 2015 and April 2017. On inviting an eligible patient into the study, practitioners rated their outcome expectancy for that patient on an anonymised form, labelled only with the same unique identifier as the corresponding patient invitation pack. Each outcome expectancy form was only analysed if the corresponding patient consent to participate was received by the researchers. Inclusion criteria were: at least 18 years old, seeking treatment from a participating practitioner at their first consultation for a new episode of LBP, and score at least 4/24 on the Roland-Morris Disability Questionnaire (RMDQ).[52] Exclusion criteria were: unable to complete questionnaires in English or Welsh, have serious underlying pathology (inflammatory arthritis, malignancy), or have practitioner-identified conditions that would prevent the sought treatment being applied. Arguably, LBP as managed in primary care should be conceptualised as typically having a chronic-episodic timeline with recurrent acute flare-ups against

For peer review only

a backdrop of temporary remission or less bothersome symptoms.[53 54] Broad inclusion criteria therefore best reflect the clinical situation.

Completed consent forms and questionnaires were returned directly to the researchers via prepaid envelopes. The researchers emailed or posted (participant's choice) the 2-week and 3-months questionnaires to participants. Techniques used to enhance retention were: non-response follow-ups (resending questionnaires and telephoning); monthly study e-newsletters to practitioners; small gifts (e.g. tea bag/pen) and monetary incentives (£5 voucher) for patients; personalised questionnaires; ink-signed cover letters; coloured ink; stamped return envelopes; first class post.[55]

Measures

This section describes how each construct was operationalised, explains the choice of questionnaires and the timing of each measure. Where other measures of constructs existed, questionnaires were chosen for their conceptual fit, psychometric properties, and conciseness. Supplemental Material Table 2 provides additional details including: example items, response scales, and Cronbach's alphas.

Outcomes

The primary outcome was self-reported back-related disability, measured using the 24-item Roland-Morris Disability Questionnaire (RMDQ).[52] Secondary outcomes (pain intensity, well-being, work and social role disability, satisfaction with care) were measured using recommended core single items.[56] All primary and secondary outcomes were measured at baseline, 2-weeks, and 3-months.

Contextual Components

Patient-Practitioner Relationship

For peer review only

EFFECTS OF TREATMENT CONTEXT

The patient-practitioner relationship was operationalised in this study as the therapeutic alliance. The therapeutic alliance is conceptually grounded in theory on how patient-practitioner interactions can elicit psychological and/or behavioural changes[57] both of which may be important in LBP. Therapeutic alliance was assessed using the 12-item patient-reported Working Alliance Inventory Short Form (WAI-SF), which measures three dimensions of working alliance – task collaboration, affective bond, and goal concordance - (four items each) with acceptable psychometric properties.[58] Task collaboration refers to the extent to which patients feel that the treatment approach their practitioner is taking is the right one for them. Affective bond refers to the extent to which patients feel an interpersonal connection with their practitioner including mutual respect and liking. And goal concordance refers to the extent to which patients feel they agree with their practitioner on the outcomes they are aiming to achieve through treatment. The patient-reported WAI-SF may have ceiling effects if used after one treatment but tends to remain stable after the second treatment;[6] it was therefore administered at 2-weeks.[60]

The Healthcare Environment

Practitioners' perceptions of the organisational environment were measured at baseline using two sub-scales from the psychometrically sound Attitudes to Back Pain Scale – Musculoskeletal Practitioners (ABS-mp): putting limits on sessions (4 items) and perceived connections within the health care system (3 items).[17 59] Patients' perceptions of the organisational and sensory-physical environment were assessed at 2-weeks using three sub-scales of the Patient Satisfaction Questionnaire (PSQ) designed to assess patient perceptions of the quality of primary healthcare in the UK.[60] The access sub-scale measures perceptions of interactions with reception staff (8 items); the appointments sub-scale measures perceived availability of convenient appointments (4 items); the facilities sub-scale measures perceptions of the physical environment of the clinic and waiting room (4 items).

For peer review only - http://bmjopen.bmj.com/site/about/guidelines.xhtml

EFFECTS OF TREATMENT CONTEXT

Patients' Beliefs

Four dimensions of treatment beliefs were measured using the brief LBP Treatment Beliefs Questionnaire: expected effectiveness, credibility, concerns, and individualised fit (4 items per subscale).[61] This questionnaire was explicitly designed for use in mixed cohorts of patients with LBP undergoing diverse treatments. It was completed at baseline as patients' expectancies regarding effectiveness should be measured early in treatment.[13]

Practitioners' Beliefs

A single-item numerical rating scale at baseline measured practitioners' outcome expectancies for each patient. Four sub-scales from the ABS-mp measured, at baseline, practitioners': willingness to engage with psychological issues (4 items); confidence and concern over clinical limitations (2 items); re-activation of work and activity (3 items); and belief in an underlying structural cause of pain (3 items).[17 59]

Hypothesised Mediators

Hypothesised mediators, i.e. constructs thought to be on the causal pathway between contextual components and patient outcomes, were measured at baseline, 2-weeks, and 3-months. The reliable and valid 9-item brief illness perceptions questionnaire (BIPQ) was used to measure the extent to which patients perceive their LBP as threatening.[62] Self-efficacy for coping with pain was assessed using the 5-item Chronic Pain Self-Efficacy for Pain Management sub-scale.[63] Psychosocial distress was assessed using the 5-item Psychosocial scale on the STarT Back questionnaire[64] which assesses multiple modifiable psychosocial prognostic factors.

Clinical and Socio-Demographic Characteristics

Patient and practitioner characteristics were assessed at baseline. Patient characteristics were: leg pain and shoulder/neck pain bothersomeness (measured using the single item on bothersomeness

For peer review only

as worded on the STarT Back tool[64] adapted from[65]); LBP duration; age; gender; work status; compensation status; co-morbidities; co-treatments; socio-economic status. To indicate socio-economic status, patients' postcodes were collected and used to assign the associated index of multiple deprivation (IMD) score to assign to each patient. The IMD ranks 32,844 small areas in England from most to least deprived; scores from 1 to 10 are then assigned to each area indicating the decile of deprivation, and these can be looked up by postcode. An IMD score of 1 indicates the area is among the most deprived 10% of areas.[66] Practitioner characteristics were: time since qualifying; experience in musculoskeletal care; age; gender.

Practitioners reported for each patient individually at three months which treatment modalities had been applied (reported in Supplemental Material Table 3).

Statistical Analysis

Power Calculation

The planned multilevel analysis required a final sample size of 690 patients to detect correlations between the predictors, hypothesised mediators, and the primary outcome with Pearson's R = 0.2 (and corresponding Beta regression coefficients of 0.235) with 95% power and p<0.0005 (2-tailed). This sample size was adjusted for clustering of patients within practitioners: the basic sample size was inflated by a factor of 1 +(k-1)*ICC, where K = 8 and ICC = 0.01.[67] (K = mean patients recruited per practitioner, ICC = intra-cluster correlation.) It was also intended to allow testing for interactions and possible treatment-specific effects. The target sample size required 86 practitioners to actively recruit patients (29 practitioners per therapy). To allow for 30% dropout,[15] we aimed to recruit 986 patients.

Data Entry

Paper questionnaires were entered online by researchers and checked for accuracy. All data were downloaded into MS Excel, cleaned, and exported into SPSS v22 and Stata for analysis.

For peer review only

Analysis

Data were explored using descriptive statistics and graphs. Cronbach's alpha was calculated to summarise the internal consistency of the measures at baseline. No imputation was undertaken for missing values.

To test the extent to which each contextual component predicted patient outcomes over time (Aim 1), multi-level linear mixed models were run with observations nested within patients over time and patients nested within practitioners. Separate models were run to test the effect of each individual contextual component on patient outcomes. These models excluded hypothesised mediators but did control for baseline back-related disability and other potential confounders specified in the protocol: leg pain and shoulder/neck pain bothersomeness; duration of LBP; age; gender; work status; compensation status; co-morbidities; co-treatments; socio-economic status (using deprivation index as indicated by post-code). The effect size was calculated for significant predictors using the partial eta-squared for multivariate regression, which can be interpreted as follows: 0.02 is a small effect, 0.13 moderate and 0.26 large (see http://imaging.mrc-cbu.cam.ac.uk/statswiki/FAQ/effectSize).

To test whether the effect of contextual components on patient outcomes was mediated by perceived threat of pain, self-efficacy for coping and/or psychosocial distress (Aim 2), the Baron and Kenny approach was used.[68] This involved (1) testing whether each hypothesized mediator was associated with the outcome (RMDQ), and then (2) controlling for those hypothesized mediators that were associated with the outcome in the analyses of those contextual components which had a statistically significant relationship with the outcome. This enabled a determination of whether the relationship between the contextual components and outcome remained significant or whether the relationship was largely explained by the presence of the mediators. Mediators were included in models as repeated measures.

For peer review only

To compare the effects of contextual components on patient outcomes across osteopathy, acupuncture, and physiotherapy (Aim 3), the presence of significant interactions between contextual components and treatment type was tested.

Patient Public Involvement

One volunteer, with personal experience of back pain and clinical research, acted as a patient advisor in this study. She attended team meetings and was involved in decision-making around study procedures and conduct including advising on the design of patient-facing study documents. She also wrote to participants to thank them for taking part. Involving members of the public in the management of research helps to improve researchers' awareness of participants' needs and perspectives and can enhance the design and conduct of studies.[69]

RESULTS

Participants

Overall, 166 practitioners and 960 patients participated. On average, each practitioner recruited 5.7 patients (SD=8.2). Figure 1 shows overall participant flow; this is split by treatment group in Supplemental Material Figure 1.

Table 1 shows patient characteristics at baseline, split by treatment group. Most patients were female, university-educated, resided in the 50% least deprived neighbourhoods in the country, had LBP for up to 3 months, did not have a significant comorbidity, and were using additional treatment(s). The group of patients accessing physiotherapy on the NHS differed from the other groups, they were less likely to hold postgraduate qualifications, more likely to be unemployed or on sick leave, more likely to have chronic LBP and more likely to report a compensation or other legal claim related to their LBP (N.B. absolute numbers of claims were low).

As a cohort, practitioners had been working for 20 years since qualifying (M=20.6, SD=10.1) and felt they were highly experienced at treating patients with LBP (M=4.3, SD=0.8). Compared to

For peer review only

the other groups, physiotherapists working in the NHS had been working for less years since qualifying (M=15.5, SD=7.2) and rated themselves as less experienced with LBP (M=3.8, SD=1.3). Supplemental Material Table 4 presents full practitioner characteristics by treatment group.

For peer review only

Table 1. Characteristics at Baseline of Patients with a New Episode of Low Back Pain, for the Whole Sample and for Subgroups of Patients Receiving Physiotherapy in the NHS, Physiotherapy in the Private Sector, Osteopathy, and Acupuncture, n (%)

	Whole sample	Physiotherapy NHS	Physiotherapy Private Sector	Osteopathy	Acupuncture
Practitioners					
Total	166 (100%)	36 (21.7%)	29 (17.5%)	46 (27.7%)	55 (33.1%)
Patients					
Total	960 (100%)	196 (20.4%)	165 (17.2%)	394 (41.0%)	205 (21.4%)
Age (years): M (SD)	52.3 (14.7)	52.4 (15.2)	55.4 (14.6)	57.3 (14.3)	56.0 (15.0)
Gender					
Female	605 (63.0%)	127 (64.8%)	103 (62.4%)	238 (60.4%)	137 (66.8%)
Male	338 (35.2%)	65 (33.2%)	61 (37.0%)	151 (38.3%)	61 (29.8%)
Missing data	17 (1.8%)	4 (2.0%)	1 (0.6%)	5 (1.3%)	7 (3.4%)
Education					
Up to secondary	39 (4.1%)	12 (6.1%)	1 (0.6%)	18 (4.6%)	8 (3.9%)
Finished secondary	306 (31.9%)	72 (36.73%)	39 (23.6%)	128 (32.5%)	67 (32.7%)
Finished 6th form	214 (22.3%)	51 (26.0%)	35 (21.2%)	94 (23.9%)	34 (16.6%)
Undergraduate	258 (26.9%)	40 (20.4%)	62 (37.6%)	97 (24.6%)	59 (28.8%)
Postgraduate	123 (12.8%)	13 (6.6%)	27 (16.4%)	51 (12.9%)	32 (15.6%)
Missing data	20 (2.1%)	8 (4.1%)	1 (0.6%)	6 (1.5%)	5 (2.4%)
Work status					
Employed	527 (54.9%)	116 (59.2%)	92 (55.8%)	211 (53.6%)	108 (52.7%)
Unemployed/sick leave	65 (6.8%)	22 (11.2%)	8 (4.8%)	22 (5.6%)	13 (6.3%)
Student/homemaker	60 (6.3%)	11 (5.6%)	14 (8.5%)	20 (5.1%)	15 (7.3%)
Retired	280 (29.2%)	43 (21.9%)	50 (30.3%)	125 (31.7%)	62 (30.2%)
Missing data	28 (2.9%)	4 (2.0%)	1 (0.6%)	16 (4.1%)	7 (3.4%)
Healthcare Sector					
Private Sector	742 (77.3%)	0	165 (100%)	389 (98.7%)	188 (91.7%)

For peer review only

EFFECTS OF TREATMENT CONTEXT

NHS	218 (22.7%)	196 (100%)	0	5 (1.3%)	17 (8.3%)
Duration of back pain					
Up to 1 week	127 (13.2%)	8 (4.1%)	37 (22.4%)	67 (17.0%)	15 (7.3%)
1 week – 1 month	257 (26.8%)	20 (10.2%)	50 (30.3%)	140 (35.5%)	47 (22.9%)
1 month -3 months	179 (18.6%)	41 (20.9%)	33 (20.0%)	77 (19.5%)	28 (13.7%)
3 months – 1 year	158 (16.5%)	57 (29.1%)	23 (13.9%)	48 (12.2%)	30 (14.6%)
1 year +	214 (22.3%)	62 (31.6%)	20 (12.1%)	55 (14.0%)	77 (37.6%)
Missing data	25 (2.6%)	8 (4.1%)	2 (1.2%)	7 (1.8%)	8 (3.9%)
Number of previous episodes					
None	210 (21.9%)	58 (29.6%)	33 (20.0%)	64 (16.2%)	55 (26.8%)
5 or fewer	317 (33.0%)	62 (31.6%)	60 (36.4%)	147 (37.3%)	48 (23.4%)
More than 5	349 (36.4%)	53 (27.0%)	62 (37.6%)	154 (39.1%)	80 (39.0%)
Missing data	84 (8.8%)	23 (11.7%)	10 (6.1%)	29 (7.4%)	22 (10.7%)
Comorbidity					
Longstanding illness or disability (besides LBP)	321 (33.4%)	71 (36.2%)	51 (30.9%)	119 (30.2%)	80 (39.0%)
Missing data	17 (1.8%)	7 (3.6%)	1 (0.6%)	8 (2.0%)	5 (2.4%)
Co-treatment					
Currently using other treatments	562 (58.5%)	123 (62.8%)	89 (53.9%)	215 (54.6%)	135 (65.9%)
Missing data	17 (1.8%)	10 (5.1%)	2 (1.2%)	12 (3.0%)	4 (2.0%)
Compensation					
Compensation or legal claim for LBP	27 (2.8%	13 (6.6%)	5 (3.0%)	6 (1.5%)	3 (1.5%)
Missing data	17 (1.8%)	4 (2.0%)	1 (0.6%)	6 (1.5%)	4 (2.0%)

Note. NHS = National Health Service. LBP = Low Back Pain.

For peer review only

Outcomes over Time

On average, patients improved from baseline to 2-weeks and from 2-weeks to 3-months on all primary and secondary outcome measures, except for overall satisfaction with healthcare which remained similarly high at all measurement points. Figure 2 displays the change in back-related disability over time; outcomes by treatment group and secondary outcomes are provided in Supplemental Material Figure 2 and Table 4 respectively.

Contextual Predictors of Back-Related Disability over Time

Table 2 shows the results of models assessing the relationship between each contextual component and back-related disability over time. After controlling for baseline disability and potential confounders, statistically significant predictors of reduced back-related disability over time were: stronger therapeutic alliance on all three dimensions; higher patient satisfaction with appointment systems; reduced perceived credibility of chosen treatment; and increased practitioner-rated outcome expectancies.

Therapeutic alliance concerning task and practitioner-rated outcome expectancies demonstrated the largest effect sizes. For each unit increase in patients' rating of the therapeutic alliance (task), patient-rated back-related disability over time decreased by 2.33 points (95% CI -2.89 to -1.77) on the RMDQ. For each unit increase in practitioners' patient-specific outcome expectancies, patient-rated back-related disability over time decreased by 1.29 points (95% CI -1.66 to -0.92) on the RMDQ.

The minimum clinically-important difference for primary care patients with LBP has been calculated as a standard 2-3 points[70] or a 30% reduction in RMDQ score from baseline.[71] Therefore, a one unit increase in patients' rating of the therapeutic alliance (task) and a two unit increase in practitioners' outcome expectancies would be associated with a clinically-important reduction in back-related disability for patients scoring 7 or less on the RMDQ at baseline. For patients scoring 8 to 15 points on the RMDQ at baseline, a two-unit increase in patients' rating of the therapeutic

For peer review only

alliance (task) and a three-unit increase in practitioners' outcome expectancies may be needed to make a clinically-important difference.

Mediation Analysis to Examine Mechanisms

All the mediators were significantly associated with the outcome (for descriptive statistics see Supplemental Material Table 6). For each unit increase in self-efficacy for coping with pain, RMDQ improved by 0.07 (95% CI -0.08, -0.05). As psychosocial distress increased by one unit, RMDQ increased by 1.43 (95% CI 1.09, 1.77). And as overall perception of back pain as threatening increased by one unit, RMDQ score increased by 0.23 (95% CI 0.21, 0.25). After controlling for these three variables in the longitudinal multilevel models, the measures of therapeutic alliance, satisfaction with appointment systems, and reduced perceived credibility of treatment were no longer statistically significant predictors of RMDQ (Table 2). This suggests that their effects on disability are indeed mediated by increased self-efficacy for coping with pain, reduced perception of back pain as threatening, and reduction in psychosocial distress.

Practitioners' outcome expectancies at baseline showed reduced but still statistically significant effects on RMDQ changes over time, suggesting that their effects are only partially mediated. In all cases the mediators remained associated with the outcome in the multivariable models, except for self-efficacy, which was not statistically significant in the model with practitioners' outcome expectancies. This suggests that the effect of practitioners' outcome expectancies at baseline on changes in RMDQ over time was partially mediated by changes in perception of back pain as threatening and psychosocial distress. For a visual summary of the general mediation model tested, see Supplemental Material Figure 3.

For peer review only

EFFECTS OF TREATMENT CONTEXT

Table 2. Longitudinal Models Assessing Contextual Predictors of Back-Related Disability over Time in Patients Receiving Physiotherapy, Osteopathy, or Acupuncture for a New Episode of Low Back Pain

Contextual component	Mean (SD)	Change in RMDQ for one-unit change in component: Unadjusted for confounders	Adjusted for confounders[a]	Adjusted for mediators[b]	Effect size partial eta-squared (95% CI)[c]
Therapeutic alliance: goal	4.2 (0.7)	-1.14 (-1.69, -0.60)	-0.94 (-1.54, -0.35)	0.36 (-0.09, 0.82)	0.02 (0.004, 0.04)
Therapeutic alliance: task	4.0 (0.6)	-2.78 (-3.33, -2.23)	-2.33 (-2.89, -1.77)	0.13 (-0.34, 0.61)	0.10 (0.07, 0.14)
Therapeutic alliance: bond	4.1 (0.9)	-1.14 (-1.56, -0.32)	-0.86 (-1.30, -0.43)	-0.10 (-0.43, 0.23)	0.03 (0.01, 0.05)
Environment: Limits on Treatment	11.5 (2.9)	-0.08 (-0.25, 0.09)	-0.03 (-0.23, 0.16)		
Environment: Connections within the Healthcare System	11.3 (2.3)	0.01 (-0.23, 0.25)	-0.05 (-0.27, 0.17)		
Environment: Satisfaction with appointment systems	1.9 (0.7)	1.31 (0.81, 1.82)	0.67 (0.13, 1.20)	-0.23 (-0.65, 0.14)	0.02 (0.01, 0.04)
Environment: Satisfaction with access	2.5 (0.6)	1.01 (0.38, 1.63)	0.60 (-0.04, 1.26)		
Environment: Satisfaction with facilities	2.2 (0.8)	0.52 (0.04, 1.01)	0.34 (-0.18, 0.86)		
Patient's beliefs: Perceived credibility of treatment	3.0 (0.3)	1.34 (0.19, 2.50)	1.51 (0.35, 2.67)	0.58 (-0.28, 1.44)	0.01 (0.001, 0.03)
Patient's beliefs: Expectancies for treatment effectiveness	2.8 (0.3)	0.17 (-0.99, 1.34)	0.12 (-1.05, 1.29)		
Patient's beliefs: Concerns about treatment	2.9 (0.3)	-0.36 (-1.45, 0.73)	-0.26 (-1.37, 0.86)		
Patient's beliefs: Individualised fit of treatment	2.9 (0.3)	0.14 (-1.07, 1.34)	0.59 (-0.65, 1.83)		
Practitioner's beliefs: Psychological	17.8 (3.6)	0.13 (-0.06, 0.32)	0.03 (-0.15, 0.20)		
Practitioner's beliefs: Confidence	8.2 (2.0)	0.03 (-0.27, 0.32)	0.10 (-0.19, 0.40)		
Practitioner's beliefs: Reactivation	15.3 (3.0)	-0.06 (-0.24, 0.13)	0.08 (-0.10, 0.25)		
Practitioner's beliefs: Biomedical	12.2 (3.6)	-0.06 (-0.22, 0.10)	-0.07 (-0.25, 0.11)		
Practitioner's beliefs: Outcome expectancies	5.7 (1.1)	-1.77 (-2.12, -1.42)	-1.29 (-1.66, -0.92)	-0.42 (-0.70, -0.13)	0.08 (0.05, 0.11)

Note. Table reports the results of separate models run to test the effect of each individual contextual factor on patient outcomes, specifically back-related disability. RMDQ = Roland Morris Disability Questionnaire. SD = Standard Deviation. [a] Adjusted for baseline back-related disability and: leg pain and shoulder/neck pain bothersomeness; duration of LBP; age; gender; work status; compensation status; co-morbidities; co-treatments; socio-economic status.

[b] Adjusted for mediation by perceived threat of pain, self-efficacy for coping and psychosocial distress. [b] Effect size only reported for statistically significant contextual predictors of back-related disability over time.

For peer review only

Comparisons across Treatments

The longitudinal models revealed no significant interactions between contextual factors and treatment type. This indicates that the effects of contextual components on back-related disability do not differ significantly between osteopathy, acupuncture, and physiotherapy.

DISCUSSION

This large prospective cohort study investigated the effects and mediating pathways of multiple contextual components on patient outcomes across three different therapies in the private and public sectors. As hypothesised, components representing four non-pharmacological contextual domains were significantly associated with back-related disability over time in a large cohort of patients with LBP seeking osteopathy, acupuncture, and physiotherapy. The patient-practitioner therapeutic alliance and practitioners' expectancies of how individual patients will respond to treatment were the strongest contextual components, showing the largest effects on patient outcomes. These components reduced disability at least partly by improving patients' self-efficacy for coping and reducing psychosocial distress and the perceived threat of LBP. Other contextual components emerged as statistically significant predictors of improved outcome over time: the healthcare environment, specifically greater satisfaction with appointments; and (contrary to hypothesised direction of association) patients' beliefs, specifically reduced perceived credibility of one's chosen treatment. The relationship between contextual components and patient outcomes did not differ across osteopathy, acupuncture, and physiotherapy.

The current study extends understanding of the mechanisms through which contextual factors may impact patient health outcomes. Earlier studies have investigated pathways amongst smaller numbers of contextual variables and suggested, for example, that self-efficacy mediates the impact on osteoarthritis pain of practitioners communicating positive expectancies,[35] affective aspects of the therapeutic relationship may shape patient expectancies in chronic pain,[75] and expectancies might impact pain via reduced catastrophising in chronic back pain.[33] The current

For peer review only

results further suggest contextual factors reduce back-related disability in part by enhancing patient self-efficacy for coping with pain, reducing the perceived threat of pain, and reducing psychosocial distress. This is broadly consistent with the fear avoidance model[34] and social cognitive theory.[36] It also builds on work in adjacent areas such as communication[41] and starts to map potential evidence-based psychological pathways through which contextual factors might impact patient outcomes.

The prospective but non-experimental design requires cautious interpretation regarding possible causal relationships between variables: the results provide evidence of predictive relationships between variables over time, which are consistent with, but insufficient to establish causality. While 97% of the baseline sample size was achieved (960 vs 986), retention was higher than anticipated (77% vs 70%) and the final sample at follow-up exceeded that required a priori (108%, n=742 vs 690). The geographical spread across the UK and the large cohort size suggests the results may be generalizable beyond this sample. All variables were measured using self-report questionnaires, potentially introducing social desirability and/or shared-method bias; the former was partially mitigated by patients returning questionnaires directly to the researchers, not their clinicians; the latter was partially mitigated by the inclusion of patient and practitioner completed questionnaires and the separation in time of the measurement of predictors and outcomes. Some of the ABS-MP subscales had low internal consistency in this sample (Cronbach's alphas <.6) suggesting the results of those analyses may be unreliable. Empathy and perceived competence, potentially important contextual components associated with the therapeutic relationship,[76] were not directly captured by the measure of therapeutic alliance.

The current findings highlight the relative importance of the therapeutic alliance as a contextual predictor of back-related disability over time, compared to other contextual components. This supports a growing emphasis on patient-practitioner communication and relationships in musculoskeletal healthcare interactions. Previous studies and reviews have also suggested the importance of the therapeutic relationship for positive patient outcomes[7 10] but have not considered the extensive selection of other contextual variables measured here. A recent systematic review in

For peer review only

physiotherapy concluded there was a lack of evidence of a strong relationship between therapeutic alliance and reduced pain, but the studies reviewed were all much smaller than the current cohort (n=12-182) and may have been underpowered.[72] The only other contextual factor to approach a moderate effect size on patient outcomes was the practitioners' outcome expectancies at baseline, which predicted outcomes even after controlling for baseline clinical and socio-demographic variables. This factor has been somewhat overlooked in the literature but was also found to predict outcomes in a large acupuncture study.[23] The reasons for this association likely involve practitioners making global clinical judgements that incorporate factors not measured in the current study. The lack of association between patients' expectancies and subsequent outcomes was unexpected given the balance of previous findings linking patient expectancies to outcomes,[9 73] but not unprecedented: expectancies are more strongly and consistently associated with experimental acute and procedural pain than chronic pain.[11] This null finding may be related to timing, as expectancies may change over time[74] and patients can be reluctant to specify expectancies about effectiveness early in treatment, while concepts such as hope may have greater resonance in clinical settings.[43] The lack of associations between patient outcomes and practitioner perceptions of the healthcare environment, attitudes and orientation to LBP was also unexpected, but may be an artefact of the low reliability of the ABS-MP subscales.

Future research, using qualitative methods, could explore how practitioners form positive outcome expectancies and how this might be harnessed in practice. The possible causal nature of and mechanisms by which practitioner outcome expectancies impact patient outcomes also require further research.

Having a strong therapeutic alliance and positive practitioner expectancies of outcome were the strongest contextual predictors of reduced disability in a large cohort of patients with LBP receiving physiotherapy, osteopathy and acupuncture. These operate in part by altering known psychological prognostic factors: self-efficacy, perceived pain threat, and psychosocial distress. Enhancing these contextual components in practice could improve patient health outcomes from

For peer review only

EFFECTS OF TREATMENT CONTEXT

LBP. While a strong therapeutic alliance concerning the ultimate goals of treatment and a strong affective bond are associated with improved patient health outcomes, it is the strength of alliance concerning how to achieve the goals (i.e., the tasks of treatment) that has the largest effect on improved outcomes. The lack of between-treatment differences in significant predictors suggests the potential for shared learning across therapies to improve contextual components. Therefore, it is advisable for clinicians to focus on strengthening their alliance building related to how the patient should reach an agreed-upon goal within the context of a strong affective bond. Evidence-based, engaging, training could be developed to support skills in therapeutic alliance building.

For peer review only

FUNDING STATEMENT

This work was supported by Arthritis Research UK Special Strategic Award grant number 20552.

AUTHOR CONTRIBUTIONS

We would like to acknowledge the vital contribution that three, now deceased, colleagues made to the design and management of this research: Dr Borislav Dimitrov (University of Southampton), Dr Jan Leach (University of Brighton), and Professor George Lewith (University of Southampton).

FLB led the study conception and design and drafted the manuscript. FLB, KB, HM, LY, George Lewith, Borislav Dimitrov, and Jan Leach conceptualised the study, and with LR drafted the protocol and the associated grant application. FLB, KB, HM, LY, MA, and LR drafted the manuscript. MA was the research fellow on the study, responsible for data collection under the supervision of FLB and KB and in collaboration with all authors. BS undertook the statistical analysis. All authors revised the manuscript critically for important intellectual content and gave final approval of the version to be published.

We would like to thank all the practitioners and patients who took part in this study. The Clinical Research Network, British Acupuncture Council, Chartered Society of Physiotherapy, General Osteopathic Council, and the Institute of Osteopathy assisted with recruitment. Research and administrative assistance was provided by: Laura Wilde, Sue Eardley, Maddy Greville-Harris, J Matthew Harvey, Daniel Scanlan, Ricky Wong, Tasnia Priti, Daniela Baramova, Helen Loader, Nicole Collaco, Yasmin Hoque, and Senada Merkulaj. Our Patient Public Involvement collaborator was Jo Foster.

COMPETING INTERESTS STATEMENT

FLB received an honorarium and travel expenses for presenting the preliminary results of this research at the Acupuncture Research Resource Centre research symposium and has also received speaker's fees and travel expenses from the Acupuncture Association of Chartered Physiotherapists. The authors declare that they have no other competing interests.

DATA SHARING STATEMENT

Deidentified participant data may be requested from the corresponding author, Dr Felicity Bishop (https://orcid.org/0000-0002-8737-6662). Access may only be permitted for the purposes of checking the reported analyses and an analysis plan must be submitted with any request for access to the data. Due to ethical considerations, reuse for any other purpose is not permitted.

For peer review only

REFERENCES

1. Crum AJ, Corbin WR, Brownell KD, et al. Mind over milkshakes: mindsets, not just nutrients, determine ghrelin response. *Health Psychol* 2011;30(4):424-9; discussion 30-1. doi: 10.1037/a0023467 [published Online First: 2011/05/18]
2. Crum AJ, Zuckerman B. Changing mindsets to enhance treatment effectiveness. *JAMA* 2017;317(20):2063-64. doi: 10.1001/jama.2017.4545
3. GBD 2015 Disease and Injury Incidence and Prevalence Collaborators. Global, regional, and national incidence, prevalence, and years lived with disability for 310 diseases and injuries, 1990-2015: a systematic analysis for the Global Burden of Disease Study 2015. *Lancet* 2016;388(10053):1545-602. doi: 10.1016/S0140-6736(16)31678-6
4. Di Blasi Z, Harkness E, Ernst E, et al. Influence of context effects on health outcomes: a systematic review. *Lancet* 2001;357:757-62.
5. Roter DL. Which facets of communication have strong effects on outcome - a meta analysis. In: Stewart MA, Roter DL, eds. Communicating with Medical Patients. Newbury Park, CA: Sage Publications 1989.
6. Martin DJ, Garske JP, Davis MK. Relation of the therapeutic alliance with outcome and other variables: A meta-analytic review. *J Consult Clin Psychol* 2000;68(3):438-50.
7. Hall AM, Ferreira PH, Maher CG, et al. The Influence of the Therapist-Patient Relationship on Treatment Outcome in Physical Rehabilitation: A Systematic Review. *Phys Ther* 2010;90(8):1099-110.
8. Kaptchuk TJ, Kelley JM, Conboy LA, et al. Components of placebo effect: randomised controlled trial in patients with irritable bowel syndrome. *Br Med J* 2008;336:999-1003.
9. Howick J, Moscrop A, Mebius A, et al. Effects of empathic and positive communication in healthcare consultations: a systematic review and meta-analysis. *J R Soc Med* 2018;111(7):240-52. doi: 10.1177/0141076818769477 [published Online First: 2018/04/20]
10. Draper-Rodi J, Vogel S, Bishop A. Identification of prognostic factors and assessment methods on the evaluation of non-specific low back pain in a biopsychosocial environment: A scoping review. *International Journal of Osteopathic Medicine* 2018;30:25-34. doi: 10.1016/j.ijosm.2018.07.001
11. Peerdeman KJ, van Laarhoven AIM, Keij SM, et al. Relieving patients' pain with expectation interventions: a meta-analysis. *Pain* 2016;157(6):1179-91. doi: 10.1097/j.pain.0000000000000540
12. Devilly GJ, Borkovec TD. Psychometric properties of the credibility/expectancy questionnaire. *J Behav Ther Exp Psychiatry* 2000;31:73-86.
13. Crow R, Gage H, Hampson S, et al. The role of expectancies in the placebo effect and their use in the delivery of health care: a systematic review. *Health Technol Assess* 1999;3(3)
14. Colagiuri B, Smith CA. A systematic review of the effect of expectancy on treatment responses to acupuncture. *Evid Based Complement Alternat Med* 2012:doi:10.1155/2012/857804.
15. Bishop FL, Yardley L, Prescott P, et al. Psychological covariates of longitudinal changes in back-related disability in patients undergoing acupuncture. *Clin J Pain* 2015;31(3):254-64. doi: 10.1097/ajp.0000000000000108 [published Online First: 2014/06/06]
16. Kong J, Wang Z, Leiser J, et al. Enhancing treatment of osteoarthritis knee pain by boosting expectancy: A functional neuroimaging study. *Neuroimage-Clinical* 2018;18:325-34. doi: 10.1016/j.nicl.2018.01.021
17. Pincus T, Foster NE, Vogel S, et al. Attitudes to back pain amongst musculoskeletal practitioners: A comparison of professional groups and practice settings using the ABS-mp. *Manual Therapy* 2007;12(2):167-75.
18. Bishop A, Foster NE, Thomas E, et al. How does the self-reported clinical management of patients with low back pain relate to the attitudes and beliefs of health care practitioners? A survey of UK general practitioners and physiotherapists. *Pain* 2008;135:187-95.

For peer review only

19. Houben RMA, Ostelo RWJG, Vlaeyen JWS, et al. Health care providers' orientations towards common low back pain predict perceived harmfulness of physical activities and recommendations regarding return to normal activity. *European Journal of Pain* 2005;9(2):173-83. doi: 10.1016/j.ejpain.2004.05.002
20. Simmonds MJ, Derghazarian T, Vlaeyen JWS. Physiotherapists' Knowledge, Attitudes, and Intolerance of Uncertainty Influence Decision Making in Low Back Pain. *The Clinical Journal of Pain* 2012;28(6):467-74. doi: 10.1097/AJP.0b013e31825bfe65
21. Thomson OP, Petty NJ, Moore AP. Osteopaths' professional views, identities and conceptions – A qualitative grounded theory study. *International Journal of Osteopathic Medicine* 2014;17(3):146-59. doi: https://doi.org/10.1016/j.ijosm.2013.12.002
22. Hughes JG, Goldbart J, Fairhurst E, et al. Exploring acupuncturists' perceptions of treating patients with rheumatoid arthritis. *Complement Ther Med* 2007;15(2):101-08.
23. Witt CM, Martins F, Willich SN, et al. Can I help you? Physicians' expectations as predictor for treatment outcome. *European Journal of Pain* 2012;16(10):1455-66.
24. Linda HA, Walter S, Koen VdH, et al. Patient safety, satisfaction, and quality of hospital care: cross sectional surveys of nurses and patients in 12 countries in Europe and the United States. *Br Med J* 2012;344
25. Drahota A, Ward D, Mackenzie H, et al. Sensory environment on health-related outcomes of hospital patients (Review). *Cochrane Database of Systematic Reviews* 2012;3:Art. No.: CD005315. DOI: 10.1002/14651858.CD005315.pub2.
26. Bishop FL, Barlow F, Coghlan B, et al. Patients as healthcare consumers in the public and private sectors: a qualitative study of acupuncture in the UK. *BMC Health Serv Res* 2011;11(129):http://www.biomedcentral.com/1472-6963/11/129.
27. Bishop FL, Amos N, Yu H, et al. Health-care sector and complementary medicine: practitioners' experiences of delivering acupuncture in the public and private sectors. *Prim Health Care Res Dev* 2012;13(03):269-78.
28. Bradbury KJ, Bishop FL, Yardley L, et al. Patients' appraisals of public and private healthcare: A qualitative study of physiotherapy and osteopathy. *J Health Psychol* 2013;18(10):1307-18.
29. Evans M, Paterson C, Wye L, et al. Lifestyle and self-care advice within traditional acupuncture consultations: A qualitative observational study nested in a co-operative inquiry. *J Altern Complement Med* 2011;17(6):519-29. doi: 10.1089/acm.2010.0749.
30. Paterson C, Evans M, Bertschlinger R, et al. Communication about self-care in traditional acupuncture consultations: The co-construction of individualised support and advice. *Patient Educ Couns* 2012;89(3):467-75.
31. Haanstra TM, Kamper SJ, Williams CM, et al. Does adherence to treatment mediate the relationship between patients' treatment outcome expectancies and the outcomes of pain intensity and recovery from acute low back pain? *Pain* 2015;156(8):1530-36. doi: 10.1097/j.pain.0000000000000198
32. Agarwal V. Re-conceptualizing pain through patient-centred care in the complementary and alternative medicine therapeutic relationship. *J Adv Nurs* 2018;74(10):2406-15. doi: 10.1111/jan.13734
33. Carriere JS, Martel MO, Meints SM, et al. What do you expect? Catastrophizing mediates associations between expectancies and pain-facilitatory processes. *European journal of pain (London, England)* 2018 doi: 10.1002/ejp.1348
34. Vlaeyen JW, Linton SJ. Fear-avoidance model of chronic musculoskeletal pain: 12 years on. *Pain* 2012;153(6):1144-7. doi: 10.1016/j.pain.2011.12.009 [published Online First: 2012/02/11]
35. Lo GH-W, Balasubramanyam AS, Barbo A, et al. Link Between Positive Clinician-Conveyed Expectations of Treatment Effect and Pain Reduction in Knee Osteoarthritis, Mediated by Patient Self-Efficacy. *Arthritis Care Res* 2016;68(7):952-57. doi: 10.1002/acr.22775
36. Bandura A. Self-efficacy: toward a unifying theory of behavioral change. *Psychol Rev* 1977;84(2):191-215. [published Online First: 1977/03/01]

For peer review only

37. Carlino E, Benedetti F. Different contexts, different pains, different experiences. *Neuroscience* 2016;338:19-26. doi: https://doi.org/10.1016/j.neuroscience.2016.01.053
38. Lee I-S, Wallraven C, Kong J, et al. When pain is not only pain: Inserting needles into the body evokes distinct reward-related brain responses in the context of a treatment. *Physiol Behav* 2015;140:148-55. doi: https://doi.org/10.1016/j.physbeh.2014.12.030
39. Fields HL. How expectations influence pain. *Pain* 2018;159(9):S3-S10. doi: 10.1097/j.pain.0000000000001272
40. Neumann M, Bensing J, Mercer S, et al. Analyzing the "nature" and "specific effectiveness" of clinical empathy: A theoretical overview and contribution towards a theory-based research agenda. *Patient Educ Couns* 2009;74(3):339-46.
41. Street, Jr., Makoul G, Arora NK, et al. How does communication heal? Pathways linking clinician-patient communication to health outcomes. *Patient Educ Couns* 2009;74(3):295-301.
42. Bradbury KJ, Al-Abbadey M, Carnes D, et al. Non-specific mechanisms in orthodox and CAM management of low back pain (MOCAM): theoretical framework and protocol for a prospective cohort study. *BMJ Open* 2016;6(5):e012209. doi: 10.1136/bmjopen-2016-012209
43. Bishop FL, Lewith GT. Patients' preconceptions of acupuncture: a qualitative study exploring the decisions patients make when seeking acupuncture. *BMC Complement Altern Med* 2013;13:102. doi: DOI: 10.1186/1472-6882-13-102
44. Vickers AJ, Vertosick EA, Lewith G, et al. Acupuncture for Chronic Pain: Update of an Individual Patient Data Meta-Analysis. *J Pain* 2018;19(5):455-74. doi: 10.1016/j.jpain.2017.11.005
45. Breivik H, Collett B, Ventafridda V, et al. Survey of chronic pain in Europe: Prevalence, impact on daily life, and treatment. *European Journal of Pain* 2006;10(4):287-333.
46. Rubinstein SM, de Zoete A, van Middelkoop M, et al. Benefits and harms of spinal manipulative therapy for the treatment of chronic low back pain: systematic review and meta-analysis of randomised controlled trials. *BMJ* 2019;364:l689. doi: 10.1136/bmj.l689 [published Online First: 2019/03/15]
47. Oliveira CB, Maher CG, Pinto RZ, et al. Clinical practice guidelines for the management of non-specific low back pain in primary care: an updated overview. *Eur Spine J* 2018;27(11):2791-803. doi: 10.1007/s00586-018-5673-2
48. Qaseem A, Wilt TJ, McLean RM, et al. Noninvasive Treatments for Acute, Subacute, and Chronic Low Back Pain: A Clinical Practice Guideline From the American College of Physicians. *Ann Intern Med* 2017;166(7):514-30. doi: 10.7326/m16-2367
49. Foley H, Steel A. Patient perceptions of clinical care in complementary medicine: A systematic review of the consultation experience. *Patient Educ Couns* 2017;100(2):212-23. doi: 10.1016/j.pec.2016.09.015 [published Online First: 2016/10/04]
50. Kaptchuk TJ, Stason WB, Davis RB, et al. Sham device v inert pill: randomised controlled trial of two placebo treatments. *Br Med J* 2006;332(7538):391-94.
51. Bishop FL, Lewis G, Harris S, et al. A within-subjects trial to test the equivalence of online and paper outcome measures: the Roland Morris disability questionnaire. *BMC Musculoskelet Disord* 2010;11:113. doi: 10.1186/1471-2474-11-113 [published Online First: 2010/06/10]
52. Roland M, Morris R. A study of the natural history of back pain. Part I: Development of a reliable and sensitive measure of disability in low-back pain. *Spine* 1983;8 (2):141-44.
53. Croft PR, Macfarlane GJ, Papageorgiou AC, et al. Outcome of low back pain in general practice: a prospective study. *Br Med J* 1998;316:1356.
54. Hestbaek L, Leboeuf-Yde C, Engberg M, et al. The course of low back pain in a general population. results from a 5-year prospective study. *J Manipulative Physiol Ther* 2003;26(4):213-19.
55. Robinson KA, Dinglas VD, Sukrithan V, et al. Updated systematic review identifies substantial number of retention strategies: using more strategies retains more study participants. *J Clin*

For peer review only

Epidemiol 2015;68(12):1481-7. doi: 10.1016/j.jclinepi.2015.04.013 [published Online First: 2015/07/19]

56. Deyo RA, Battie M, Beurskens AJHM, et al. Outcome measures for low back pain research: A proposal for standardized use. *Spine* 1998;23(18):2003-13.
57. Horvath AO, Greenberg LS. Development and validation of the Working Alliance Inventory. *Journal of Counseling Psychology* 1989;36(2):223-33.
58. Busseri MA, Tyler JD. Interchangeability of the Working Alliance Inventory and Working Alliance Inventory, Short Form. *Psychological Assessment* 2003;15(2):193-97.
59. Pincus T, Vogel S, Santos R, et al. The Attitudes to Back Pain Scale in Musculoskeletal Practitioners (ABS-mp): The Development and Testing of a New Questionnaire. *Clin J Pain* 2006;22(4):378-86.
60. Grogan S, Conner M, Norman P, et al. Validation of a questionnaire measuring patient satisfaction with general practitioner services. *Quality and Safety in Health Care* 2000;9(4):210-15.
61. Dima A, Lewith GT, Little P, et al. Patients' treatment beliefs in low back pain: development and validation of a questionnaire in primary care. *Pain* 2015;156(8):1489-500. doi: 10.1097/j.pain.0000000000000193 [published Online First: 2015/04/24]
62. Broadbent E, Petrie KJ, Main J, et al. The Brief Illness Perception Questionnaire. *J Psychosom Res* 2006;60:631-37.
63. Anderson KO, Dowds BN, Pelletz RE, et al. Development and initial validation of a scale to measure self-efficacy beliefs in patients with chronic pain. *Pain* 1995;63(1):77-83.
64. Hill JC, Dunn KM, Lewis M, et al. A primary care back pain screening tool: Identifying patient subgroups for initial treatment. *Arthritis Care Res* 2008;59(5):632-41.
65. Dunn KM, Croft PR. Classification of Low Back Pain in Primary Care: Using "Bothersomeness" to Identify the Most Severe Cases. *Spine* 2005;30(16):1887-92.
66. Department for Communities and Local Government. The English Index of Multiple Deprivation (IMD) 2015–Guidance. Gov.UK, 2015.
67. Farrin A, Russell I, Torgerson D, et al. Differential recruitment in a cluster randomized trial in primary care: the experience of the UK back pain, exercise, active management and manipulation (UK BEAM) feasibility study. *Clin Trials* 2005;2(2):119-24. [published Online First: 2005/11/11]
68. Baron RM, Kenny DA. The Moderator Mediator Variable Distinction in Social Psychological-Research - Conceptual, Strategic, and Statistical Considerations. *J Pers Soc Psychol* 1986;51(6):1173-82.
69. Brett J, Staniszewska S, Mockford C, et al. Mapping the impact of patient and public involvement on health and social care research: a systematic review. *Health Expect* 2014;17(5):637-50. doi: 10.1111/j.1369-7625.2012.00795.x
70. Roland M, Fairbank J. The Roland Morris Disability Questionnaire and the Oswestry Disability Questionnaire. *Spine* 2000;25:3115-24.
71. Jordan K, Dunn KM, Lewis M, et al. A minimal clinically important difference was derived for the Roland-Morris Disability Questionnaire for low back pain. *J Clin Epidemiol* 2006;59(1):45-52. doi: https://doi.org/10.1016/j.jclinepi.2005.03.018
72. Taccolini Manzoni AC, Bastos de Oliveira NT, Nunes Cabral CM, et al. The role of the therapeutic alliance on pain relief in musculoskeletal rehabilitation: A systematic review. *Physiotherapy Theory and Practice* 2018;34(12):901-15. doi: 10.1080/09593985.2018.1431343
73. Peerdeman KJ, van Laarhoven AM, Peters ML, et al. An Integrative Review of the Influence of Expectancies on Pain. *Frontiers in Psychology* 2016;7 doi: 10.3389/fpsyg.2016.01270
74. Eaves ER, Sherman KJ, Ritenbaugh C, et al. A qualitative study of changes in expectations over time among patients with chronic low back pain seeking four CAM therapies. *BMC Complement Altern Med* 2015;15 doi: 10.1186/s12906-015-0531-9

For peer review only

75. Wiering B, de Boer D, Krol M, et al. Entertaining accurate treatment expectations while suffering from chronic pain: an exploration of treatment expectations and the relationship with patient- provider communication. *BMC Health Serv Res* 2018;18 doi: 10.1186/s12913-018-3497-8
76. Howe LC, Goyer JP, Crum AJ. Harnessing the placebo effect: Exploring the influence of physician characteristics on placebo response. *Health Psychol* 2017;36(11):1074-82. doi: 10.1037/hea0000499 [published Online First: 2017/03/09]

For peer review only

Figure Captions

Figure 1. Modified Consort Diagram. Showing Participant Flow, Recruitment and Drop-Out.

Figure 2. Back-Related Disability over Time. Mean scores and standard errors are plotted.

For peer review only

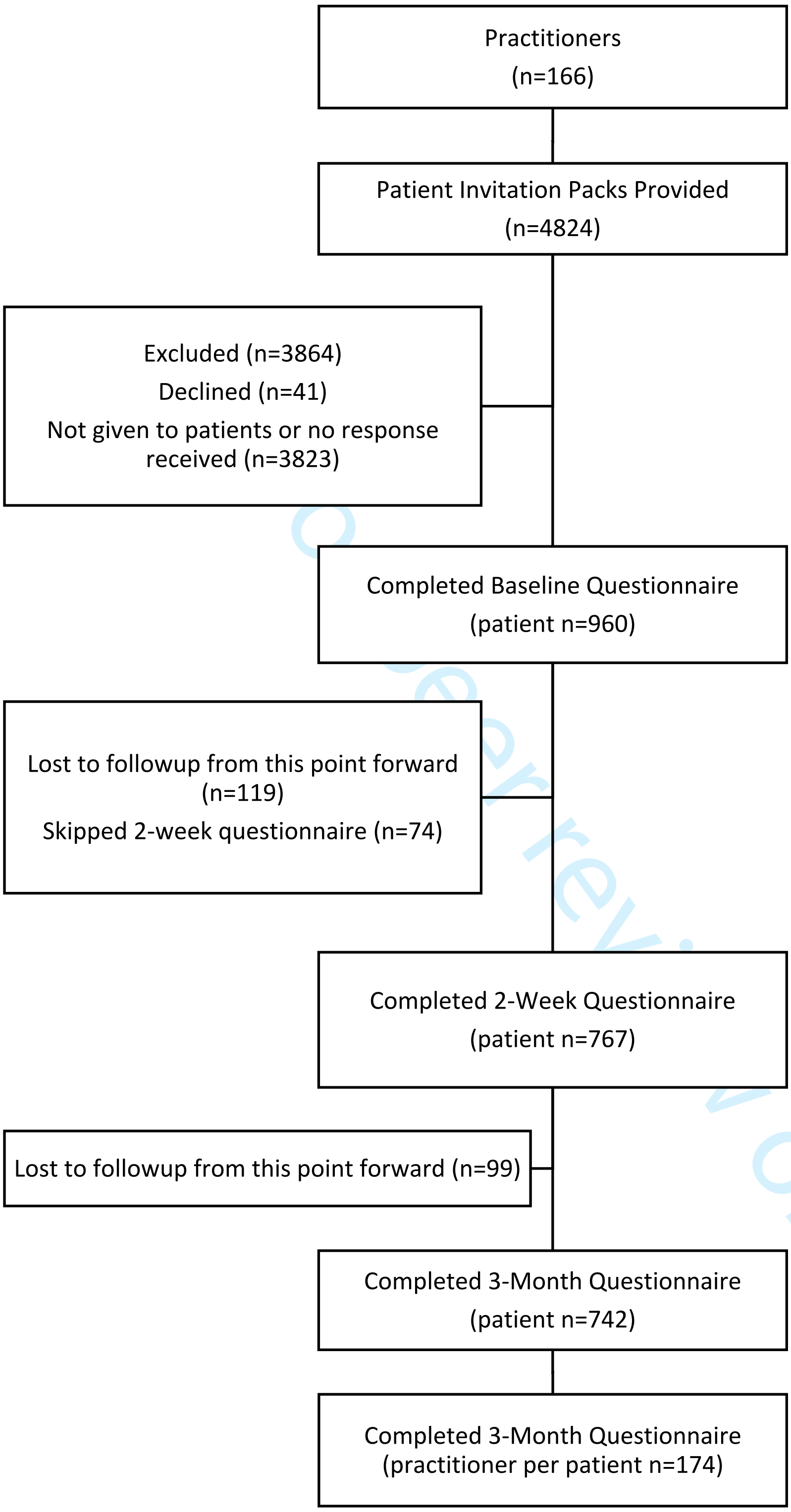

For peer review only

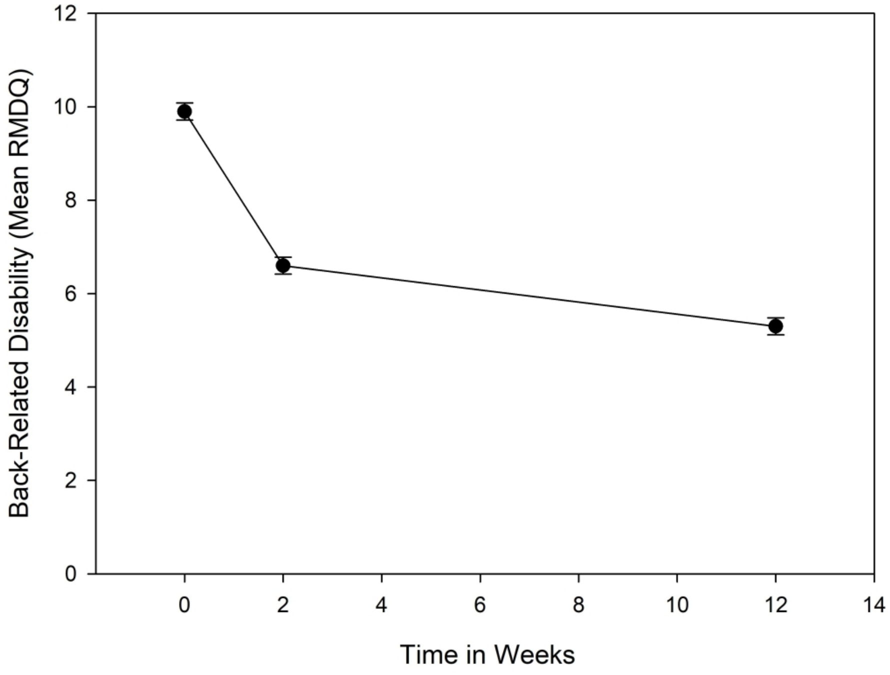

Figure 2. Back-Related Disability over Time. Mean scores and standard errors are plotted.

1003x761mm (96 x 96 DPI)

Direct and Mediated Effects of Treatment Context on Back Pain

Supplemental Material

Table 1: Deviations from Published Protocol

#	Deviation	Rationale
1	Unable to recruit equivalent numbers of practitioners and patients from the NHS and the private sector and therefore did not test the hypothesis that differences in contextual effects between acupuncture, osteopathy, and physiotherapy would be more pronounced in the NHS than the private sector.	Around the time recruitment began there was a marked decline in NHS commissioning of osteopathy and acupuncture for low back pain. This made it very difficult to recruit these groups.
2	Revised sample size calculation.	Necessary to reflect changes in sample as per deviation #1.
3	No test of whether effects of contextual components on back-related disability are mediated by increased patient adherence to theory-specific lifestyle advice (in addition to pain beliefs and self-efficacy).	Extensive missing patient self-report data on adherence and the variety of lifestyle advice precluded a test of this hypothesis.
4	Practitioner-reported data on caseload, waiting list, and appointment duration not collected.	Items were erroneously omitted from the questionnaire.

For peer review only

Table 2: Study Measures with Example Items, Response Scales, and Interpretation

Construct	Measure (subscale)	Example item	Response scale	Possible range	Cronbach's alpha**	High score indicates
Primary Outcomes						
Disability	RMDQ[1]	I change position frequently to try to get my back comfortable	Yes/No	0-24	-	Greater back-related disability
Secondary Outcomes						
Social Role Disability	Core single item[2]	During the past 4 weeks, how many days did back pain or leg pain (sciatica) keep you from going to work or college?[2]	Free text – number of days in past 4 weeks	0-28	-	Greater social role disability
Functional impairment	Core single item[2]	During the past week, how much did pain interfere with your normal work (including both work outside the home and housework)?[2]	5-point verbal	1-5	-	Greater functional impairment
Pain	Core single item[2]	Please rate your pain by indicating the number that best describes your pain on average in the last 24 hours.[2]	11-point numerical	0-10	-	Greater average pain over last 24 hours
Wellbeing	Core single item[2]	If you had to spend the rest of your life with the symptoms you have right now, how would you feel about it?	5-point verbal	1-5	-	Greater wellbeing
Satisfaction	Core single item[2]	Over the course of treatment for your back pain or leg pain (sciatica) so far, how satisfied are you with your overall health care?	5-point verbal	1-5	-	Greater satisfaction with treatment
Contextual Components						
Therapeutic alliance: task	WAI-SF[3 4] (Task)	I believe the way we are working with my problem is	5-point verbal	1-5	0.67	Greater patient-practitioner agreement

For peer review only

		correct				on task (i.e. how to achieve goals)
Therapeutic alliance: goal	WAI-SF[3 4] (Goal)	___ and I are working towards mutually agreed upon goals	5-point verbal	1-5	0.74	Greater patient-practitioner agreement on goals
Therapeutic alliance: bond	WAI-SF[3 4] (Bond)	I believe ___ likes me.	5-point verbal	1-5	0.86	Greater patient-practitioner interpersonal affective bond
Environment: Limits on Treatment*	ABS-mp[5 6] (Limitations on sessions)	If I keep seeing my patients on and off I can prevent relapse	7-point agree/disagree	4-28	0.46	Disinclined to put a limit on number of sessions or refer elsewhere
Environment: Connections within the Healthcare System*	ABS-mp[5 6] (Perceived connections within the health care system)	When referring patients I am confident they will receive good treatment	7-point agree/disagree	3-21	0.49	Feels well-connected to health care system resources
Environment: Satisfaction with appointment systems	PSQ[7] (Appointments)	Getting an appointment at a convenient time is easy	5-point agree/disagree	1-5	0.81	Less satisfied with appointment systems
Environment: Satisfaction with access	PSQ[7] (Access)	It is easy to get advice over the telephone	5-point agree/disagree	1-5	0.80	Less satisfied with access
Environment: Satisfaction with facilities	PSQ[7] (Facilities)	The waiting room is uncomfortable	5-point agree/disagree	1-5	0.75	Less satisfied with facilities
Patient's beliefs: Expectancies for treatment effectiveness	LBP treatment beliefs questionnaire[8] (Effectiveness)	[therapy] can work well for people with back pain	5-point agree/disagree	1-5	0.82	Stronger beliefs that therapy will be effective
Patient's beliefs: Perceived credibility of treatment	LBP treatment beliefs questionnaire [8] (Credibility)	Having [therapy] for back pain makes a lot of sense	5-point agree/disagree	1-5	0.85	Stronger beliefs that therapy is credible

For peer review only

Patient's beliefs: Concerns about treatment	LBP treatment beliefs questionnaire [8] (Concerns)	I feel that [therapy] would not harm me	5-point agree/disagree	1-5	0.83	Fewer concerns about therapy
Patient's beliefs: Individualised fit of treatment	LBP treatment beliefs questionnaire [8] (individual fit)	I am confident [therapy] would be a suitable treatment for my back pain	5-point agree/disagree	1-5	0.87	Stronger beliefs that therapy is good fit for oneself
Practitioner's beliefs: psychological*	ABS-mp [5 6] (Psychological)	I often find myself providing psychological support to patients	7-point agree/disagree	4-28	0.65	More willing to engage with patients' psychological issues
Practitioner's beliefs: confidence *	ABS-mp[5 6] (Confidence and concern)	I am concerned about the quality of treatment my referred patients receive	7-point agree/disagree	2-14	0.18	More confident and less concerned about one's own and others' clinical limitations
Practitioner's beliefs: reactivation*	ABS-mp[5 6] (Reactivation)	Return to normal daily activities is the most important consequence of treatment	7-point agree/disagree	3-21	0.57	Greater tendency to focus on re-activation of work and activity
Practitioner's beliefs: biomedical*	ABS-mp[5 6] (Biomedical)	If you look hard enough you can find a structural reason for most patients' back pain	7-point agree/disagree	3-21	0.57	Greater tendency to focus on biomedical model of back pain (also includes rest and vigilance)
Practitioner's beliefs: Outcome Expectancies*	Single item per patient	Thinking about this patient, how effective do you expect your course of treatment to be?	7-point numerical	1-7	-	Expects treatment to be extremely effective for this patient
Hypothesised Mediators						
Self-efficacy for pain management	Self-efficacy for pain management[9]	How certain are you that you can decrease your pain quite a bit?	10-point numerical	10-100	0.78	Higher self-efficacy for pain management
Perception of LBP as threatening	Brief IPQ[10]	How much does your back pain affect your life?	11-point numerical	0-80	0.73	LBP perceived as more threatening
Psychosocial distress	StartBack[11]	I feel that my back pain is terrible and it's never going to get any better	2-point agree/disagree	0-5	0.45	Greater psychosocial distress

For peer review only

* These questionnaires completed by practitioners. All others completed by patients.
** Cronbach's alpha in this sample
RMDQ, Roland Morris Disability Questionnaire. WAI-SF, Working Alliance Inventory – Short Form. ABS-mp, Attitudes to Back Pain Scale – Musculoskeletal Practitioners. PSQ, Patient Satisfaction Questionnaire. LBP, Low Back Pain. IPQ, Illness Perceptions Questionnaire.

For peer review only

Table 3. Treatment Characteristics by Treatment Group, n (%)

	Physiotherapy NHS (n=196)	Physiotherapy Private (n=165)	Osteopathy (n=394)	Acupuncture (n=205)
Number of weeks treated: M(SD)	6.9 (5.3)	6.1 (9.8)	4.3 (4.6)	8.6 (10.9)
Number of appointments attended: M (SD)	3.9 (2.7)	4.0 (3.2)	3.2 (2.3)	5.1 (4.2)
Treatment Modalities (>1 permitted)				
Manual therapy	61 (31.1%)	116 (70.3%)	248 (62.9%)	50 (24.4%)
Electro/thermal therapy	7 (3.6%)	30 (18.2%)	45 (11.4%)	59 (28.8%)
Acupuncture	21 (10.7%)	18 (10.9%)	46 (11.7%)	123 (60.0%)
Exercise	139 (70.9%)	117 (70.9%)	128 (32.5%)	16 (7.8%)
Lifestyle advice	127 (64.8%)	112 (67.9%)	205 (52.0%)	56 (27.3%)
Missing data	45 (23.0%)	44 (26.7%)	93 (23.6%)	80 (39.0%)

NHS, National Health Service. M, Mean. SD, Standard Deviation.

Note. The average duration of treatment was longest for patients seeing acupuncturists and shortest for patients seeing osteopaths. There was considerable missing data on the specific treatment modalities used for each patient (particularly for the acupuncture group). Among those patients for whom treatment modalities were reported, manual therapy techniques were most commonly used with patients consulting osteopaths and physiotherapists in the private sector; electro/thermal and acupuncture techniques were most likely to be applied by acupuncturists but were used for a minority of patients in the other groups; and patients consulting physiotherapists were most likely to undertake specific exercises or be given lifestyle advice. Categories were defined as follows: manual therapy (specifically, one or more of: mobilisation, articulation, grade V manipulation, high-velocity low-amplitude technique, soft tissue techniques, and East Asian techniques such as acupressure, tuina, gua sha, cupping); electro-therapy therapy (transcutaneous electrical nerve stimulation, ultrasound, electro-acupuncture, application of heat, application of cold, moxibustion); acupuncture (western acupuncture such as trigger point needling and segmental needling, traditionally-based acupuncture such as Traditional Chinese Medicine, five elements, stems and branches, Japanese needling); exercise (individual, group, hydrotherapy); lifestyle advice (therapy-specific and non-therapy-specific advice on diet, specific exercises, physical activity, rest, and relaxation).

For peer review only

Table 4. Practitioner Characteristics at Baseline by Treatment Group

	Whole sample (n=166)	Physiotherapy NHS (n=36)	Physiotherapy Private (n=29)	Osteopathy (n=46)	Acupuncture (n=55)
Years since qualifying	20.6 (10.1)	15.5 (7.2)	25.3 (11.0)	24.0 (10.9)	18.5 (8.6)
Self-rated experience treating LBP*	4.3 (0.8)	3.8 (1.3)	4.5 (0.9)	4.5 (0.7)	4.2 (0.7)
Age	49.7 (10.8)	39.2 (8.3)	50.8 (8.3)	55.0 (10.6)	53.3 (14.6)

* Rated on a 1-5 scale where 1 = not at all experienced; 5 = very experienced.

NHS, National Health Service. LBP, Low Back Pain.

For peer review only

Table 5. Secondary Outcomes over Time.

Secondary Outcome	Time	Mean (SD)
Social Role Disability	Baseline	2.0 (5.4)
Social Role Disability	2-weeks	N/A
Social Role Disability	3-months	0.9 (4.2)
Functional impairment	Baseline	3.2 (1.1)
Functional impairment	2-weeks	2.5 (1.1)
Functional impairment	3-months	2.2 (1.1)
Pain	Baseline	5.0 (2.2)
Pain	2-weeks	3.5 (2.4)
Pain	3-months	3.4 (2.5)
Wellbeing	Baseline	1.6 (0.9)
Wellbeing	2-weeks	2.3 (1.3)
Wellbeing	3-months	2.8 (1.4)
Satisfaction	Baseline	4.1 (1.0)
Satisfaction	2-weeks	4.1 (1.0)
Satisfaction	3-months	4.1 (1.1)

For peer review only

Table 6. Mean (Standard Deviation) Scores on Mediators over Time by Treatment Group

	Time	Whole sample	Physiotherapy NHS	Physiotherapy Private	Osteopathy	Acupuncture
Self-efficacy for coping with pain	Baseline	68.5 (19.7)	60.6 (19.2)	74.8 (23.0)	70.0 (17.9)	68.2 (18.3)
Self-efficacy for coping with pain	2-weeks	65.9 (25.3)	56.2 (24.0)	71.4 (26.6)	66.8 (26.0)	68.2 (21.9)
Self-efficacy for coping with pain	3-months	69.1 (24.1)	59.0 (24.3)	75.3 (24.6)	71.4 (23.7)	68.8 (21.7)
Perception of LBP as threatening	Baseline	37.2 (9.9)	41.0 (9.5)	34.3 (9.4)	35.4 (9.7)	39.8 (9.4)
Perception of LBP as threatening	2-weeks	32.7 (13.2)	38.9 (11.8)	27.9 (13.4)	30.7 (12.9)	35.2 (12.4)
Perception of LBP as threatening	3-months	32.1 (14.5)	36.8 (13.9)	26.8 (14.0)	30.0 (13.9)	35.5 (14.5)
Psychosocial complexity of LBP	Baseline	0.60 (0.7)	0.6 (0.6)	0.6 (0.6)	0.6 (0.7)	0.6 (0.7)
Psychosocial complexity of LBP	2-weeks	1.1 (1.8)	1.3 (1.9)	0.8 (1.6)	1.1 (1.9)	1.0 (1.7)
Psychosocial complexity of LBP	3-months	1.1 (1.9)	1.4 (1.9)	1.0 (1.9)	1.0 (1.9)	1.3 (1.9)

For peer review only

Figure 1. Modified Consort Diagram. Showing Participant Flow, Recruitment and Drop-Out by Therapy Group.

For peer review only

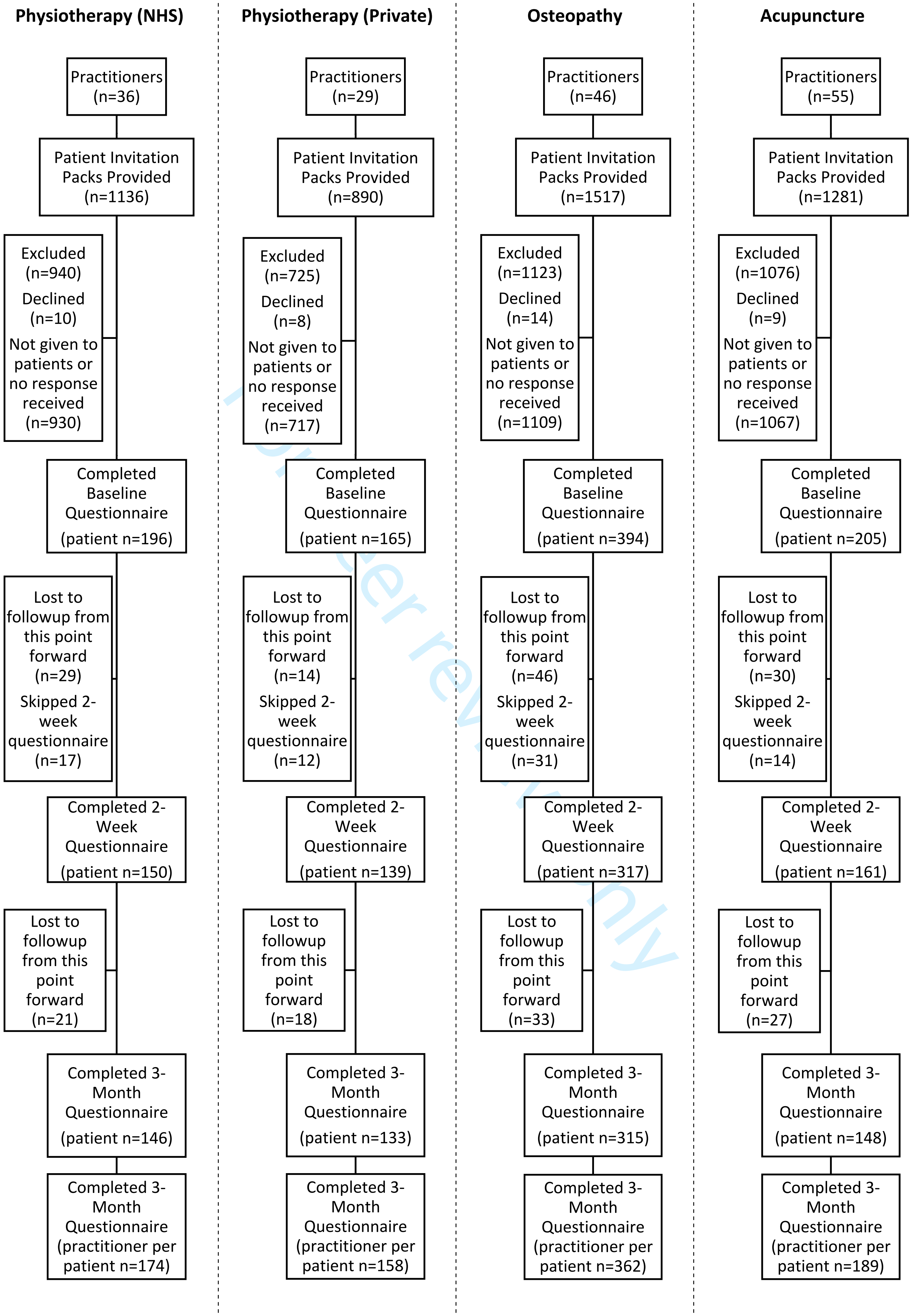
Physiotherapy (NHS)
Practitioners (n=36)
Patient Invitation Packs Provided (n=1136)
Excluded (n=940) Declined (n=10) Not given to patients or no response received (n=930)
Completed Baseline Questionnaire (patient n=196)
Lost to followup from this point forward (n=29) Skipped 2-week questionnaire (n=17)
Completed 2-Week Questionnaire (patient n=150)
Lost to followup from this point forward (n=21)
Completed 3-Month Questionnaire (patient n=146)
Completed 3-Month Questionnaire (practitioner per patient n=174)
Physiotherapy (Private)
Practitioners (n=29)
Patient Invitation Packs Provided (n=890)
Excluded (n=725) Declined (n=8) Not given to patients or no response received (n=717)
Completed Baseline Questionnaire (patient n=165)
Lost to followup from this point forward (n=14) Skipped 2-week questionnaire (n=12)
Completed 2-Week Questionnaire (patient n=139)
Lost to followup from this point forward (n=18)
Completed 3-Month Questionnaire (patient n=133)
Completed 3-Month Questionnaire (practitioner per patient n=158)
Osteopathy
Practitioners (n=46)
Patient Invitation Packs Provided (n=1517)
Excluded (n=1123) Declined (n=14) Not given to patients or no response received (n=1109)
Completed Baseline Questionnaire (patient n=394)
Lost to followup from this point forward (n=46) Skipped 2-week questionnaire (n=31)
Completed 2-Week Questionnaire (patient n=317)
Lost to followup from this point forward (n=33)
Completed 3-Month Questionnaire (patient n=315)
Completed 3-Month Questionnaire (practitioner per patient n=362)
Acupuncture
Practitioners (n=55)
Patient Invitation Packs Provided (n=1281)
Excluded (n=1076) Declined (n=9) Not given to patients or no response received (n=1067)
Completed Baseline Questionnaire (patient n=205)
Lost to followup from this point forward (n=30) Skipped 2-week questionnaire (n=14)
Completed 2-Week Questionnaire (patient n=161)
Lost to followup from this point forward (n=27)
Completed 3-Month Questionnaire (patient n=148)
Completed 3-Month Questionnaire (practitioner per patient n=189)

Figure 2. Primary Outcomes over Time by Therapy Group

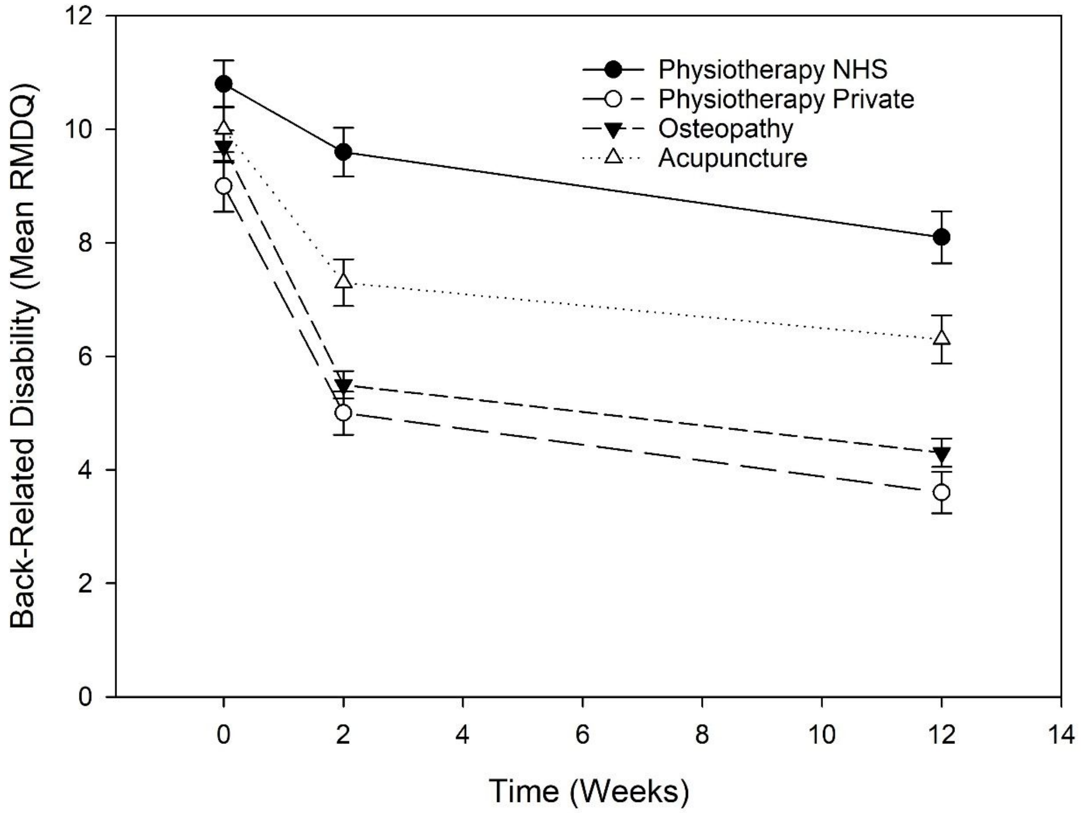

w only

Figure 3. General Mediation Model

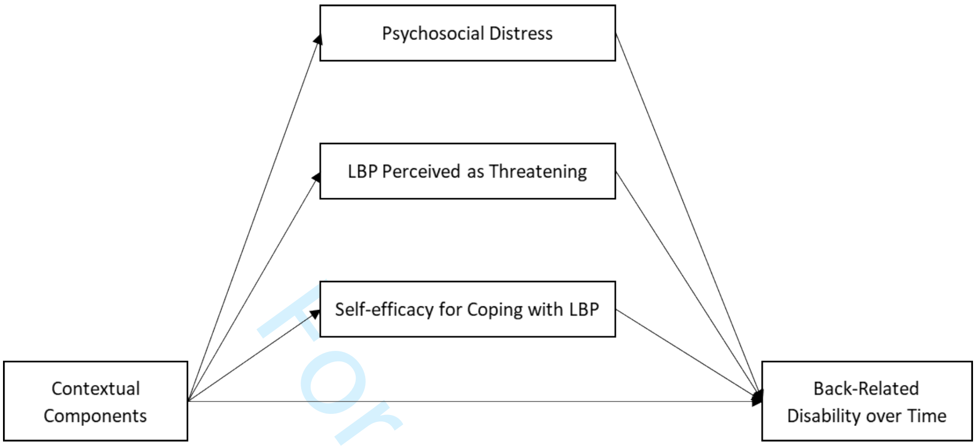

For peer review only

References

1. Roland M, Morris R. A study of the natural history of back pain. Part I: Development of a reliable and sensitive measure of disability in low-back pain. *Spine* 1983;8 (2):141-44.
2. Deyo RA, Battie M, Beurskens AJHM, et al. Outcome measures for low back pain research: A proposal for standardized use. *Spine* 1998;23(18):2003-13.
3. Busseri MA, Tyler JD. Interchangeability of the Working Alliance Inventory and Working Alliance Inventory, Short Form. *Psychological Assessment* 2003;15(2):193-97.
4. Tracey TJ, Kokotovic AM. Factor structure of the Working Alliance Inventory. *Psychological Assessment: A Journal of Consulting and Clinical Psychology* 1989;1(3):207-10.
5. Pincus T, Foster NE, Vogel S, et al. Attitudes to back pain amongst musculoskeletal practitioners: A comparison of professional groups and practice settings using the ABS-mp. *Manual Therapy* 2007;12(2):167-75.
6. Pincus T, Vogel S, Santos R, et al. The Attitudes to Back Pain Scale in Musculoskeletal Practitioners (ABS-mp): The Development and Testing of a New Questionnaire. *Clin J Pain* 2006;22(4):378-86.
7. Grogan S, Conner M, Norman P, et al. Validation of a questionnaire measuring patient satisfaction with general practitioner services. *Quality and Safety in Health Care* 2000;9(4):210-15.
8. Dima A, Lewith GT, Little P, et al. Patients' treatment beliefs in low back pain: development and validation of a questionnaire in primary care. *Pain* 2015;156(8):1489-500. doi: 10.1097/j.pain.0000000000000193 [published Online First: 2015/04/24]
9. Anderson KO, Dowds BN, Pelletz RE, et al. Development and initial validation of a scale to measure self-efficacy beliefs in patients with chronic pain. *Pain* 1995;63(1):77-83.
10. Broadbent E, Petrie KJ, Main J, et al. The Brief Illness Perception Questionnaire. *J Psychosom Res* 2006;60:631-37.
11. Hill JC, Dunn KM, Lewis M, et al. A primary care back pain screening tool: Identifying patient subgroups for initial treatment. *Arthritis Care Res* 2008;59(5):632-41.

For peer review only

STROBE Statement—Checklist of items that should be included in reports of ***cohort studies***

	Item No	Recommendation	
**Title and abstract**	1	(*a*) Indicate the study's design with a commonly used term in the title or the abstract	✓
		(*b*) Provide in the abstract an informative and balanced summary of what was done and what was found	✓
**Introduction**			
Background/rationale	2	Explain the scientific background and rationale for the investigation being reported	✓
Objectives	3	State specific objectives, including any prespecified hypotheses	✓
**Methods**			
Study design	4	Present key elements of study design early in the paper	✓
Setting	5	Describe the setting, locations, and relevant dates, including periods of recruitment, exposure, follow-up, and data collection	✓
Participants	6	(*a*) Give the eligibility criteria, and the sources and methods of selection of participants. Describe methods of follow-up	✓
		(*b*) For matched studies, give matching criteria and number of exposed and unexposed	N/A
Variables	7	Clearly define all outcomes, exposures, predictors, potential confounders, and effect modifiers. Give diagnostic criteria, if applicable	✓
Data sources/ measurement	8*	For each variable of interest, give sources of data and details of methods of assessment (measurement). Describe comparability of assessment methods if there is more than one group	✓
Bias	9	Describe any efforts to address potential sources of bias	✓ (in discussion)
Study size	10	Explain how the study size was arrived at	✓
Quantitative variables	11	Explain how quantitative variables were handled in the analyses. If applicable, describe which groupings were chosen and why	✓
Statistical methods	12	(*a*) Describe all statistical methods, including those used to control for confounding	✓
		(*b*) Describe any methods used to examine subgroups and interactions	✓
		(*c*) Explain how missing data were addressed	✓
		(*d*) If applicable, explain how loss to follow-up was addressed	✓
		(*e*) Describe any sensitivity analyses	N/A
**Results**			
Participants	13*	(a) Report numbers of individuals at each stage of study—eg numbers potentially eligible, examined for eligibility, confirmed eligible, included in the study, completing follow-up, and analysed	✓
		(b) Give reasons for non-participation at each stage	✓
		(c) Consider use of a flow diagram	✓
Descriptive data	14*	(a) Give characteristics of study participants (eg demographic, clinical, social) and information on exposures and potential confounders	✓
		(b) Indicate number of participants with missing data for each variable of interest	✓
		(c) Summarise follow-up time (eg, average and total amount)	✓

For peer review only

Outcome data	15*	Report numbers of outcome events or summary measures over time	✓
Main results	16	(*a*) Give unadjusted estimates and, if applicable, confounder-adjusted estimates and their precision (eg, 95% confidence interval). Make clear which confounders were adjusted for and why they were included	✓
		(*b*) Report category boundaries when continuous variables were categorized	✓
		(*c*) If relevant, consider translating estimates of relative risk into absolute risk for a meaningful time period	N/A
Other analyses	17	Report other analyses done—eg analyses of subgroups and interactions, and sensitivity analyses	✓
**Discussion**			
Key results	18	Summarise key results with reference to study objectives	✓
Limitations	19	Discuss limitations of the study, taking into account sources of potential bias or imprecision. Discuss both direction and magnitude of any potential bias	✓
Interpretation	20	Give a cautious overall interpretation of results considering objectives, limitations, multiplicity of analyses, results from similar studies, and other relevant evidence	✓
Generalisability	21	Discuss the generalisability (external validity) of the study results	✓
**Other information**			
Funding	22	Give the source of funding and the role of the funders for the present study and, if applicable, for the original study on which the present article is based	✓

*Give information separately for exposed and unexposed groups.

Note: An Explanation and Elaboration article discusses each checklist item and gives methodological background and published examples of transparent reporting. The STROBE checklist is best used in conjunction with this article (freely available on the Web sites of PLoS Medicine at http://www.plosmedicine.org/, Annals of Internal Medicine at http://www.annals.org/, and Epidemiology at http://www.epidem.com/). Information on the STROBE Initiative is available at http://www.strobe-statement.org.

For peer review only - http://bmjopen.bmj.com/site/about/guidelines.xhtml

BMJ Open

Direct and Mediated Effects of Treatment Context on Low Back Pain Outcome: A Prospective Cohort Study

Journal:	*BMJ Open*
Manuscript ID	bmjopen-2020-044831.R1
Article Type:	Original research
Date Submitted by the Author:	23-Feb-2021
Complete List of Authors:	Bishop, Felicity; University of Southampton, Psychology<br>Al-Abbadey, Miznah; University of Portsmouth, Department of Psychology<br>Roberts, Lisa; University of Southampton, Health Sciences; University Hospital Southampton NHS Foundation Trust, Therapy Services<br>MacPherson, Hugh; University of York, Health Sciences<br>Stuart, Beth; University of Southampton, Primary Care and Population Sciences<br>Carnes, Dawn; Queen Mary University of London, Institute of Population Health Sciences<br>Fawkes, Carol; Queen Mary University of London, Institute of Population Health Sciences<br>Yardley, Lucy; University of Southampton, Psychology; University of Bristol, School of Health Sciences<br>Bradbury, Katherine; University of Southampton, Psychology
**Primary Subject Heading**:	Rehabilitation medicine
Secondary Subject Heading:	Complementary medicine, Patient-centred medicine
Keywords:	REHABILITATION MEDICINE, PRIMARY CARE, Back pain < ORTHOPAEDIC & TRAUMA SURGERY, COMPLEMENTARY MEDICINE

SCHOLARONE™
Manuscripts

I, the Submitting Author has the right to grant and does grant on behalf of all authors of the Work (as defined in the below author licence), an exclusive licence and/or a non-exclusive licence for contributions from authors who are: i) UK Crown employees; ii) where BMJ has agreed a CC-BY licence shall apply, and/or iii) in accordance with the terms applicable for US Federal Government officers or employees acting as part of their official duties; on a worldwide, perpetual, irrevocable, royalty-free basis to BMJ Publishing Group Ltd ("BMJ") its licensees and where the relevant Journal is co-owned by BMJ to the co-owners of the Journal, to publish the Work in this journal and any other BMJ products and to exploit all rights, as set out in our licence.

The Submitting Author accepts and understands that any supply made under these terms is made by BMJ to the Submitting Author unless you are acting as an employee on behalf of your employer or a postgraduate student of an affiliated institution which is paying any applicable article publishing charge ("APC") for Open Access articles. Where the Submitting Author wishes to make the Work available on an Open Access basis (and intends to pay the relevant APC), the terms of reuse of such Open Access shall be governed by a Creative Commons licence – details of these licences and which Creative Commons licence will apply to this Work are set out in our licence referred to above.

Other than as permitted in any relevant BMJ Author's Self Archiving Policies, I confirm this Work has not been accepted for publication elsewhere, is not being considered for publication elsewhere and does not duplicate material already published. I confirm all authors consent to publication of this Work and authorise the granting of this licence.

For peer review only

Direct and Mediated Effects of Treatment Context on Low Back Pain Outcome: A Prospective Cohort Study

Felicity L Bishop,[1*] Miznah Al-Abbadey, [1,2] Lisa Roberts, [3,4] Hugh MacPherson, [5] Beth Stuart, [6] Dawn Carnes, [7] Carol Fawkes, [7] Lucy Yardley, [1,8] Katherine Bradbury.[1]

[1] Department of Psychology, University of Southampton, Southampton, UK.
[2] Department of Psychology, University of Portsmouth, Portsmouth, UK.
[3] Health Sciences, University of Southampton, Southampton, UK.
[4] Therapy Services, University Hospital Southampton NHS Foundation Trust, Southampton, UK
[5] Health Sciences, University of York, York, UK.
[6] Primary Care and Population Sciences, University of Southampton, Southampton, UK.
[7] Institute of Population Health Sciences, Queen Mary University of London, London, UK.
[8] School of Psychological Science, University of Bristol, Bristol, UK

*Correspondence concerning this article should be addressed to Felicity L Bishop. Centre for Clinical and Community Applications of Health Psychology, Faculty of Environmental and Life Sciences, Building 44 Highfield Campus, University of Southampton, Southampton SO17 1BJ United Kingdom. Email: F.L.Bishop@southampton.ac.uk.

Word count, excluding title page, abstract, references, figures and tables: 4582

For peer review only

Abstract

Objectives: Contextual components of treatment previously associated with patient outcomes include the environment, therapeutic relationship, and expectancies. Questions remain about which components are most important, how they influence outcomes and comparative effects across treatment approaches. We aimed to identify significant and strong contextual predictors of patient outcomes, test for psychological mediators, and compare effects across three treatment approaches.

Design: Prospective cohort study with patient-reported and practitioner-reported questionnaire data (online or paper) collected at first consultation, 2-weeks, and 3-months.

Setting: Physiotherapy, osteopathy, and acupuncture clinics throughout the UK.

Participants: 166 practitioners (65 physiotherapists, 46 osteopaths, 55 acupuncturists) were recruited via their professional organisations. Practitioners recruited 960 adult patients seeking treatment for low back pain (LBP).

Primary and Secondary Outcomes: The primary outcome was back-related disability. Secondary outcomes were pain and well-being. Contextual components measured were: therapeutic alliance; patient satisfaction with appointment systems, access, facilities; patients' treatment beliefs including outcome expectancies; practitioners' attitudes to LBP; and practitioners' patient-specific outcome expectancies. The hypothesised mediators measured were: patient self-efficacy for pain management; patient perceptions of LBP; and psychosocial distress.

Results: After controlling for baseline and potential confounders, statistically significant predictors of reduced back-related disability were: all three dimensions of stronger therapeutic alliance (goal, task and bond); higher patient satisfaction with appointment systems; reduced patient-perceived treatment credibility; and increased practitioner-rated outcome expectancies. Therapeutic alliance over task (η_p^2=0.10 95% CI 0.07-0.14) and practitioner-rated outcome expectancies (η_p^2=0.08 95% CI 0.05-0.11) demonstrated the largest effect sizes. Patients' self-efficacy, LBP perceptions, and

For peer review only

psychosocial distress partially mediated these relationships. There were no interactions with treatment approach.

Conclusions: Enhancing contextual components in musculoskeletal healthcare could improve patient outcomes. Interventions should focus on helping practitioners and patients forge effective therapeutic alliances with strong affective bonds and agreement on treatment goals and how to achieve them.

Keywords: health knowledge, attitudes, practice; low back pain; placebo effects; therapeutic relationship; complementary therapies; context.

Article Summary

- The prospective but non-experimental design requires cautious interpretation regarding possible causal relationships between variables.
- 97% of the sample size was achieved (960 vs 986), with higher retention than anticipated (77% vs 70%) and the final sample at follow-up exceeded that required a priori (742 vs 690).
- The geographical spread across the UK and the large cohort size suggests the results may be generalizable beyond this sample.
- All variables were measured using self-report questionnaires, potentially introducing social desirability and/or shared-method bias; the former was partially mitigated by patients returning questionnaires directly to the researchers, not their clinicians; the latter was partially mitigated by the inclusion of patient and practitioner completed questionnaires and the separation in time of the measurement of predictors and outcomes.

For peer review only

INTRODUCTION

All healthcare interventions are delivered within a context that may significantly enhance or hinder an intervention's beneficial effects. Furthermore, contextual components, such as features of the environment or the patient's beliefs or "mind-set,"[1 2] may be shared across similar therapies. Therefore, identifying and strengthening beneficial contextual components could offer an efficient means of enhancing the effectiveness of multiple interventions. Low back pain (LBP) was chosen as the condition of interest as it is highly prevalent with considerable burden to individuals, communities, health services and the economy[3] and there is scope and need to improve patient outcomes across multiple non-pharmacological treatments. However, before embarking on intervention development and testing, it is vital to determine which contextual components should be targeted to produce the greatest likelihood of patient benefit.

Contextual components comprise the broad constellation of psychological, social, and environmental factors that act alongside and can interact with core components of interventions. Five domains have been proposed: patient-practitioner relationship; patients' beliefs; practitioners' beliefs; healthcare environment; and, for pharmacological interventions, incidental characteristics of treatment such as the colour of medications.[4] Particular components have been associated with improved patient satisfaction and/or clinical outcomes across multiple settings or specifically within non-pharmacological musculoskeletal care. Such components include: positive and empathetic patient-practitioner relationships and a strong patient-practitioner alliance[4-10] (patient-practitioner relationship); patients' outcome expectancies and perceived treatment credibility[9 11-16] (patient beliefs); musculoskeletal practitioners' beliefs about pain[17-22] and outcome expectancies[23] (practitioner beliefs); good organisational environments (e.g. collegiality)[24] the physical-sensory environment (e.g. music)[25] and the healthcare sector[26-28] (environment), although evidence of effects of healthcare sector on patient outcomes is mainly qualitative. Contextual components might impact patient outcomes via i) behavioural pathways (e.g. improved self-management,[29 30] but see also Haanstra et al. who found that adherence accounted for only a small proportion of the

For peer review only

relationship between expectancies and pain outcomes[31] ii) psychosocial pathways (e.g. reconceptualising pain as less threatening;[32 33 34] increased self-efficacy[35 36]), and/or iii) neurophysiological pathways.[37-41] The focus in this study was on psychosocial pathways; more details on how these were hypothesised to operate are provided in our published protocol.[42]

In summary, there is good evidence that contextual components can impact patient outcomes. However, most studies have focused on one or two components, meaning that direct comparisons have not been made to ascertain the relative contribution of different contextual components to patient health outcomes. Few studies have quantitatively tested potential pathways in the context of usual clinical practice. Most studies have focused on one treatment, meaning that direct comparisons have not been made to compare contextual components and their effects across treatment approaches. Therefore, the purpose of this study was to investigate the effects and mediating pathways of multiple contextual components on patient outcomes across three different treatment approaches in the private and public sectors. Physiotherapy, osteopathy, and acupuncture were selected as they are popular, safe, and relatively effective approaches to treating LBP[43-45] and the core treatment modalities used by physiotherapists, osteopaths, and acupuncturists, were recommended by clinical guidelines at the conception of this study.[46 47]

The aims of the study were to (1) identify contextual components of treatment for LBP that predict patient outcomes, (2) test whether psychosocial factors mediate effects of contextual components on patient outcomes, and (3) compare components and their effects across physiotherapy, osteopathy, and acupuncture. In relation to aim (1), based on the literature it was hypothesised that patients would experience less back-related disability after treatment for LBP when contextual components are more positive (i.e., stronger therapeutic alliance; more pleasant/ accessible/ convenient/ supportive healthcare environment; positive patient-reported treatment beliefs and outcome expectancies; positive practitioner-reported outcome expectancies and practitioner having a biopsychosocial orientation to LBP). However, there were no strong grounds for a priori hypotheses concerning which component would be the strongest predictor of patient

For peer review only

outcomes. In relation to aim (2), it was hypothesised that contextual components reduce patients' back-related disability by reducing the perceived threat of pain and/or increasing patients' self-efficacy for coping with pain and/or reducing psychosocial distress. For aim (3), the effects of contextual components on patient outcomes were hypothesised to be larger in osteopathy and acupuncture – as examples of what have been classed complementary therapies - than in physiotherapy, which is usually considered a conventional therapy. This hypothesis was based on evidence that patients find the context of complementary therapies particularly valuable,[48] that the consultation process used by acupuncturists is effective for Irritable Bowel Syndrome even when accompanied by a sham version of acupuncture [8] and that the rituals and modes of delivery of complementary therapies may enhance their effects.[49]

METHODS

Ethical Considerations

Ethical approval was obtained from NHS Health Research Authority NRES Committee East Midlands – Derby (REF: 14/EM/1113) on 22nd August 2014. Participants gave written informed consent; study documents conveyed the right to decline or withdraw without affecting medical care.

Design

In this prospective cohort study, practitioners and patients completed questionnaires at three time-points: baseline (after the first consultation for a new episode of LBP); during treatment (2-weeks post-baseline); and short-term outcome (3-months post-baseline). Contextual components were measured once each either at baseline or 2-weeks (specified in Measures), with time-points chosen to reduce ceiling effects, capture data accurately, and spread questionnaire burden. Outcomes, potential predictors of outcome, and potential mediators were measured repeatedly at baseline, 2-weeks and 3-months, to permit tests of whether scores on contextual factors were associated with changes over time in predictors or mediators. Participants chose to

For peer review only

complete hard copies (mailed, returned via Freepost) or electronic copies (emailed, completed online at isurvey.soton.ac.uk). Online and paper versions of the primary outcome are equivalent, and used interchangeably, as patients value having this choice.[50] The full protocol was published.[42] See Supplemental Material Table 1 for deviations from the protocol.

Procedure

Between April 2015 and April 2017, physiotherapists, osteopaths, and acupuncturists working in the NHS and the private sector throughout the UK were recruited by advertisements and personal invitations, with support from professional societies. Inclusion criteria were: treat at least one patient with LBP on average per week, have at least three years' current experience in musculoskeletal work, and be registered (or eligible to register, for acupuncturists) with the appropriate professional body. To better capture current clinical practice, no restrictions were placed on type of intervention being delivered, meaning that osteopaths and physiotherapists were able to use acupuncture if and when they would usually do so.

Written consent was obtained from eligible practitioners, who gave potentially eligible patients a study invitation pack (containing invitation letter, information sheet, consent form, and baseline questionnaire). Patients were recruited between May 2015 and April 2017. On inviting an eligible patient into the study, practitioners rated their outcome expectancy for that patient on an anonymised form, labelled only with the same unique identifier as the corresponding patient invitation pack. Each outcome expectancy form was only analysed if the corresponding patient consent to participate was received by the researchers. Inclusion criteria were: at least 18 years old, seeking treatment from a participating practitioner at their first consultation for a new episode of LBP, and score at least 4/24 on the Roland-Morris Disability Questionnaire (RMDQ).[51] Exclusion criteria were: unable to complete questionnaires in English or Welsh, have serious underlying pathology (inflammatory arthritis, malignancy), or have practitioner-identified conditions that would prevent the sought treatment being applied. Arguably, LBP as managed in primary care should be

For peer review only

conceptualised as typically having a chronic-episodic timeline with recurrent acute flare-ups against a backdrop of temporary remission or less bothersome symptoms.[52 53] Broad inclusion criteria therefore best reflect the clinical situation.

Completed consent forms and questionnaires were returned directly to the researchers via prepaid envelopes. The researchers emailed or posted (participant's choice) the 2-week and 3-months questionnaires to participants. Techniques used to enhance retention were: non-response follow-ups (resending questionnaires and telephoning); monthly study e-newsletters to practitioners; small gifts (e.g. tea bag/pen) and monetary incentives (£5 voucher) for patients; personalised questionnaires; ink-signed cover letters; coloured ink; stamped return envelopes; first class post.[54]

Measures

This section describes how each construct was operationalised, explains the choice of questionnaires and the timing of each measure. Where other measures of constructs existed, questionnaires were chosen for their conceptual fit, psychometric properties, and conciseness. Supplemental Material Table 2 provides additional details including: example items, response scales, and Cronbach's alphas.

Outcomes

The primary outcome was self-reported back-related disability, measured using the 24-item Roland-Morris Disability Questionnaire (RMDQ).[51] Secondary outcomes (pain intensity, well-being, work and social role disability, satisfaction with care) were measured using recommended core single items.[55] All primary and secondary outcomes were measured at baseline, 2-weeks, and 3-months.

For peer review only

Contextual Components

Patient-Practitioner Relationship

The patient-practitioner relationship was operationalised in this study as the therapeutic alliance. The therapeutic alliance is conceptually grounded in theory on how patient-practitioner interactions can elicit psychological and/or behavioural changes[56] both of which may be important in LBP. Therapeutic alliance was assessed using the 12-item patient-reported Working Alliance Inventory Short Form (WAI-SF), which measures three dimensions of working alliance – task collaboration, affective bond, and goal concordance - (four items each) with acceptable psychometric properties.[57] Task collaboration refers to the extent to which patients feel that the treatment approach their practitioner is taking is the right one for them. Affective bond refers to the extent to which patients feel an interpersonal connection with their practitioner including mutual respect and liking. And goal concordance refers to the extent to which patients feel they agree with their practitioner on the outcomes they are aiming to achieve through treatment. The patient-reported WAI-SF may have ceiling effects if used after one treatment but tends to remain stable after the second treatment;[6] it was therefore administered at 2-weeks.

The Healthcare Environment

Practitioners' perceptions of the organisational environment were measured at baseline using two sub-scales from the psychometrically sound Attitudes to Back Pain Scale – Musculoskeletal Practitioners (ABS-mp): putting limits on sessions (4 items) and perceived connections within the health care system (3 items).[17 58] Patients' perceptions of the organisational and sensory-physical environment were assessed at 2-weeks using three sub-scales of the Patient Satisfaction Questionnaire (PSQ) designed to assess patient perceptions of the quality of primary healthcare in the UK.[59] The access sub-scale measures perceptions of interactions with reception staff (8 items); the appointments sub-scale measures perceived availability of convenient appointments (4 items);

For peer review only

the facilities sub-scale measures perceptions of the physical environment of the clinic and waiting room (4 items).

Patients' Beliefs

Four dimensions of treatment beliefs were measured using the brief LBP Treatment Beliefs Questionnaire: expected effectiveness, credibility, concerns, and individualised fit (4 items per subscale).[60] This questionnaire was explicitly designed for use in mixed cohorts of patients with LBP undergoing diverse treatments. It was completed at baseline as patients' expectancies regarding effectiveness should be measured early in treatment.[13]

Practitioners' Beliefs

A single-item numerical rating scale at baseline measured practitioners' outcome expectancies for each patient. Four sub-scales from the ABS-mp measured, at baseline, practitioners': willingness to engage with psychological issues (4 items); confidence and concern over clinical limitations (2 items); re-activation of work and activity (3 items); and belief in an underlying structural cause of pain (3 items).[17 58]

Hypothesised Mediators

Hypothesised mediators, i.e. constructs thought to be on the causal pathway between contextual components and patient outcomes, were measured at baseline, 2-weeks, and 3-months. The reliable and valid 9-item brief illness perceptions questionnaire (BIPQ) was used to measure the extent to which patients perceive their LBP as threatening.[61] Self-efficacy for coping with pain was assessed using the 5-item Chronic Pain Self-Efficacy for Pain Management sub-scale.[62] Psychosocial distress was assessed using the 5-item Psychosocial scale on the STarT Back questionnaire[63] which assesses multiple modifiable psychosocial predictors of outcome.

For peer review only

Clinical and Socio-Demographic Characteristics

Patient and practitioner characteristics were assessed at baseline. Patient characteristics were: leg pain and shoulder/neck pain bothersomeness (measured using the single item on bothersomeness as worded on the STarT Back tool[63] adapted from[64]); LBP duration; age; gender; work status; compensation status; co-morbidities; co-treatments; socio-economic status. To indicate socio-economic status, patients' postcodes were collected and used to assign the associated index of multiple deprivation (IMD) score to assign to each patient. The IMD ranks 32,844 small areas in England from most to least deprived; scores from 1 to 10 are then assigned to each area indicating the decile of deprivation, and these can be looked up by postcode. An IMD score of 1 indicates the area is among the most deprived 10% of areas.[65] Practitioner characteristics were: time since qualifying; experience in musculoskeletal care; age; gender.

Practitioners reported for each patient individually at three months which treatment modalities had been applied (reported in Supplemental Material Table 3).

Statistical Analysis

Power Calculation

The planned multilevel analysis required a final sample size of 690 patients to detect correlations between the predictors, hypothesised mediators, and the primary outcome with Pearson's R = 0.2 (and corresponding Beta regression coefficients of 0.235) with 95% power and p<0.0005 (2-tailed). This sample size was adjusted for clustering of patients within practitioners: the basic sample size was inflated by a factor of 1 +(k-1)*ICC, where K = 8 and ICC = 0.01.[66] (K = mean patients recruited per practitioner, ICC = intra-cluster correlation.) It was also intended to allow testing for interactions and possible treatment-specific effects. The target sample size required 86 practitioners to actively recruit patients (29 practitioners per therapy). To allow for 30% dropout, based on a similar prospective cohort study in acupuncture,[15] we aimed to recruit 986 patients.

For peer review only - http://bmjopen.bmj.com/site/about/guidelines.xhtml

Data Entry

Paper questionnaires were entered online by researchers and checked for accuracy. All data were downloaded into MS Excel, cleaned, and exported into SPSS v22 and Stata for analysis.

Analysis

Data were explored using descriptive statistics and graphs. Cronbach's alpha was calculated to summarise the internal consistency of the measures at baseline. No imputation was undertaken for missing values.

To test the extent to which each contextual component predicted patient outcomes over time (Aim 1), multi-level linear mixed models were run with observations nested within patients over time and patients nested within practitioners. Separate models were run to test the effect of each individual contextual component on patient outcomes. These models excluded hypothesised mediators but did control for baseline back-related disability and other potential confounders specified in the protocol: leg pain and shoulder/neck pain bothersomeness; duration of LBP; age; gender; work status; compensation status; co-morbidities; co-treatments; socio-economic status (using deprivation index as indicated by post-code). The effect size was calculated for significant predictors using the partial eta-squared for multivariate regression, which can be interpreted as follows: 0.02 is a small effect, 0.13 moderate and 0.26 large.[67]

To test whether the effect of contextual components on patient outcomes was mediated by perceived threat of pain, self-efficacy for coping and/or psychosocial distress (Aim 2), the Baron and Kenny approach was used.[68] This involved (1) testing whether each hypothesized mediator was associated with the outcome (RMDQ), and then (2) controlling for those hypothesized mediators that were associated with the outcome in the analyses of those contextual components which had a statistically significant relationship with the outcome. This enabled a determination of whether the relationship between the contextual components and outcome remained significant or whether the

For peer review only

relationship was largely explained by the presence of the mediators. Mediators were included in models as repeated measures.

To compare the effects of contextual components on patient outcomes across osteopathy, acupuncture, and physiotherapy (Aim 3), the presence of significant interactions between contextual components and treatment type was tested.

Patient Public Involvement

One volunteer, with personal experience of back pain and clinical research, acted as a patient advisor in this study. She attended team meetings and was involved in decision-making around study procedures and conduct including advising on the design of patient-facing study documents. She also wrote to participants to thank them for taking part. Involving members of the public in the management of research helps to improve researchers' awareness of participants' needs and perspectives and can enhance the design and conduct of studies.[69]

RESULTS

Participants

Overall, 166 practitioners and 960 patients participated. On average, each practitioner recruited 5.7 patients (SD=8.2). Figure 1 shows overall participant flow; this is split by treatment group in Supplemental Material Figure 1.

Table 1 shows patient characteristics at baseline, split by treatment group. Most patients were female, university-educated, resided in the 50% least deprived neighbourhoods in the country, had LBP for up to 3 months, did not have a significant comorbidity, and were using additional treatment(s). The group of patients accessing physiotherapy on the NHS differed from the other groups, they were less likely to hold postgraduate qualifications, more likely to be unemployed or on sick leave, more likely to have chronic LBP and more likely to report a compensation or other legal claim related to their LBP (N.B. absolute numbers of claims were low).

For peer review only

EFFECTS OF TREATMENT CONTEXT

As a cohort, practitioners had been working for 20 years since qualifying (M=20.6, SD=10.1) and felt they were highly experienced at treating patients with LBP (M=4.3, SD=0.8). Compared to the other groups, physiotherapists working in the NHS had been working for less years since qualifying (M=15.5, SD=7.2) and rated themselves as less experienced with LBP (M=3.8, SD=1.3). Supplemental Material Table 4 presents full practitioner characteristics by treatment group.

For peer review only

Table 1. Characteristics at Baseline of Patients with a New Episode of Low Back Pain, for the Whole Sample and for Subgroups of Patients Receiving Physiotherapy in the NHS, Physiotherapy in the Private Sector, Osteopathy, and Acupuncture, n (%)

	Whole sample	Physiotherapy NHS	Physiotherapy Private Sector	Osteopathy	Acupuncture
Practitioners					
Total	166 (100%)	36 (21.7%)	29 (17.5%)	46 (27.7%)	55 (33.1%)
Patients					
Total	960 (100%)	196 (20.4%)	165 (17.2%)	394 (41.0%)	205 (21.4%)
Age (years): M (SD)	52.3 (14.7)	52.4 (15.2)	55.4 (14.6)	57.3 (14.3)	56.0 (15.0)
Gender					
Female	605 (63.0%)	127 (64.8%)	103 (62.4%)	238 (60.4%)	137 (66.8%)
Male	338 (35.2%)	65 (33.2%)	61 (37.0%)	151 (38.3%)	61 (29.8%)
Missing data	17 (1.8%)	4 (2.0%)	1 (0.6%)	5 (1.3%)	7 (3.4%)
Education					
Up to secondary	39 (4.1%)	12 (6.1%)	1 (0.6%)	18 (4.6%)	8 (3.9%)
Finished secondary	306 (31.9%)	72 (36.73%)	39 (23.6%)	128 (32.5%)	67 (32.7%)
Finished 6th form	214 (22.3%)	51 (26.0%)	35 (21.2%)	94 (23.9%)	34 (16.6%)
Undergraduate	258 (26.9%)	40 (20.4%)	62 (37.6%)	97 (24.6%)	59 (28.8%)
Postgraduate	123 (12.8%)	13 (6.6%)	27 (16.4%)	51 (12.9%)	32 (15.6%)
Missing data	20 (2.1%)	8 (4.1%)	1 (0.6%)	6 (1.5%)	5 (2.4%)
Work status					
Employed	527 (54.9%)	116 (59.2%)	92 (55.8%)	211 (53.6%)	108 (52.7%)
Unemployed/sick leave	65 (6.8%)	22 (11.2%)	8 (4.8%)	22 (5.6%)	13 (6.3%)
Student/homemaker	60 (6.3%)	11 (5.6%)	14 (8.5%)	20 (5.1%)	15 (7.3%)
Retired	280 (29.2%)	43 (21.9%)	50 (30.3%)	125 (31.7%)	62 (30.2%)
Missing data	28 (2.9%)	4 (2.0%)	1 (0.6%)	16 (4.1%)	7 (3.4%)
Healthcare Sector					
Private Sector	742 (77.3%)	0	165 (100%)	389 (98.7%)	188 (91.7%)

For peer review only

EFFECTS OF TREATMENT CONTEXT

NHS	218 (22.7%)	196 (100%)	0	5 (1.3%)	17 (8.3%)
Duration of back pain					
Up to 1 week	127 (13.2%)	8 (4.1%)	37 (22.4%)	67 (17.0%)	15 (7.3%)
1 week – 1 month	257 (26.8%)	20 (10.2%)	50 (30.3%)	140 (35.5%)	47 (22.9%)
1 month -3 months	179 (18.6%)	41 (20.9%)	33 (20.0%)	77 (19.5%)	28 (13.7%)
3 months – 1 year	158 (16.5%)	57 (29.1%)	23 (13.9%)	48 (12.2%)	30 (14.6%)
1 year +	214 (22.3%)	62 (31.6%)	20 (12.1%)	55 (14.0%)	77 (37.6%)
Missing data	25 (2.6%)	8 (4.1%)	2 (1.2%)	7 (1.8%)	8 (3.9%)
Number of previous episodes					
None	210 (21.9%)	58 (29.6%)	33 (20.0%)	64 (16.2%)	55 (26.8%)
5 or fewer	317 (33.0%)	62 (31.6%)	60 (36.4%)	147 (37.3%)	48 (23.4%)
More than 5	349 (36.4%)	53 (27.0%)	62 (37.6%)	154 (39.1%)	80 (39.0%)
Missing data	84 (8.8%)	23 (11.7%)	10 (6.1%)	29 (7.4%)	22 (10.7%)
Comorbidity					
Longstanding illness or disability (besides LBP)	321 (33.4%)	71 (36.2%)	51 (30.9%)	119 (30.2%)	80 (39.0%)
Missing data	17 (1.8%)	7 (3.6%)	1 (0.6%)	8 (2.0%)	5 (2.4%)
Co-treatment					
Currently using other treatments	562 (58.5%)	123 (62.8%)	89 (53.9%)	215 (54.6%)	135 (65.9%)
Missing data	17 (1.8%)	10 (5.1%)	2 (1.2%)	12 (3.0%)	4 (2.0%)
Compensation					
Compensation or legal claim for LBP	27 (2.8%	13 (6.6%)	5 (3.0%)	6 (1.5%)	3 (1.5%)
Missing data	17 (1.8%)	4 (2.0%)	1 (0.6%)	6 (1.5%)	4 (2.0%)

Note. NHS = National Health Service. LBP = Low Back Pain.

For peer review only

Outcomes over Time

On average, patients improved from baseline to 2-weeks and from 2-weeks to 3-months on all primary and secondary outcome measures, except for overall satisfaction with healthcare which remained similarly high at all measurement points. Figure 2 displays the change in back-related disability over time; outcomes by treatment group and secondary outcomes are provided in Supplemental Material Figure 2 and Supplemental Material Table 5 respectively.

Contextual Predictors of Back-Related Disability over Time

Table 2 shows the results of models assessing the relationship between each contextual component and back-related disability over time. After controlling for baseline disability and potential confounders, statistically significant predictors of reduced back-related disability over time were: stronger therapeutic alliance on all three dimensions; higher patient satisfaction with appointment systems; reduced perceived credibility of chosen treatment; and increased practitioner-rated outcome expectancies.

Therapeutic alliance concerning task and practitioner-rated outcome expectancies demonstrated the largest effect sizes. For each unit increase in patients' rating of the therapeutic alliance (task), patient-rated back-related disability over time decreased by 2.33 points (95% CI -2.89 to -1.77) on the RMDQ. For each unit increase in practitioners' patient-specific outcome expectancies, patient-rated back-related disability over time decreased by 1.29 points (95% CI -1.66 to -0.92) on the RMDQ.

Mediation Analysis to Examine Mechanisms

All the mediators were significantly associated with the outcome (for descriptive statistics see Supplemental Material Table 6). For each unit increase in self-efficacy for coping with pain, RMDQ improved by 0.07 (95% CI -0.08, -0.05). As psychosocial distress increased by one unit, RMDQ increased by 1.43 (95% CI 1.09, 1.77). And as overall perception of back pain as threatening

For peer review only

increased by one unit, RMDQ score increased by 0.23 (95% CI 0.21, 0.25). After controlling for these three variables in the longitudinal multilevel models, the measures of therapeutic alliance, satisfaction with appointment systems, and reduced perceived credibility of treatment were no longer statistically significant predictors of RMDQ (Table 2). This suggests that their effects on disability are indeed mediated by increased self-efficacy for coping with pain, reduced perception of back pain as threatening, and reduction in psychosocial distress.

Practitioners' outcome expectancies at baseline showed reduced but still statistically significant effects on RMDQ changes over time, suggesting that their effects are only partially mediated. In all cases the mediators remained associated with the outcome in the multivariable models, except for self-efficacy, which was not statistically significant in the model with practitioners' outcome expectancies. This suggests that the effect of practitioners' outcome expectancies at baseline on changes in RMDQ over time was partially mediated by changes in perception of back pain as threatening and psychosocial distress. For a visual summary of the general mediation model tested, see Supplemental Material Figure 3.

For peer review only

EFFECTS OF TREATMENT CONTEXT

Table 2. Longitudinal Models Assessing Contextual Predictors of Back-Related Disability over Time in Patients Receiving Physiotherapy, Osteopathy, or Acupuncture for a New Episode of Low Back Pain

Contextual component	Possible scale range	Mean (SD)	Change in RMDQ for one-unit change in component: Unadjusted for confounders	Adjusted for confounders[a]	Adjusted for mediators[b]	Effect size partial eta-squared (95% CI)[c]
Therapeutic alliance: goal	1-5	4.2 (0.7)	-1.14 (-1.69, -0.60)	-0.94 (-1.54, -0.35)	0.36 (-0.09, 0.82)	0.02 (0.004, 0.04)
Therapeutic alliance: task	1-5	4.0 (0.6)	-2.78 (-3.33, -2.23)	-2.33 (-2.89, -1.77)	0.13 (-0.34, 0.61)	0.10 (0.07, 0.14)
Therapeutic alliance: bond	1-5	4.1 (0.9)	-1.14 (-1.56, -0.32)	-0.86 (-1.30, -0.43)	-0.10 (-0.43, 0.23)	0.03 (0.01, 0.05)
Environment: Limits on Treatment	4-28	11.5 (2.9)	-0.08 (-0.25, 0.09)	-0.03 (-0.23, 0.16)		
Environment: Connections within the Healthcare System	3-21	11.3 (2.3)	0.01 (-0.23, 0.25)	-0.05 (-0.27, 0.17)		
Environment: Satisfaction with appointment systems	1-5	1.9 (0.7)	1.31 (0.81, 1.82)	0.67 (0.13, 1.20)	-0.23 (-0.65, 0.14)	0.02 (0.01, 0.04)
Environment: Satisfaction with access	1-5	2.5 (0.6)	1.01 (0.38, 1.63)	0.60 (-0.04, 1.26)		
Environment: Satisfaction with facilities	1-5	2.2 (0.8)	0.52 (0.04, 1.01)	0.34 (-0.18, 0.86)		
Patient's beliefs: Perceived credibility of treatment	1-5	3.0 (0.3)	1.34 (0.19, 2.50)	1.51 (0.35, 2.67)	0.58 (-0.28, 1.44)	0.01 (0.001, 0.03)
Patient's beliefs: Expectancies for treatment effectiveness	1-5	2.8 (0.3)	0.17 (-0.99, 1.34)	0.12 (-1.05, 1.29)		
Patient's beliefs: Concerns about treatment	1-5	2.9 (0.3)	-0.36 (-1.45, 0.73)	-0.26 (-1.37, 0.86)		
Patient's beliefs: Individualised fit of treatment	1-5	2.9 (0.3)	0.14 (-1.07, 1.34)	0.59 (-0.65, 1.83)		
Practitioner's beliefs: Psychological	4-28	17.8 (3.6)	0.13 (-0.06, 0.32)	0.03 (-0.15, 0.20)		
Practitioner's beliefs: Confidence	2-14	8.2 (2.0)	0.03 (-0.27, 0.32)	0.10 (-0.19, 0.40)		
Practitioner's beliefs: Reactivation	3-21	15.3 (3.0)	-0.06 (-0.24, 0.13)	0.08 (-0.10, 0.25)		
Practitioner's beliefs: Biomedical	3-21	12.2 (3.6)	-0.06 (-0.22, 0.10)	-0.07 (-0.25, 0.11)		
Practitioner's beliefs: Outcome expectancies	1-7	5.7 (1.1)	-1.77 (-2.12, -1.42)	-1.29 (-1.66, -0.92)	-0.42 (-0.70, -0.13)	0.08 (0.05, 0.11)

Note. Table reports the results of separate models run to test the effect of each individual contextual factor on patient outcomes, specifically back-related disability. RMDQ = Roland Morris Disability Questionnaire. SD = Standard Deviation. [a] Adjusted for baseline back-related disability and: leg pain and shoulder/neck pain bothersomeness; duration of LBP; age; gender; work status; compensation status; co-morbidities; co-treatments; socio-economic status.

For peer review only

EFFECTS OF TREATMENT CONTEXT

[b] Adjusted for mediation by perceived threat of pain, self-efficacy for coping and psychosocial distress. [b] Effect size only reported for statistically significant contextual predictors of back-related disability over time.

For peer review only

Comparisons across Treatments

The longitudinal models revealed no significant interactions between contextual factors and treatment type (see Supplemental Material Table 7). This indicates that the effects of contextual components on back-related disability do not differ significantly between osteopathy, acupuncture, and physiotherapy.

DISCUSSION

This large prospective cohort study investigated the effects and mediating pathways of multiple contextual components on patient outcomes across three different therapies in the private and public sectors. As hypothesised, components representing four non-pharmacological contextual domains were significantly associated with back-related disability over time in a large cohort of patients with LBP seeking osteopathy, acupuncture, and physiotherapy. The patient-practitioner therapeutic alliance and practitioners' expectancies of how individual patients will respond to treatment were the strongest contextual components, showing the largest effects on patient outcomes. These components reduced disability at least partly by improving patients' self-efficacy for coping and reducing psychosocial distress and the perceived threat of LBP. Other contextual components emerged as statistically significant predictors of improved outcome over time: the healthcare environment, specifically greater satisfaction with appointments; and (contrary to hypothesised direction of association) patients' beliefs, specifically reduced perceived credibility of one's chosen treatment. The relationship between contextual components and patient outcomes did not differ across osteopathy, acupuncture, and physiotherapy.

The current study extends understanding of the mechanisms through which contextual factors may impact patient health outcomes. Earlier studies have investigated pathways amongst smaller numbers of contextual variables and suggested, for example, that self-efficacy mediates the impact on osteoarthritis pain of practitioners communicating positive expectancies,[35] affective aspects of the therapeutic relationship may shape patient expectancies in chronic pain,[70] and

For peer review only

expectancies might impact pain via reduced catastrophising in chronic back pain.[33] The current results further suggest contextual factors reduce back-related disability in part by enhancing patient self-efficacy for coping with pain, reducing the perceived threat of pain, and reducing psychosocial distress. This is broadly consistent with the fear avoidance model[34] and social cognitive theory.[36] It also builds on work in adjacent areas such as communication[41] and starts to map potential evidence-based psychological pathways through which contextual factors might impact patient outcomes.

The prospective but non-experimental design requires cautious interpretation regarding possible causal relationships between variables: the results provide evidence of predictive relationships between variables over time, which are consistent with, but insufficient to establish causality. Experimental designs (e.g., trials) are now required to build on this work and to confirm the clinical importance of therapeutic alliance as a factor that influences patient outcomes. While 97% of the baseline sample size was achieved (960 vs 986), retention was higher than anticipated (77% vs 70%) and the final sample at follow-up exceeded that required a priori (108%, n=742 vs 690). The geographical spread across the UK and the large cohort size suggests the results may be generalizable beyond this sample. All variables were measured using self-report questionnaires, potentially introducing social desirability and/or shared-method bias; the former was partially mitigated by patients returning questionnaires directly to the researchers, not their clinicians; the latter was partially mitigated by the inclusion of patient and practitioner completed questionnaires and the separation in time of the measurement of predictors and outcomes. Some of the ABS-MP subscales had low internal consistency in this sample (Cronbach's alphas <.6) suggesting the results of those analyses may be unreliable. Empathy and perceived competence, potentially important contextual components associated with the therapeutic relationship,[71] were not directly captured by the measure of therapeutic alliance.

The current findings highlight the relative importance of the therapeutic alliance as a contextual predictor of back-related disability over time, compared to other contextual components. The minimum clinically-important difference for primary care patients with LBP has been calculated

For peer review only

as a standard 2-3 points[72] or a 30% reduction in RMDQ score from baseline.[73] Therefore, a one unit increase in patients' rating of the therapeutic alliance (task) and a two unit increase in practitioners' outcome expectancies would be associated with a clinically-important reduction in back-related disability for patients scoring 7 or less on the RMDQ at baseline. For patients scoring 8 to 15 points on the RMDQ at baseline, a two-unit increase in patients' rating of the therapeutic alliance (task) and a three-unit increase in practitioners' outcome expectancies may be needed to make a clinically-important difference.

Our findings support a growing emphasis on patient-practitioner communication and relationships in musculoskeletal healthcare interactions. Previous studies and reviews have also suggested the importance of the therapeutic relationship for positive patient outcomes[7 10] but have not considered the extensive selection of other contextual variables measured here. A recent systematic review in physiotherapy concluded there was a lack of evidence of a strong relationship between therapeutic alliance and reduced pain, but the studies reviewed were all much smaller than the current cohort (n=12-182) and may have been underpowered.[74] The only other contextual factor to approach a moderate effect size on patient outcomes was the practitioners' outcome expectancies at baseline, which predicted outcomes even after controlling for baseline clinical and socio-demographic variables. This factor has been somewhat overlooked in the literature but was also found to predict outcomes in a large acupuncture study.[23] The reasons for this association likely involve practitioners making global clinical judgements that incorporate factors not measured in the current study. The statistically significant association between lower perceived treatment credibility and improved patient outcomes was not in the predicted direction; the effect size was very small (.01) and unlikely to be clinically significant. It may be that patients who believe their treatment is extremely credible are more likely to then experience some disappointment when experiencing the reality of that treatment, which may then impact engagement with treatment and/or outcome reporting. The lack of association between patients' expectancies and subsequent outcomes was unexpected given the balance of previous findings linking patient expectancies to outcomes,[9 75] but

For peer review only

not unprecedented: expectancies are more strongly and consistently associated with experimental acute and procedural pain than chronic pain.[11] This null finding may be related to timing, as expectancies may change over time[76] and patients can be reluctant to specify expectancies about effectiveness early in treatment, while concepts such as hope may have greater resonance in clinical settings.[77] The lack of associations between patient outcomes and practitioner perceptions of the healthcare environment, attitudes and orientation to LBP was also unexpected, but may be an artefact of the low reliability of the ABS-MP subscales.

Future research, using qualitative methods, could explore how practitioners form positive outcome expectancies and how this might be harnessed in practice. The possible causal nature of and mechanisms by which practitioner outcome expectancies impact patient outcomes also require further research. To confirm the clinical importance of therapeutic alliance and enable practitioners to harness this in practice, sensible next steps would be to develop and then trial brief and engaging post-qualification training for practitioners, using a systematic approach such as the person-based approach to intervention development.[78]

Having a strong therapeutic alliance and positive practitioner expectancies of outcome were the strongest contextual predictors of reduced disability in a large cohort of patients with LBP receiving physiotherapy, osteopathy and acupuncture. These operate in part by altering known psychological predictors of outcome: self-efficacy, perceived pain threat, and psychosocial distress. Enhancing these contextual components in practice could improve patient health outcomes from LBP. While a strong therapeutic alliance concerning the ultimate goals of treatment and a strong affective bond are associated with improved patient health outcomes, it is the strength of alliance concerning how to achieve the goals (i.e., the tasks of treatment) that has the largest effect on improved outcomes. The lack of between-treatment differences in significant predictors suggests the potential for shared learning across therapies to improve contextual components. Therefore, it is advisable for clinicians to focus on strengthening their alliance building related to how the patient

For peer review only

should reach an agreed-upon goal within the context of a strong affective bond. Evidence-based, engaging, training could be developed to support skills in therapeutic alliance building.

For peer review only

FUNDING STATEMENT

This work was supported by Arthritis Research UK Special Strategic Award grant number 20552.

AUTHOR CONTRIBUTIONS

FLB led the study conception and design and drafted the manuscript. FLB, KB, HM, LY, and LR conceptualised the study and drafted the protocol and the associated grant application. FLB, KB, HM, LY, MA, DC, CF, and LR drafted the manuscript. MA was the research fellow on the study, responsible for data collection under the supervision of FLB and KB and in collaboration with all authors. BS undertook the statistical analysis. All authors revised the manuscript critically for important intellectual content and gave final approval of the version to be published.

ACKNOWLEDGEMENTS

We would like to acknowledge the vital contribution that three, now deceased, colleagues made to the design and management of this research: Dr Borislav Dimitrov (University of Southampton), Dr Jan Leach (University of Brighton), and Professor George Lewith (University of Southampton). Specifically, George Lewith, Borislav Dimitrov, and Jan Leach contributed to conceptualising the study, drafting the protocol and the associated grant application, and supervising data collection.

We would like to thank all the practitioners and patients who took part in this study. The Clinical Research Network, British Acupuncture Council, Chartered Society of Physiotherapy, General Osteopathic Council, and the Institute of Osteopathy assisted with recruitment. Research and administrative assistance was provided by: Laura Wilde, Sue Eardley, Maddy Greville-Harris, J Matthew Harvey, Daniel Scanlan, Ricky Wong, Tasnia Priti, Daniela Baramova, Helen Loader, Nicole Collaco, Yasmin Hoque, and Senada Merkulaj. Our Patient Public Involvement collaborator was Jo Foster.

COMPETING INTERESTS STATEMENT

FLB received an honorarium and travel expenses for presenting the preliminary results of this research at the Acupuncture Research Resource Centre research symposium and has also received speaker's fees and travel expenses from the Acupuncture Association of Chartered Physiotherapists. The authors declare that they have no other competing interests.

DATA SHARING STATEMENT

Deidentified participant data may be requested from the corresponding author, Dr Felicity Bishop (https://orcid.org/0000-0002-8737-6662). Access may only be permitted for the purposes of checking the reported analyses and an analysis plan must be submitted with any request for access to the data. Due to ethical considerations, reuse for any other purpose is not permitted.

For peer review only

REFERENCES

1. Crum AJ, Corbin WR, Brownell KD, et al. Mind over milkshakes: mindsets, not just nutrients, determine ghrelin response. *Health Psychol* 2011;30(4):424-9; discussion 30-1. doi: 10.1037/a0023467 [published Online First: 2011/05/18]
2. Crum AJ, Zuckerman B. Changing mindsets to enhance treatment effectiveness. *JAMA* 2017;317(20):2063-64. doi: 10.1001/jama.2017.4545
3. GBD 2015 Disease and Injury Incidence and Prevalence Collaborators. Global, regional, and national incidence, prevalence, and years lived with disability for 310 diseases and injuries, 1990-2015: a systematic analysis for the Global Burden of Disease Study 2015. *Lancet* 2016;388(10053):1545-602. doi: 10.1016/S0140-6736(16)31678-6
4. Di Blasi Z, Harkness E, Ernst E, et al. Influence of context effects on health outcomes: a systematic review. *Lancet* 2001;357:757-62.
5. Roter DL. Which facets of communication have strong effects on outcome - a meta analysis. In: Stewart MA, Roter DL, eds. Communicating with Medical Patients. Newbury Park, CA: Sage Publications 1989.
6. Martin DJ, Garske JP, Davis MK. Relation of the therapeutic alliance with outcome and other variables: A meta-analytic review. *J Consult Clin Psychol* 2000;68(3):438-50.
7. Hall AM, Ferreira PH, Maher CG, et al. The Influence of the Therapist-Patient Relationship on Treatment Outcome in Physical Rehabilitation: A Systematic Review. *Phys Ther* 2010;90(8):1099-110.
8. Kaptchuk TJ, Kelley JM, Conboy LA, et al. Components of placebo effect: randomised controlled trial in patients with irritable bowel syndrome. *Br Med J* 2008;336:999-1003.
9. Howick J, Moscrop A, Mebius A, et al. Effects of empathic and positive communication in healthcare consultations: a systematic review and meta-analysis. *J R Soc Med* 2018;111(7):240-52. doi: 10.1177/0141076818769477 [published Online First: 2018/04/20]
10. Draper-Rodi J, Vogel S, Bishop A. Identification of prognostic factors and assessment methods on the evaluation of non-specific low back pain in a biopsychosocial environment: A scoping review. *International Journal of Osteopathic Medicine* 2018;30:25-34. doi: 10.1016/j.ijosm.2018.07.001
11. Peerdeman KJ, van Laarhoven AIM, Keij SM, et al. Relieving patients' pain with expectation interventions: a meta-analysis. *Pain* 2016;157(6):1179-91. doi: 10.1097/j.pain.0000000000000540
12. Devilly GJ, Borkovec TD. Psychometric properties of the credibility/expectancy questionnaire. *J Behav Ther Exp Psychiatry* 2000;31:73-86.
13. Crow R, Gage H, Hampson S, et al. The role of expectancies in the placebo effect and their use in the delivery of health care: a systematic review. *Health Technol Assess* 1999;3(3)
14. Colagiuri B, Smith CA. A systematic review of the effect of expectancy on treatment responses to acupuncture. *Evid Based Complement Alternat Med* 2012:doi:10.1155/2012/857804.
15. Bishop FL, Yardley L, Prescott P, et al. Psychological covariates of longitudinal changes in back-related disability in patients undergoing acupuncture. *Clin J Pain* 2015;31(3):254-64. doi: 10.1097/ajp.0000000000000108 [published Online First: 2014/06/06]
16. Kong J, Wang Z, Leiser J, et al. Enhancing treatment of osteoarthritis knee pain by boosting expectancy: A functional neuroimaging study. *Neuroimage-Clinical* 2018;18:325-34. doi: 10.1016/j.nicl.2018.01.021
17. Pincus T, Foster NE, Vogel S, et al. Attitudes to back pain amongst musculoskeletal practitioners: A comparison of professional groups and practice settings using the ABS-mp. *Manual Therapy* 2007;12(2):167-75.
18. Bishop A, Foster NE, Thomas E, et al. How does the self-reported clinical management of patients with low back pain relate to the attitudes and beliefs of health care practitioners? A survey of UK general practitioners and physiotherapists. *Pain* 2008;135:187-95.

For peer review only

19. Houben RMA, Ostelo RWJG, Vlaeyen JWS, et al. Health care providers' orientations towards common low back pain predict perceived harmfulness of physical activities and recommendations regarding return to normal activity. *European Journal of Pain* 2005;9(2):173-83. doi: 10.1016/j.ejpain.2004.05.002
20. Simmonds MJ, Derghazarian T, Vlaeyen JWS. Physiotherapists' Knowledge, Attitudes, and Intolerance of Uncertainty Influence Decision Making in Low Back Pain. *The Clinical Journal of Pain* 2012;28(6):467-74. doi: 10.1097/AJP.0b013e31825bfe65
21. Thomson OP, Petty NJ, Moore AP. Osteopaths' professional views, identities and conceptions – A qualitative grounded theory study. *International Journal of Osteopathic Medicine* 2014;17(3):146-59. doi: https://doi.org/10.1016/j.ijosm.2013.12.002
22. Hughes JG, Goldbart J, Fairhurst E, et al. Exploring acupuncturists' perceptions of treating patients with rheumatoid arthritis. *Complement Ther Med* 2007;15(2):101-08.
23. Witt CM, Martins F, Willich SN, et al. Can I help you? Physicians' expectations as predictor for treatment outcome. *European Journal of Pain* 2012;16(10):1455-66.
24. Linda HA, Walter S, Koen VdH, et al. Patient safety, satisfaction, and quality of hospital care: cross sectional surveys of nurses and patients in 12 countries in Europe and the United States. *Br Med J* 2012;344
25. Drahota A, Ward D, Mackenzie H, et al. Sensory environment on health-related outcomes of hospital patients (Review). *Cochrane Database of Systematic Reviews* 2012;3:Art. No.: CD005315. DOI: 10.1002/14651858.CD005315.pub2.
26. Bishop FL, Barlow F, Coghlan B, et al. Patients as healthcare consumers in the public and private sectors: a qualitative study of acupuncture in the UK. *BMC Health Serv Res* 2011;11(129):http://www.biomedcentral.com/1472-6963/11/129.
27. Bishop FL, Amos N, Yu H, et al. Health-care sector and complementary medicine: practitioners' experiences of delivering acupuncture in the public and private sectors. *Prim Health Care Res Dev* 2012;13(03):269-78.
28. Bradbury KJ, Bishop FL, Yardley L, et al. Patients' appraisals of public and private healthcare: A qualitative study of physiotherapy and osteopathy. *J Health Psychol* 2013;18(10):1307-18.
29. Evans M, Paterson C, Wye L, et al. Lifestyle and self-care advice within traditional acupuncture consultations: A qualitative observational study nested in a co-operative inquiry. *J Altern Complement Med* 2011;17(6):519-29. doi: 10.1089/acm.2010.0749.
30. Paterson C, Evans M, Bertschlinger R, et al. Communication about self-care in traditional acupuncture consultations: The co-construction of individualised support and advice. *Patient Educ Couns* 2012;89(3):467-75.
31. Haanstra TM, Kamper SJ, Williams CM, et al. Does adherence to treatment mediate the relationship between patients' treatment outcome expectancies and the outcomes of pain intensity and recovery from acute low back pain? *Pain* 2015;156(8):1530-36. doi: 10.1097/j.pain.0000000000000198
32. Agarwal V. Re-conceptualizing pain through patient-centred care in the complementary and alternative medicine therapeutic relationship. *J Adv Nurs* 2018;74(10):2406-15. doi: 10.1111/jan.13734
33. Carriere JS, Martel MO, Meints SM, et al. What do you expect? Catastrophizing mediates associations between expectancies and pain-facilitatory processes. *European journal of pain (London, England)* 2018 doi: 10.1002/ejp.1348
34. Vlaeyen JW, Linton SJ. Fear-avoidance model of chronic musculoskeletal pain: 12 years on. *Pain* 2012;153(6):1144-7. doi: 10.1016/j.pain.2011.12.009 [published Online First: 2012/02/11]
35. Lo GH-W, Balasubramanyam AS, Barbo A, et al. Link Between Positive Clinician-Conveyed Expectations of Treatment Effect and Pain Reduction in Knee Osteoarthritis, Mediated by Patient Self-Efficacy. *Arthritis Care Res* 2016;68(7):952-57. doi: 10.1002/acr.22775
36. Bandura A. Self-efficacy: toward a unifying theory of behavioral change. *Psychol Rev* 1977;84(2):191-215. [published Online First: 1977/03/01]

For peer review only

37. Carlino E, Benedetti F. Different contexts, different pains, different experiences. *Neuroscience* 2016;338:19-26. doi: https://doi.org/10.1016/j.neuroscience.2016.01.053
38. Lee I-S, Wallraven C, Kong J, et al. When pain is not only pain: Inserting needles into the body evokes distinct reward-related brain responses in the context of a treatment. *Physiol Behav* 2015;140:148-55. doi: https://doi.org/10.1016/j.physbeh.2014.12.030
39. Fields HL. How expectations influence pain. *Pain* 2018;159(9):S3-S10. doi: 10.1097/j.pain.0000000000001272
40. Neumann M, Bensing J, Mercer S, et al. Analyzing the "nature" and "specific effectiveness" of clinical empathy: A theoretical overview and contribution towards a theory-based research agenda. *Patient Educ Couns* 2009;74(3):339-46.
41. Street, Jr., Makoul G, Arora NK, et al. How does communication heal? Pathways linking clinician-patient communication to health outcomes. *Patient Educ Couns* 2009;74(3):295-301.
42. Bradbury KJ, Al-Abbadey M, Carnes D, et al. Non-specific mechanisms in orthodox and CAM management of low back pain (MOCAM): theoretical framework and protocol for a prospective cohort study. *BMJ Open* 2016;6(5):e012209. doi: 10.1136/bmjopen-2016-012209
43. Vickers AJ, Vertosick EA, Lewith G, et al. Acupuncture for Chronic Pain: Update of an Individual Patient Data Meta-Analysis. *J Pain* 2018;19(5):455-74. doi: 10.1016/j.jpain.2017.11.005
44. Breivik H, Collett B, Ventafridda V, et al. Survey of chronic pain in Europe: Prevalence, impact on daily life, and treatment. *European Journal of Pain* 2006;10(4):287-333.
45. Rubinstein SM, de Zoete A, van Middelkoop M, et al. Benefits and harms of spinal manipulative therapy for the treatment of chronic low back pain: systematic review and meta-analysis of randomised controlled trials. *BMJ* 2019;364:l689. doi: 10.1136/bmj.l689 [published Online First: 2019/03/15]
46. Oliveira CB, Maher CG, Pinto RZ, et al. Clinical practice guidelines for the management of non-specific low back pain in primary care: an updated overview. *Eur Spine J* 2018;27(11):2791-803. doi: 10.1007/s00586-018-5673-2
47. Qaseem A, Wilt TJ, McLean RM, et al. Noninvasive Treatments for Acute, Subacute, and Chronic Low Back Pain: A Clinical Practice Guideline From the American College of Physicians. *Ann Intern Med* 2017;166(7):514-30. doi: 10.7326/m16-2367
48. Foley H, Steel A. Patient perceptions of clinical care in complementary medicine: A systematic review of the consultation experience. *Patient Educ Couns* 2017;100(2):212-23. doi: 10.1016/j.pec.2016.09.015 [published Online First: 2016/10/04]
49. Kaptchuk TJ, Stason WB, Davis RB, et al. Sham device v inert pill: randomised controlled trial of two placebo treatments. *Br Med J* 2006;332(7538):391-94.
50. Bishop FL, Lewis G, Harris S, et al. A within-subjects trial to test the equivalence of online and paper outcome measures: the Roland Morris disability questionnaire. *BMC Musculoskelet Disord* 2010;11:113. doi: 10.1186/1471-2474-11-113 [published Online First: 2010/06/10]
51. Roland M, Morris R. A study of the natural history of back pain. Part I: Development of a reliable and sensitive measure of disability in low-back pain. *Spine* 1983;8 (2):141-44.
52. Croft PR, Macfarlane GJ, Papageorgiou AC, et al. Outcome of low back pain in general practice: a prospective study. *Br Med J* 1998;316:1356.
53. Hestbaek L, Leboeuf-Yde C, Engberg M, et al. The course of low back pain in a general population. results from a 5-year prospective study. *J Manipulative Physiol Ther* 2003;26(4):213-19.
54. Robinson KA, Dinglas VD, Sukrithan V, et al. Updated systematic review identifies substantial number of retention strategies: using more strategies retains more study participants. *J Clin Epidemiol* 2015;68(12):1481-7. doi: 10.1016/j.jclinepi.2015.04.013 [published Online First: 2015/07/19]
55. Deyo RA, Battie M, Beurskens AJHM, et al. Outcome measures for low back pain research: A proposal for standardized use. *Spine* 1998;23(18):2003-13.

For peer review only

56. Horvath AO, Greenberg LS. Development and validation of the Working Alliance Inventory. *Journal of Counseling Psychology* 1989;36(2):223-33.
57. Busseri MA, Tyler JD. Interchangeability of the Working Alliance Inventory and Working Alliance Inventory, Short Form. *Psychological Assessment* 2003;15(2):193-97.
58. Pincus T, Vogel S, Santos R, et al. The Attitudes to Back Pain Scale in Musculoskeletal Practitioners (ABS-mp): The Development and Testing of a New Questionnaire. *Clin J Pain* 2006;22(4):378-86.
59. Grogan S, Conner M, Norman P, et al. Validation of a questionnaire measuring patient satisfaction with general practitioner services. *Quality and Safety in Health Care* 2000;9(4):210-15.
60. Dima A, Lewith GT, Little P, et al. Patients' treatment beliefs in low back pain: development and validation of a questionnaire in primary care. *Pain* 2015;156(8):1489-500. doi: 10.1097/j.pain.0000000000000193 [published Online First: 2015/04/24]
61. Broadbent E, Petrie KJ, Main J, et al. The Brief Illness Perception Questionnaire. *J Psychosom Res* 2006;60:631-37.
62. Anderson KO, Dowds BN, Pelletz RE, et al. Development and initial validation of a scale to measure self-efficacy beliefs in patients with chronic pain. *Pain* 1995;63(1):77-83.
63. Hill JC, Dunn KM, Lewis M, et al. A primary care back pain screening tool: Identifying patient subgroups for initial treatment. *Arthritis Care Res* 2008;59(5):632-41.
64. Dunn KM, Croft PR. Classification of Low Back Pain in Primary Care: Using "Bothersomeness" to Identify the Most Severe Cases. *Spine* 2005;30(16):1887-92.
65. Department for Communities and Local Government. The English Index of Multiple Deprivation (IMD) 2015–Guidance. Gov.UK, 2015.
66. Farrin A, Russell I, Torgerson D, et al. Differential recruitment in a cluster randomized trial in primary care: the experience of the UK back pain, exercise, active management and manipulation (UK BEAM) feasibility study. *Clin Trials* 2005;2(2):119-24. [published Online First: 2005/11/11]
67. Rules of thumb on magnitudes of effect sizes. MRC CBU Wiki. http://imaging.mrc-cbu.cam.ac.uk/statswiki/FAQ/effectSize. Date accessed: February 2021.
68. Baron RM, Kenny DA. The Moderator Mediator Variable Distinction in Social Psychological-Research - Conceptual, Strategic, and Statistical Considerations. *J Pers Soc Psychol* 1986;51(6):1173-82.
69. Brett J, Staniszewska S, Mockford C, et al. Mapping the impact of patient and public involvement on health and social care research: a systematic review. *Health Expect* 2014;17(5):637-50. doi: 10.1111/j.1369-7625.2012.00795.x
70. Wiering B, de Boer D, Krol M, et al. Entertaining accurate treatment expectations while suffering from chronic pain: an exploration of treatment expectations and the relationship with patient- provider communication. *BMC Health Serv Res* 2018;18 doi: 10.1186/s12913-018-3497-8
71. Howe LC, Goyer JP, Crum AJ. Harnessing the placebo effect: Exploring the influence of physician characteristics on placebo response. *Health Psychol* 2017;36(11):1074-82. doi: 10.1037/hea0000499 [published Online First: 2017/03/09]
72. Roland M, Fairbank J. The Roland Morris Disability Questionnaire and the Oswestry Disability Questionnaire. *Spine* 2000;25:3115-24.
73. Jordan K, Dunn KM, Lewis M, et al. A minimal clinically important difference was derived for the Roland-Morris Disability Questionnaire for low back pain. *J Clin Epidemiol* 2006;59(1):45-52. doi: https://doi.org/10.1016/j.jclinepi.2005.03.018
74. Taccolini Manzoni AC, Bastos de Oliveira NT, Nunes Cabral CM, et al. The role of the therapeutic alliance on pain relief in musculoskeletal rehabilitation: A systematic review. *Physiotherapy Theory and Practice* 2018;34(12):901-15. doi: 10.1080/09593985.2018.1431343

For peer review only

75. Peerdeman KJ, van Laarhoven AM, Peters ML, et al. An Integrative Review of the Influence of Expectancies on Pain. *Frontiers in Psychology* 2016;7 doi: 10.3389/fpsyg.2016.01270
76. Eaves ER, Sherman KJ, Ritenbaugh C, et al. A qualitative study of changes in expectations over time among patients with chronic low back pain seeking four CAM therapies. *BMC Complement Altern Med* 2015;15 doi: 10.1186/s12906-015-0531-9
77. Bishop FL, Lewith GT. Patients' preconceptions of acupuncture: a qualitative study exploring the decisions patients make when seeking acupuncture. *BMC Complement Altern Med* 2013;13:102. doi: DOI: 10.1186/1472-6882-13-102
78. Yardley L, Morrison L, Bradbury K, Muller I. The person-based approach to intervention development: application to digital health-related behavior change interventions. *J Med Internet Res* 2015;17(1):e30. doi:10.2196/jmir.4055.

For peer review only

Figure Captions

Figure 1. Modified Consort Diagram. Showing Participant Flow, Recruitment and Drop-Out.

Figure 2. Back-Related Disability over Time. Mean scores and standard errors are plotted.

For peer review only

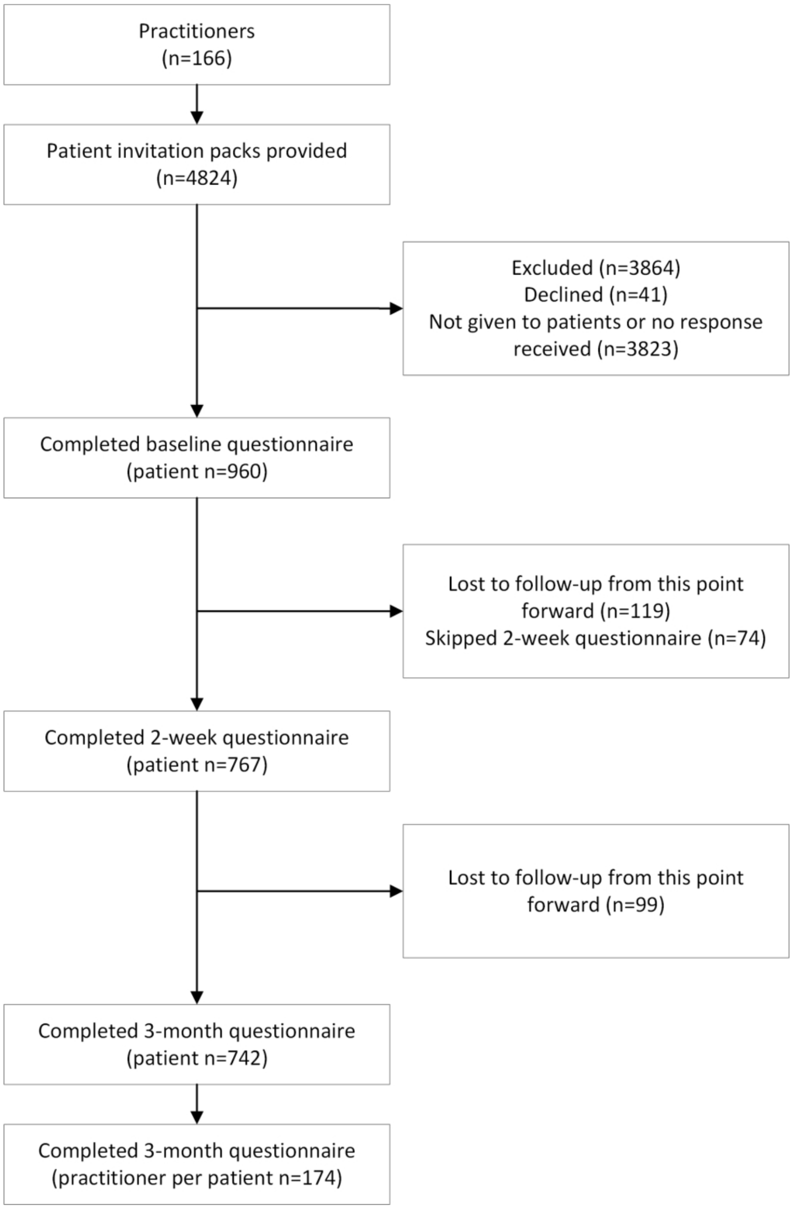

Modified Consort Diagram. Showing Participant Flow, Recruitment and Drop-Out.

147x224mm (300 x 300 DPI)

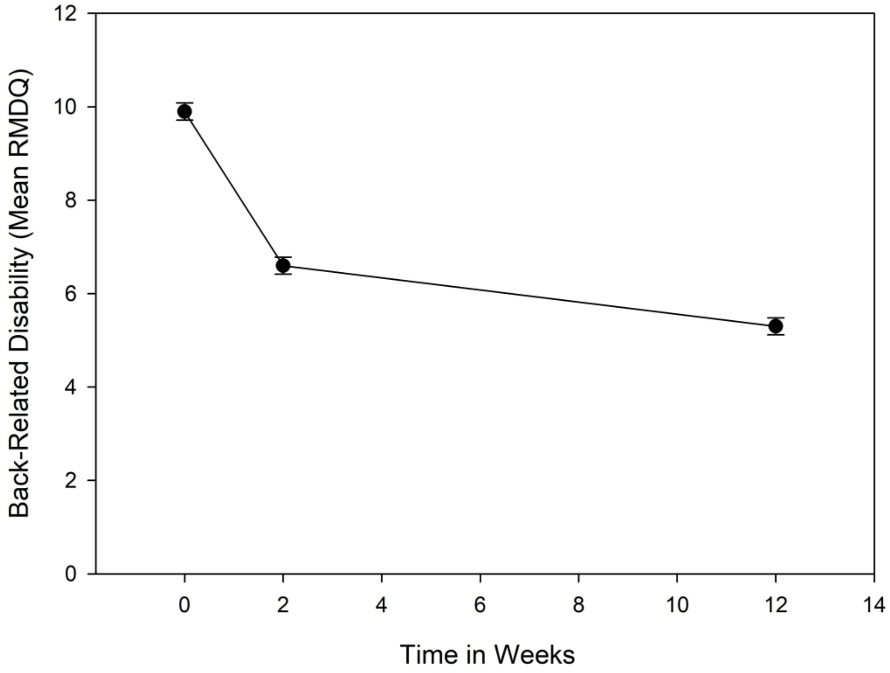

Figure 2. Back-Related Disability over Time. Mean scores and standard errors are plotted.

1003x761mm (96 x 96 DPI)

Direct and Mediated Effects of Treatment Context on Back Pain: Supplemental Material

Contents

Table 1: Deviations from Published Protocol 1
Table 2: Study Measures with Example Items, Response Scales, and Interpretation 2
Table 3. Treatment Characteristics by Treatment Group, n (%) 6
Table 4. Practitioner Characteristics at Baseline by Treatment Group 7
Table 5. Secondary Outcomes (Mean, SD) over Time. 8
Table 6. Mean (Standard Deviation) Scores on Mediators over Time by Treatment Group 9
Table 7. Interaction Terms between Contextual Factors and Treatment Type 10
Figure 1. Modified Consort Diagram. Showing Participant Flow, Recruitment and Drop-Out by Therapy Group. 12
Figure 2. Primary Outcomes over Time by Therapy Group 14
Figure 3. General Mediation Model 15
References 16

For peer review only

Table 1: Deviations from Published Protocol

#	Deviation	Rationale
1	Unable to recruit equivalent numbers of practitioners and patients from the NHS and the private sector and therefore did not test the hypothesis that differences in contextual effects between acupuncture, osteopathy, and physiotherapy would be more pronounced in the NHS than the private sector.	Around the time recruitment began there was a marked decline in NHS commissioning of osteopathy and acupuncture for low back pain. This made it very difficult to recruit these groups.
2	Revised sample size calculation.	Necessary to reflect changes in sample as per deviation #1.
3	No test of whether effects of contextual components on back-related disability are mediated by increased patient adherence to theory-specific lifestyle advice (in addition to pain beliefs and self-efficacy).	Extensive missing patient self-report data on adherence and the variety of lifestyle advice precluded a test of this hypothesis.
4	Practitioner-reported data on caseload, waiting list, and appointment duration not collected.	Items were erroneously omitted from the questionnaire.

For peer review only - http://bmjopen.bmj.com/site/about/guidelines.xhtml

Table 2: Study Measures with Example Items, Response Scales, and Interpretation

Construct	Measure (subscale)	Example item	Response scale	Possible range	Cronbach's alpha**	High score indicates
Primary Outcomes						
Disability	RMDQ[1]	I change position frequently to try to get my back comfortable	Yes/No	0-24	-	Greater back-related disability
Secondary Outcomes						
Social Role Disability	Core single item[2]	During the past 4 weeks, how many days did back pain or leg pain (sciatica) keep you from going to work or college?[2]	Free text – number of days in past 4 weeks	0-28	-	Greater social role disability
Functional impairment	Core single item[2]	During the past week, how much did pain interfere with your normal work (including both work outside the home and housework)?[2]	5-point verbal	1-5	-	Greater functional impairment
Pain	Core single item[2]	Please rate your pain by indicating the number that best describes your pain on average in the last 24 hours.[2]	11-point numerical	0-10	-	Greater average pain over last 24 hours
Wellbeing	Core single item[2]	If you had to spend the rest of your life with the symptoms you have right now, how would you feel about it?	5-point verbal	1-5	-	Greater wellbeing
Satisfaction	Core single item[2]	Over the course of treatment for your back pain or leg pain (sciatica) so far, how satisfied are you with your overall health care?	5-point verbal	1-5	-	Greater satisfaction with treatment
Contextual Components						
Therapeutic alliance: task	WAI-SF[3 4] (Task)	I believe the way we are working with my problem is correct	5-point verbal	1-5	0.67	Greater patient-practitioner agreement on task (i.e. how to

For peer review only

						achieve goals)
Therapeutic alliance: goal	WAI-SF[3,4] (Goal)	___ and I are working towards mutually agreed upon goals	5-point verbal	1-5	0.74	Greater patient-practitioner agreement on goals
Therapeutic alliance: bond	WAI-SF[3,4] (Bond)	I believe ___ likes me.	5-point verbal	1-5	0.86	Greater patient-practitioner interpersonal affective bond
Environment: Limits on Treatment*	ABS-mp[5,6] (Limitations on sessions)	If I keep seeing my patients on and off I can prevent relapse	7-point agree/disagree	4-28	0.46	Disinclined to put a limit on number of sessions or refer elsewhere
Environment: Connections within the Healthcare System*	ABS-mp[5,6] (Perceived connections within the health care system)	When referring patients I am confident they will receive good treatment	7-point agree/disagree	3-21	0.49	Feels well-connected to health care system resources
Environment: Satisfaction with appointment systems	PSQ[7] (Appointments)	Getting an appointment at a convenient time is easy	5-point agree/disagree	1-5	0.81	Less satisfied with appointment systems
Environment: Satisfaction with access	PSQ[7] (Access)	It is easy to get advice over the telephone	5-point agree/disagree	1-5	0.80	Less satisfied with access
Environment: Satisfaction with facilities	PSQ[7] (Facilities)	The waiting room is uncomfortable	5-point agree/disagree	1-5	0.75	Less satisfied with facilities
Patient's beliefs: Expectancies for treatment effectiveness	LBP treatment beliefs questionnaire[8] (Effectiveness)	[therapy] can work well for people with back pain	5-point agree/disagree	1-5	0.82	Stronger beliefs that therapy will be effective
Patient's beliefs: Perceived credibility of treatment	LBP treatment beliefs questionnaire [8] (Credibility)	Having [therapy] for back pain makes a lot of sense	5-point agree/disagree	1-5	0.85	Stronger beliefs that therapy is credible
Patient's beliefs:	LBP treatment beliefs	I feel that [therapy] would not	5-point	1-5	0.83	Fewer concerns about

For peer review only - http://bmjopen.bmj.com/site/about/guidelines.xhtml

Concerns about treatment	questionnaire [8] (Concerns)	harm me	agree/disagree			therapy
Patient's beliefs: Individualised fit of treatment	LBP treatment beliefs questionnaire [8] (individual fit)	I am confident [therapy] would be a suitable treatment for my back pain	5-point agree/disagree	1-5	0.87	Stronger beliefs that therapy is good fit for oneself
Practitioner's beliefs: psychological*	ABS-mp [5,6] (Psychological)	I often find myself providing psychological support to patients	7-point agree/disagree	4-28	0.65	More willing to engage with patients' psychological issues
Practitioner's beliefs: confidence *	ABS-mp[5,6] (Confidence and concern)	I am concerned about the quality of treatment my referred patients receive	7-point agree/disagree	2-14	0.18	More confident and less concerned about one's own and others' clinical limitations
Practitioner's beliefs: reactivation*	ABS-mp[5,6] (Reactivation)	Return to normal daily activities is the most important consequence of treatment	7-point agree/disagree	3-21	0.57	Greater tendency to focus on re-activation of work and activity
Practitioner's beliefs: biomedical*	ABS-mp[5,6] (Biomedical)	If you look hard enough you can find a structural reason for most patients' back pain	7-point agree/disagree	3-21	0.57	Greater tendency to focus on biomedical model of back pain (also includes rest and vigilance)
Practitioner's beliefs: Outcome Expectancies*	Single item per patient	Thinking about this patient, how effective do you expect your course of treatment to be?	7-point numerical	1-7	-	Expects treatment to be extremely effective for this patient
Hypothesised Mediators						
Self-efficacy for pain management	Self-efficacy for pain management[9]	How certain are you that you can decrease your pain quite a bit?	10-point numerical	10-100	0.78	Higher self-efficacy for pain management
Perception of LBP as threatening	Brief IPQ[10]	How much does your back pain affect your life?	11-point numerical	0-80	0.73	LBP perceived as more threatening
Psychosocial distress	StartBack[11]	I feel that my back pain is terrible and it's never going to get any better	2-point agree/disagree	0-5	0.45	Greater psychosocial distress

* These questionnaires completed by practitioners. All others completed by patients.

For peer review only

** Cronbach's alpha in this sample

RMDQ, Roland Morris Disability Questionnaire. WAI-SF, Working Alliance Inventory – Short Form. ABS-mp, Attitudes to Back Pain Scale – Musculoskeletal Practitioners. PSQ, Patient Satisfaction Questionnaire. LBP, Low Back Pain. IPQ, Illness Perceptions Questionnaire.

For peer review only - http://bmjopen.bmj.com/site/about/guidelines.xhtml

Table 3. Treatment Characteristics by Treatment Group, n (%)

	Physiotherapy NHS (n=196)	Physiotherapy Private (n=165)	Osteopathy (n=394)	Acupuncture (n=205)
Number of weeks treated: M(SD)	6.9 (5.3)	6.1 (9.8)	4.3 (4.6)	8.6 (10.9)
Number of appointments attended: M (SD)	3.9 (2.7)	4.0 (3.2)	3.2 (2.3)	5.1 (4.2)
Treatment Modalities (>1 permitted)				
Manual therapy	61 (31.1%)	116 (70.3%)	248 (62.9%)	50 (24.4%)
Electro/thermal therapy	7 (3.6%)	30 (18.2%)	45 (11.4%)	59 (28.8%)
Acupuncture	21 (10.7%)	18 (10.9%)	46 (11.7%)	123 (60.0%)
Exercise	139 (70.9%)	117 (70.9%)	128 (32.5%)	16 (7.8%)
Lifestyle advice	127 (64.8%)	112 (67.9%)	205 (52.0%)	56 (27.3%)
Missing data	45 (23.0%)	44 (26.7%)	93 (23.6%)	80 (39.0%)

NHS, National Health Service. M, Mean. SD, Standard Deviation.

Note. The average duration of treatment was longest for patients seeing acupuncturists and shortest for patients seeing osteopaths. There was considerable missing data on the specific treatment modalities used for each patient (particularly for the acupuncture group). Among those patients for whom treatment modalities were reported, manual therapy techniques were most commonly used with patients consulting osteopaths and physiotherapists in the private sector; electro/thermal and acupuncture techniques were most likely to be applied by acupuncturists but were used for a minority of patients in the other groups; and patients consulting physiotherapists were most likely to undertake specific exercises or be given lifestyle advice. Categories were defined as follows: manual therapy (specifically, one or more of: mobilisation, articulation, grade V manipulation, high-velocity low-amplitude technique, soft tissue techniques, and East Asian techniques such as acupressure, tuina, gua sha, cupping); electro-therapy therapy (transcutaneous electrical nerve stimulation, ultrasound, electro-acupuncture, application of heat, application of cold, moxibustion); acupuncture (western acupuncture such as trigger point needling and segmental needling, traditionally-based acupuncture such as Traditional Chinese Medicine, five elements, stems and branches, Japanese needling); exercise (individual, group, hydrotherapy); lifestyle advice (therapy-specific and non-therapy-specific advice on diet, specific exercises, physical activity, rest, and relaxation).

For peer review only

Table 4. Practitioner Characteristics at Baseline by Treatment Group

	Whole sample (n=166)	Physiotherapy NHS (n=36)	Physiotherapy Private (n=29)	Osteopathy (n=46)	Acupuncture (n=55)
Years since qualifying	20.6 (10.1)	15.5 (7.2)	25.3 (11.0)	24.0 (10.9)	18.5 (8.6)
Self-rated experience treating LBP*	4.3 (0.8)	3.8 (1.3)	4.5 (0.9)	4.5 (0.7)	4.2 (0.7)
Age	49.7 (10.8)	39.2 (8.3)	50.8 (8.3)	55.0 (10.6)	53.3 (14.6)

* Rated on a 1-5 scale where 1 = not at all experienced; 5 = very experienced.
NHS, National Health Service. LBP, Low Back Pain.

For peer review only

For peer review only - http://bmjopen.bmj.com/site/about/guidelines.xhtml

Table 5. Secondary Outcomes (Mean, SD) over Time.

Secondary Outcome	Baseline	2-weeks	3-months
Social Role Disability	2.0 (5.4)	N/A	0.9 (4.2)
Functional impairment	3.2 (1.1)	2.5 (1.1)	2.2 (1.1)
Pain	5.0 (2.2)	3.5 (2.4)	3.4 (2.5)
Wellbeing	1.6 (0.9)	2.3 (1.3)	2.8 (1.4)
Satisfaction	4.1 (1.0)	4.1 (1.0)	4.1 (1.1)

For peer review only

Table 6. Mean (Standard Deviation) Scores on Mediators over Time by Treatment Group

		Self-efficacy for coping with pain			Perception of LBP as threatening			Psychosocial complexity of LBP		
Treatment Group / Timepoint		Baseline	2-weeks	3-months	Baseline	2-weeks	3-months	Baseline	2-weeks	3-months
Whole sample		68.5 (19.7)	65.9 (25.3)	69.1 (24.1)	37.2 (9.9)	32.7 (13.2)	32.1 (14.5)	0.60 (0.7)	1.1 (1.8)	1.1 (1.9)
Physiotherapy	NHS	60.6 (19.2)	56.2 (24.0)	59.0 (24.3)	41.0 (9.5)	38.9 (11.8)	36.8 (13.9)	0.6 (0.6)	1.3 (1.9)	1.4 (1.9)
Physiotherapy	Private	74.8 (23.0)	71.4 (26.6)	75.3 (24.6)	34.3 (9.4)	27.9 (13.4)	26.8 (14.0)	0.6 (0.6)	0.8 (1.6)	1.0 (1.9)
Osteopathy		70.0 (17.9)	66.8 (26.0)	71.4 (23.7)	35.4 (9.7)	30.7 (12.9)	30.0 (13.9)	0.6 (0.7)	1.1 (1.9)	1.0 (1.9)
Acupuncture		68.2 (18.3)	68.2 (21.9)	68.8 (21.7)	39.8 (9.4)	35.2 (12.4)	35.5 (14.5)	0.6 (0.7)	1.0 (1.7)	1.3 (1.9)

For peer review only - http://bmjopen.bmj.com/site/about/guidelines.xhtml

Table 7. Interaction Terms between Contextual Factors and Treatment Type

Contextual component	Interaction term (95% CI) Osteopathy*	Interaction term (95% CI) Acupuncture*
Therapeutic alliance: goal	-0.27 (-1.62, 1.09)	0.88 (-0.79, 2.55)
Therapeutic alliance: task	-0.32 (-1.64, 1.00)	1.26 (-0.41, 2.93)
Therapeutic alliance: bond	0.16 (-0.83, 1.15)	0.55 (-0.78, 1.89)
Environment: Limits on Treatment	-0.13 (-0.60, 0.34)	0.14 (-0.33, 0.62)
Environment: Connections within the Healthcare System	0.12 (-0.35, 0.59)	0.33 (-0.24, 0.91)
Environment: Satisfaction with appointment systems	-0.07 (-0.22, 0.36)	-0.20 (-0.68, 0.08)
Environment: Satisfaction with access	-0.15 (-0.36, 0.07)	-0.31 (-0.47, -0.06)
Environment: Satisfaction with facilities	-0.13 (-0.42, 0.16)	-0.46 (-0.82, 0.09)
Patient's beliefs: Perceived credibility of treatment	1.43 (-1.43, 4.30)	0.58 (-2.26, 3.43)
Patient's beliefs: Expectancies for treatment effectiveness	2.86 (-0.08, 5.80)	1.85 (-1.13, 4.83)
Patient's beliefs: Concerns about treatment	0.04 (-2.85, 2.92)	-0.53 (-3.14, 2.09)
Patient's beliefs: Individualised fit of treatment	0.58 (-2.46, 3.62)	1.92 (-1.13, 4.97)
Practitioner's beliefs: Psychological	-0.46 (-0.87, 0.04)	-0.43 (-1.08, 0.21)
Practitioner's beliefs: Confidence	-0.34 (-1.01, 0.33)	-0.19 (-0.94, 0.55)

For peer review only - http://bmjopen.bmj.com/site/about/guidelines.xhtml

Practitioner's beliefs: Reactivation	-0.17 (-0.57, 0.23)	0.18 (-0.24, 0.60)
Practitioner's beliefs: Biomedical	0.09 (-0.25, 0.43)	-4.10 (-9.50, 1.33)
Practitioner's beliefs: Outcome expectancies	0.22 (-0.61, 1.04)	0.37 (-0.61, 1.36)

*Physiotherapy is the reference group in the categorical treatment variable

For peer review only

For peer review only - http://bmjopen.bmj.com/site/about/guidelines.xhtml

Figure 1. Modified Consort Diagram. Showing Participant Flow, Recruitment and Drop-Out by Therapy Group.

For peer review only

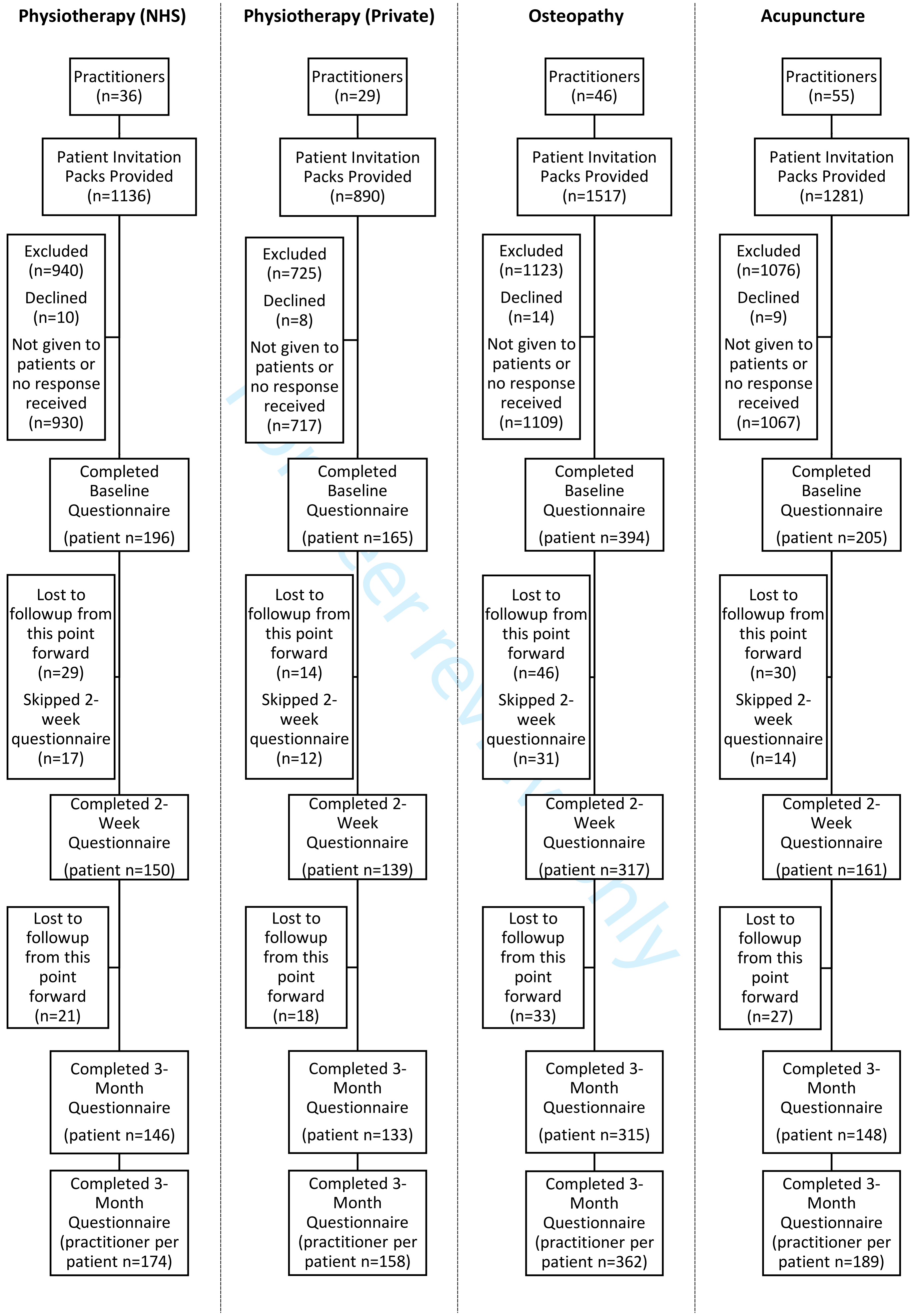

For peer review only - http://bmjopen.bmj.com/site/about/guidelines.xhtml

Figure 2. Primary Outcomes over Time by Therapy Group

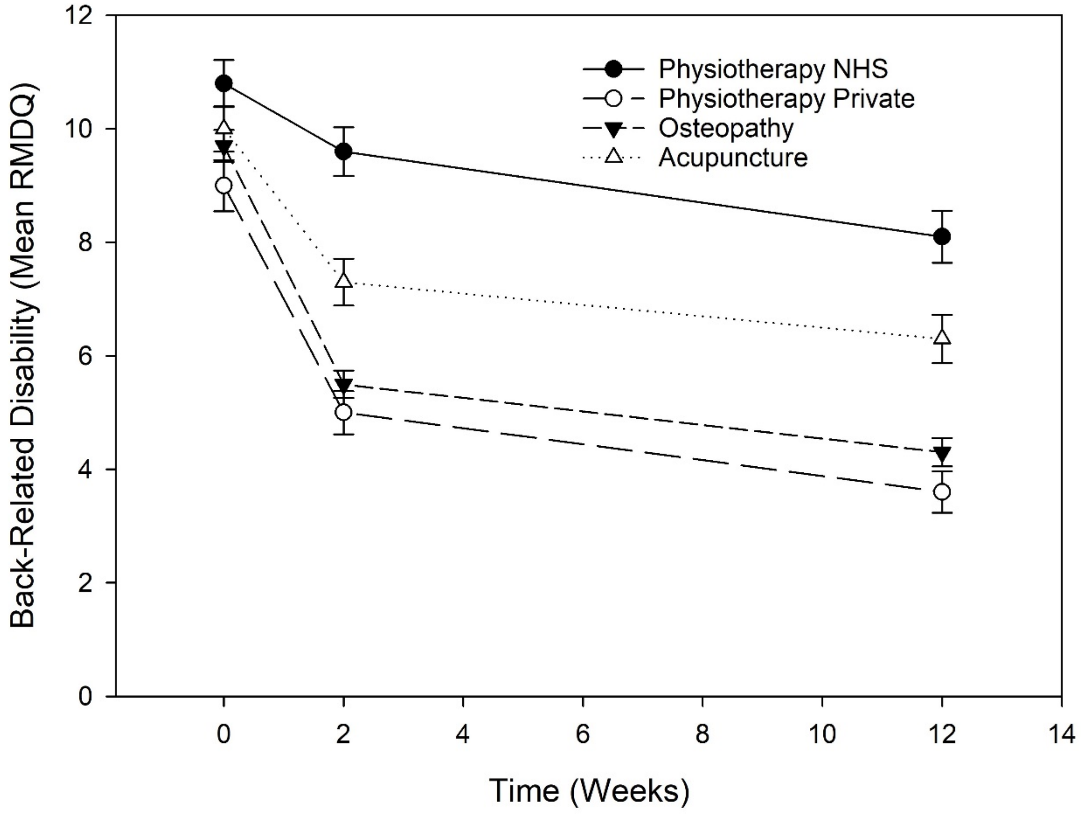

ew only

Figure 3. General Mediation Model

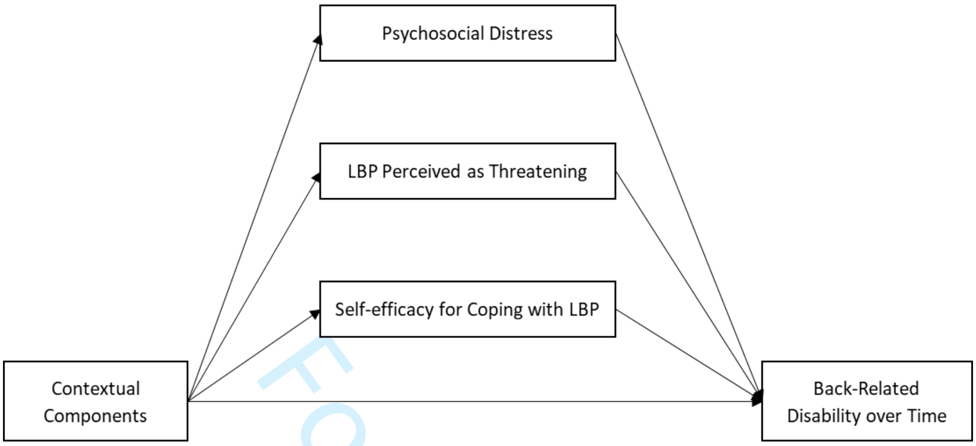

For peer review only

For peer review only - http://bmjopen.bmj.com/site/about/guidelines.xhtml

References

1. Roland M, Morris R. A study of the natural history of back pain. Part I: Development of a reliable and sensitive measure of disability in low-back pain. *Spine* 1983;8 (2):141-44.
2. Deyo RA, Battie M, Beurskens AJHM, et al. Outcome measures for low back pain research: A proposal for standardized use. *Spine* 1998;23(18):2003-13.
3. Busseri MA, Tyler JD. Interchangeability of the Working Alliance Inventory and Working Alliance Inventory, Short Form. *Psychological Assessment* 2003;15(2):193-97.
4. Tracey TJ, Kokotovic AM. Factor structure of the Working Alliance Inventory. *Psychological Assessment: A Journal of Consulting and Clinical Psychology* 1989;1(3):207-10.
5. Pincus T, Foster NE, Vogel S, et al. Attitudes to back pain amongst musculoskeletal practitioners: A comparison of professional groups and practice settings using the ABS-mp. *Manual Therapy* 2007;12(2):167-75.
6. Pincus T, Vogel S, Santos R, et al. The Attitudes to Back Pain Scale in Musculoskeletal Practitioners (ABS-mp): The Development and Testing of a New Questionnaire. *Clin J Pain* 2006;22(4):378-86.
7. Grogan S, Conner M, Norman P, et al. Validation of a questionnaire measuring patient satisfaction with general practitioner services. *Quality and Safety in Health Care* 2000;9(4):210-15.
8. Dima A, Lewith GT, Little P, et al. Patients' treatment beliefs in low back pain: development and validation of a questionnaire in primary care. *Pain* 2015;156(8):1489-500. doi: 10.1097/j.pain.0000000000000193 [published Online First: 2015/04/24]
9. Anderson KO, Dowds BN, Pelletz RE, et al. Development and initial validation of a scale to measure self-efficacy beliefs in patients with chronic pain. *Pain* 1995;63(1):77-83.
10. Broadbent E, Petrie KJ, Main J, et al. The Brief Illness Perception Questionnaire. *J Psychosom Res* 2006;60:631-37.
11. Hill JC, Dunn KM, Lewis M, et al. A primary care back pain screening tool: Identifying patient subgroups for initial treatment. *Arthritis Care Res* 2008;59(5):632-41.

For peer review only

For peer review only - http://bmjopen.bmj.com/site/about/guidelines.xhtml

STROBE Statement—Checklist of items that should be included in reports of ***cohort studies***

	Item No	Recommendation	
**Title and abstract**	1	(*a*) Indicate the study's design with a commonly used term in the title or the abstract	✓ p1
		(*b*) Provide in the abstract an informative and balanced summary of what was done and what was found	✓ p2
**Introduction**			
Background/rationale	2	Explain the scientific background and rationale for the investigation being reported	✓ p4-5
Objectives	3	State specific objectives, including any prespecified hypotheses	✓ p5-6
**Methods**			
Study design	4	Present key elements of study design early in the paper	✓ p6
Setting	5	Describe the setting, locations, and relevant dates, including periods of recruitment, exposure, follow-up, and data collection	✓ p6-7
Participants	6	(*a*) Give the eligibility criteria, and the sources and methods of selection of participants. Describe methods of follow-up	✓ p7-8
		(*b*) For matched studies, give matching criteria and number of exposed and unexposed	N/A
Variables	7	Clearly define all outcomes, exposures, predictors, potential confounders, and effect modifiers. Give diagnostic criteria, if applicable	✓ p8-11
Data sources/ measurement	8*	For each variable of interest, give sources of data and details of methods of assessment (measurement). Describe comparability of assessment methods if there is more than one group	✓ p8-11
Bias	9	Describe any efforts to address potential sources of bias	✓ p12, p22
Study size	10	Explain how the study size was arrived at	✓ p11
Quantitative variables	11	Explain how quantitative variables were handled in the analyses. If applicable, describe which groupings were chosen and why	✓
Statistical methods	12	(*a*) Describe all statistical methods, including those used to control for confounding	✓ p12-13
		(*b*) Describe any methods used to examine subgroups and interactions	✓ p12-13
		(*c*) Explain how missing data were addressed	✓ p12-13
		(*d*) If applicable, explain how loss to follow-up was addressed	✓ p12-13
		(*e*) Describe any sensitivity analyses	N/A
**Results**			
Participants	13*	(a) Report numbers of individuals at each stage of study—eg numbers potentially eligible, examined for eligibility, confirmed eligible, included in the study, completing follow-up, and analysed	✓ Fig 1
		(b) Give reasons for non-participation at each stage	✓ Fig 1
		(c) Consider use of a flow diagram	✓ Fig 1
Descriptive data	14*	(a) Give characteristics of study participants (eg demographic, clinical, social) and information on exposures and potential confounders	✓ Table

For peer review only - http://bmjopen.bmj.com/site/about/guidelines.xhtml

			1
		(b) Indicate number of participants with missing data for each variable of interest	✓ Table 1
		(c) Summarise follow-up time (eg, average and total amount)	✓ p17
Outcome data	15*	Report numbers of outcome events or summary measures over time	✓ p17
Main results	16	(*a*) Give unadjusted estimates and, if applicable, confounder-adjusted estimates and their precision (eg, 95% confidence interval). Make clear which confounders were adjusted for and why they were included	✓ Table 2
		(*b*) Report category boundaries when continuous variables were categorized	✓ Table 1
		(*c*) If relevant, consider translating estimates of relative risk into absolute risk for a meaningful time period	N/A
Other analyses	17	Report other analyses done—eg analyses of subgroups and interactions, and sensitivity analyses	✓ p18
**Discussion**			
Key results	18	Summarise key results with reference to study objectives	✓ p21
Limitations	19	Discuss limitations of the study, taking into account sources of potential bias or imprecision. Discuss both direction and magnitude of any potential bias	✓ p22
Interpretation	20	Give a cautious overall interpretation of results considering objectives, limitations, multiplicity of analyses, results from similar studies, and other relevant evidence	✓ p22-25
Generalisability	21	Discuss the generalisability (external validity) of the study results	✓ p22
**Other information**			
Funding	22	Give the source of funding and the role of the funders for the present study and, if applicable, for the original study on which the present article is based	✓ p26

*Give information separately for exposed and unexposed groups.

Note: An Explanation and Elaboration article discusses each checklist item and gives methodological background and published examples of transparent reporting. The STROBE checklist is best used in conjunction with this article (freely available on the Web sites of PLoS Medicine at http://www.plosmedicine.org/, Annals of Internal Medicine at http://www.annals.org/, and Epidemiology at http://www.epidem.com/). Information on the STROBE Initiative is available at http://www.strobe-statement.org.

For peer review only
